# Supplementary material for: Modular antibodies reveal DNA damage-induced mono-ADP-ribosylation as a second wave of PARP1 signaling
Source: Mol Cell. Author manuscript; Available in PMC 2025 Apr 24. (PMC10205078; doi:10.1016/j.molcel.2023.03.027)
Supplement: Supplemental Information [file NIHMS1899415-supplement-Supplemental_Information.pdf]

Molecular Cell, Volume *83*

Supplemental information

Modular antibodies reveal DNA damage-induced mono-ADP-ribosylation as a second wave of PARP1 signaling

Edoardo José Longarini, Helen Dauben, Carolina Locatelli, Anne R. Wondisford, Rebecca Smith, Charlotte Muench, Andreas Kolvenbach, Michelle Lee Lynskey, Alexis Pope, Juan José Bonfiglio, Eva Pinto Jurado, Roberta Fajka-Boja, Thomas Colby, Marion Schuller, Ivan Ahel, Gyula Timinszky, Roderick J. O'Sullivan, Sébastien Huet, and Ivan Matic

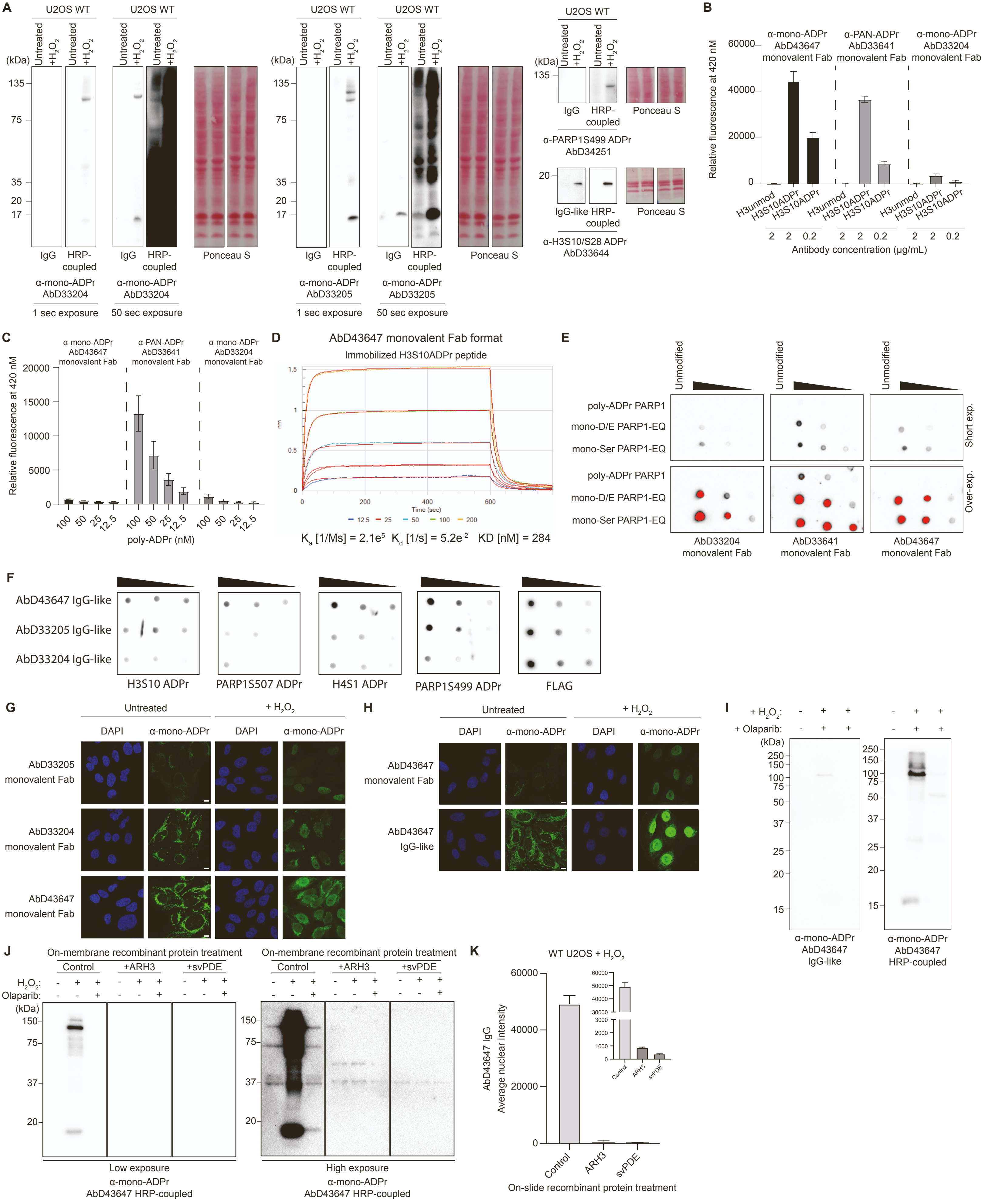

A
U2OS WT
Untreated
+H2O2
(kDa)
135
75
35
20
17
IgG
HRP-coupled
Ponceau S
α-mono-ADPr AbD33204
1 sec exposure
50 sec exposure
α-mono-ADPr AbD33205
α-PARP1S499 ADPr AbD34251
IgG-like HRP-coupled
α-H3S10/S28 ADPr AbD33644
B
α-mono-ADPr AbD43647 monovalent Fab
α-PAN-ADPr AbD33641 monovalent Fab
α-mono-ADPr AbD33204 monovalent Fab
Relative fluorescence at 420 nM
H3unmod
H3S10ADPr
Antibody concentration (µg/mL)
C
poly-ADPr (nM)
D
AbD43647 monovalent Fab format
Immobilized H3S10ADPr peptide
Time (sec)
Ka [1/Ms] = 2.1e5 Kd [1/s] = 5.2e-2 KD [nM] = 284
E
Unmodified
poly-ADPr PARP1
mono-D/E PARP1-EQ
mono-Ser PARP1-EQ
Short exp.
Over-exp.
AbD33204 monovalent Fab
AbD33641 monovalent Fab
AbD43647 monovalent Fab
F
AbD43647 IgG-like
AbD33205 IgG-like
AbD33204 IgG-like
H3S10 ADPr
PARP1S507 ADPr
H4S1 ADPr
PARP1S499 ADPr
FLAG
G
Untreated
+ H2O2
DAPI
α-mono-ADPr
AbD33205 monovalent Fab
AbD33204 monovalent Fab
AbD43647 monovalent Fab
H
AbD43647 monovalent Fab
AbD43647 IgG-like
I
+ H2O2:
+ Olaparib:
α-mono-ADPr AbD43647 IgG-like
α-mono-ADPr AbD43647 HRP-coupled
J
On-membrane recombinant protein treatment
Control
+ARH3
+svPDE
Low exposure
High exposure
α-mono-ADPr AbD43647 HRP-coupled
K
WT U2OS + H2O2
AbD43647 IgG Average nuclear intensity
Control
ARH3
svPDE
On-slide recombinant protein treatment

Figure S1. SpyTag-based modular antibodies enable sensitive and versatile detection of mono-ADPr, related to Figure 1

(**A**) Immunoblotting images showing comparison between IgG and HRP-coupled format for the indicated antibodies (see also **Figure 1A and S1I**). WT U2OS cells were either left untreated or treated with 2 mM H_2O_2 for 10 min and lysed in 4% SDS lysis buffer. (**B**) ELISA analysis of antibody sensitivity. The antibodies AbD33641, AbD33204, or the derived affinity maturated AbD43647 were incubated at the indicated concentrations with immobilized biotinylated peptides (H3S10unmodified or H3S10ADPr, 61 nM). Graphs showing mean ± SEM of n=2 technical replicates. (**C**) ELISA analysis of antibody specificity. The antibodies AbD33641, AbD33204, or the derived affinity maturated AbD43647 were incubated at 0.2 µg/ml with biotinylated free-poly-ADP-ribose at the indicated concentrations. Graphs showing mean ± SEM of n=2 technical replicates. (**D**) Affinity measurements of AbD43647, in monovalent Fab format. H3(1-21)S10ADPr was used as the immobilized antigen. K_a [1/Ms] = 2.1e^5 K_d [1/s] = 5.2e^{-2} KD [nM] = 284. (**E**) Dot blot analysis of *in vitro* automodified (i) PARP1 WT for poly-ADPr, (ii) PARP1-E988Q (PARP1-EQ) mutant in the absence of HPF1 for mono-ADPr on Asp/Glu, and (iii) PARP1-E988Q mutant in the presence of HPF1 WT for mono-ADPr on Ser. Bottom panels (high-exposed immunoblots of the top panels) show poly-ADPr of PARP1 WT. (**F**) Dot blot analysis of AbD43647, AbD33205, and AbD33204 antibodies blotted at 0.5, 0.25, 0.125 µg and incubated with the indicated biotinylated ADPribosylated peptides. Peptide binding was detected using anti-Streptavidin HRP antibody. (**G**) IF analysis of WT U2OS cells, untreated or treated with 2 mM H_2O_2 for 10 min, PFA fixed and stained with the indicated antibodies. (**H**) As in (**G**), IF analysis with the indicated antibodies. Detection of mono-ADPr in IF is dramatically improved upon conversion of Fab to bivalent IgG-like antibody. (**I**) Immunoblotting images showing comparison between IgG-like and HRP-coupled for the affinity maturated AbD43647. WT U2OS cells were treated with either 2 mM H_2O_2 for 10 min or 1 µM Olaparib for 30 min followed by 2 mM H_2O_2 for 10 min, and analyzed by immunoblotting with the indicated antibodies. Detection of mono-ADPr in WB is dramatically improved upon conversion of IgG-like to directly-coupled HRP antibody format. (**J**) Immunoblotting images showing that AbD43647 displays minimal signal after Serine-ADPr is removed by on-membrane recombinant ARH3 treatment or all forms of ADPr are hydrolyzed to phosphoribose by on-membrane svPDE treatment. (**K**) IF analysis of WT U2OS cells treated with 2 mM H_2O_2 for 10 min then fixed with Methanol and treated with the corresponding recombinant proteins. As in (**J**), AbD43647 display minimal nuclear signal after on-slide recombinant ARH3 and svPDE treatment.

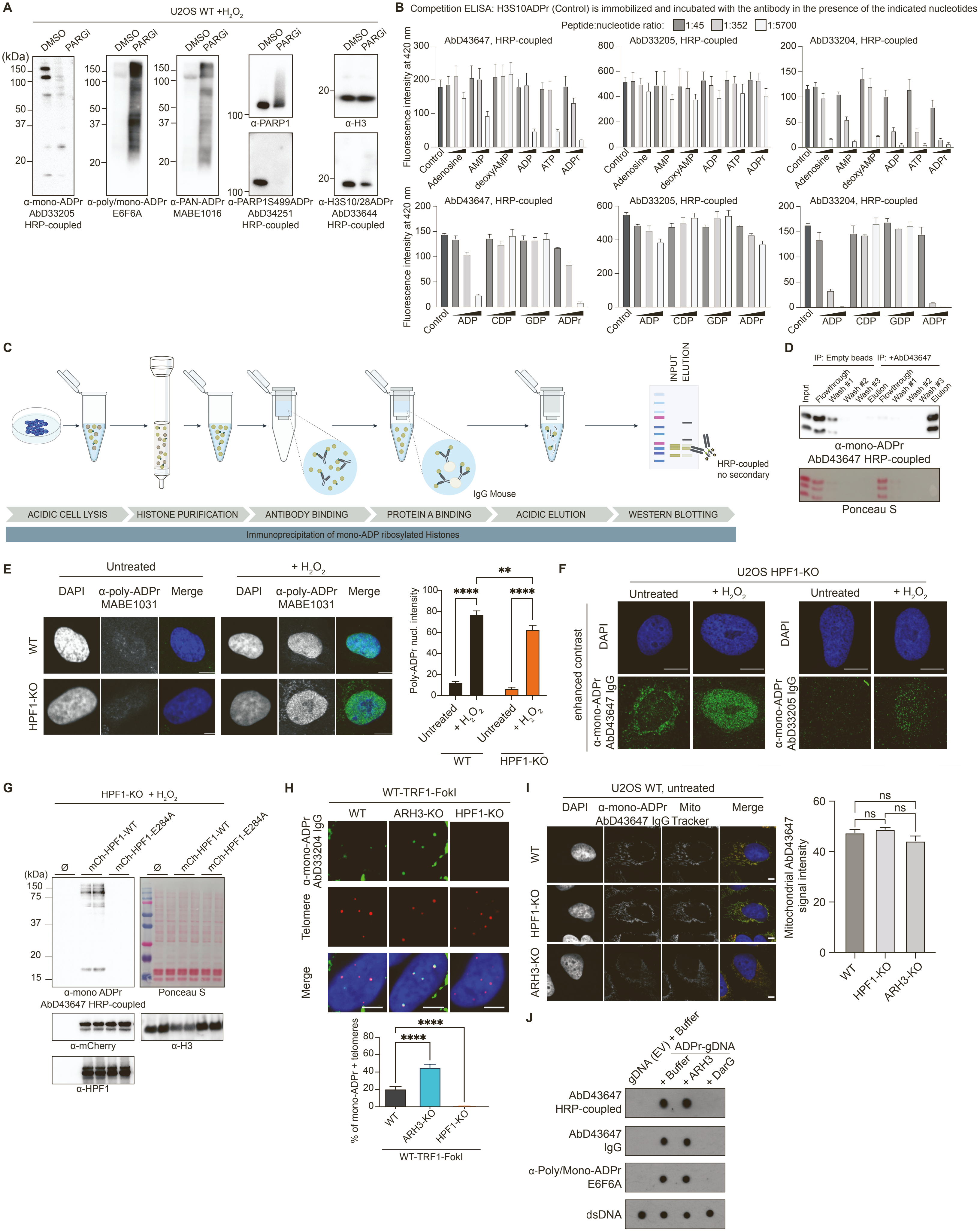
A
U2OS WT +H2O2
DMSO
PARGi
(kDa)
150
100
50
37
20
α-mono-ADPr
AbD33205
HRP-coupled
α-poly/mono-ADPr
E6F6A
α-PAN-ADPr
MABE1016
α-PARP1
α-PARP1S499ADPr
AbD34251
HRP-coupled
α-H3
α-H3S10/28ADPr
AbD33644
HRP-coupled
B
Competition ELISA: H3S10ADPr (Control) is immobilized and incubated with the antibody in the presence of the indicated nucleotides
Peptide:nucleotide ratio: 1:45 1:352 1:5700
AbD43647, HRP-coupled
AbD33205, HRP-coupled
AbD33204, HRP-coupled
Fluorescence intensity at 420 nm
Control
Adenosine
AMP
deoxyAMP
ADP
ATP
ADPr
CDP
GDP
C
ACIDIC CELL LYSIS
HISTONE PURIFICATION
ANTIBODY BINDING
PROTEIN A BINDING
ACIDIC ELUTION
WESTERN BLOTTING
Immunoprecipitation of mono-ADP ribosylated Histones
IgG Mouse
INPUT
ELUTION
HRP-coupled
no secondary
D
IP: Empty beads
IP: +AbD43647
Input
Flowthrough
Wash #1
Wash #2
Wash #3
Elution
α-mono-ADPr
AbD43647 HRP-coupled
Ponceau S
E
Untreated
+ H2O2
DAPI
α-poly-ADPr
MABE1031
Merge
WT
HPF1-KO
Poly-ADPr nucl. intensity
**
****
F
U2OS HPF1-KO
enhanced contrast
α-mono-ADPr
AbD43647 IgG
AbD33205 IgG
G
HPF1-KO + H2O2
Ø
mCh-HPF1-WT
mCh-HPF1-E284A
(kDa)
150
75
37
20
15
α-mono ADPr
AbD43647 HRP-coupled
Ponceau S
α-mCherry
α-H3
α-HPF1
H
WT-TRF1-FokI
WT
ARH3-KO
HPF1-KO
α-mono-ADPr
AbD33204 IgG
Telomere
Merge
% of mono-ADPr + telomeres
****
I
U2OS WT, untreated
DAPI
α-mono-ADPr
AbD43647 IgG
Mito
Tracker
Merge
Mitochondrial AbD43647
signal intensity
ns
J
gDNA (EV) + Buffer
ADPr-gDNA
+ Buffer
+ ARH3
+ DarG
AbD43647
HRP-coupled
AbD43647
IgG
α-Poly/Mono-ADPr
E6F6A
dsDNA

Figure S2. Dependence of mono-ADPr on HPF1 and validation of live cell imaging probes, related to Figure 1

(**A**) Immunoblotting images showing the mono-ADPr specificity of AbD33205, AbD34251, and AbD33644. WT U2OS cells were treated with either DMSO or 1 µM PARGi for 20 min followed by 2 mM H_2O_2 for 10min and lysed in 4% SDS lysis buffer. Related to **Figure 1C**. (**B**) Competition ELISA analysis of antibody epitopes. The antibodies AbD33205, AbD33204, or the derived affinity maturated AbD43647 were incubated with increasing concentrations of ADPr-related small molecules: 0 (control), 2.75 µM, 21.5 µM, 344 µM then incubated with immobilized H3S10ADPr peptide (61 nM). The signal intensity represents the amount of leftover antibody bound to the peptide. AbD33205 is not significantly affected by the nucleotides under any of the conditions tested, reflecting the lack of reactivity towards free-ADPr and indicating a strong preference for peptide-bound ADPr. Conversely, AbD33204 and to a lesser extent AbD43647 can cross-react with free-ADPr. Binding of AbD43647 to ADPr requires adenine and a free 2'-hydroxyl group on the adenine-proximal ribose. Binding is further strengthened by presence of 2 phosphate groups and to a lesser extent adenine-distal ribose. Graphs showing mean ± SEM (n=3, technical replicates). (**C**) Schematic illustration showing the immunoprecipitation workflow used to detect mono-ADPr on purified histones coupled with sensitive immunoblot detection. Related to **Figure 1D.** (**D**) Immunoblotting images showing selective enrichment of ADPribosylated histones upon pulldown with AbD43647. Related to **Figure 1D.** (**E**) IF analysis of WT or HPF1-KO U2OS cells, untreated or treated with 2 mM H_2O_2 for 10 min, methanol fixed and stained for poly-ADPr with the MABE1031 antibody. Related to **Figure 1E**. Data is mean ± SEM from a representative of n = 3 biological replicates. **** $P < 0.0001$; ** $P < 0.01$ (two-way ANOVA). (**F**) Enhanced contrast of the images from **Figure 1E**, showing mono-ADPr levels detected by AbD43647 in HPF1-KO U2OS cells upon treatment with 2 mM H_2O_2 for 10 min. (**G**) HPF1-KO U2OS cells were mock transfected or transfected with mCherry-HPF1-WT or mCherry-HPF1-E284A, treated with 1 mM H_2O_2 for 20 min, lysed and subjected to immunoblotting with the indicated antibodies. The two side-by-side lanes represent biological replicates. (**H**) Top: IF images of WT, ARH3-KO, and HPF1-KO U2OS cells transfected with FLAG-tagged WT-TRF1-FokI - to induce telomere-localized DNA damage - or DA-TRF1-FokI (D450A, catalytic dead mutant which does not induce DNA damage), fixed with methanol/acetone and stained with the indicated antibodies. Bottom: corresponding quantification of of mono-ADPr positive telomeres (%). Results are mean ± SEM. n = 3 biological replicates, 50 cells. **** $P < 0.0001$ (one-way ANOVA). Related to **Figure 1F.** Scale bars: 5 µm. (**I**) IF images (left) of WT, ARH3-KO, and HPF1-KO U2OS cells treated with MitoTracker red to stain mitochondria (500 nM for 15 min), fixed with PFA and stained with the indicated antibodies. Scale bars: 5 µm. Corresponding quantification (right) of AbD43647 signal overlapping with mitochondria staining. Data is mean ± SEM from a representative of n = 3 biological replicates. ns, not significant (unpaired Student's t test). Related to **Figure 1G**. (**J**) Dot blot of genomic DNA (gDNA) ADPr treated with 1 µM recombinant ARH3 or DarG for 30 min at 37 °C before staining with the indicated antibodies. Related to **Figure 1H**.

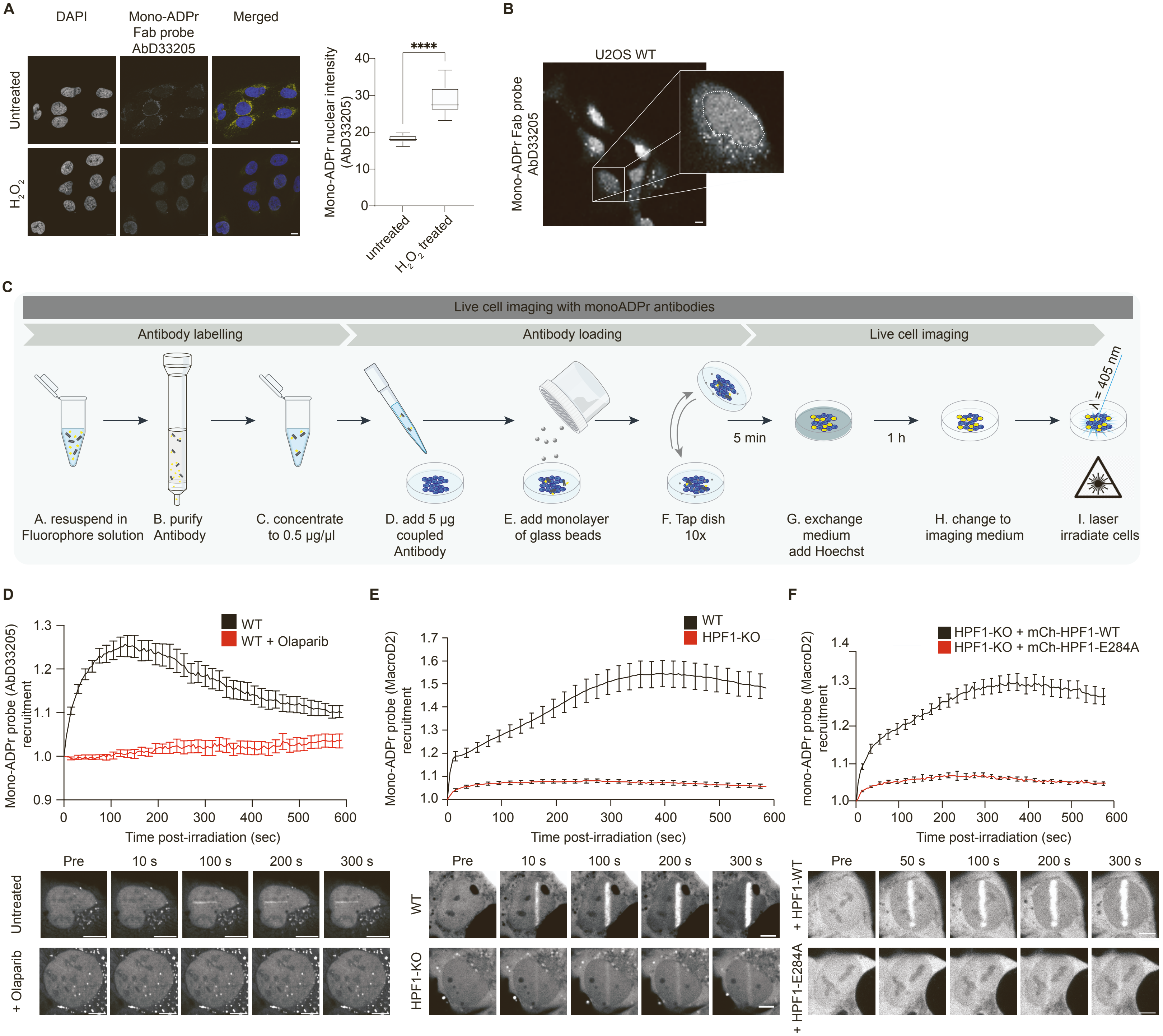
A
DAPI
Mono-ADPr Fab probe AbD33205
Merged
Untreated
H_2O_2
Mono-ADPr nuclear intensity (AbD33205)
****
untreated
H_2O_2 treated
B
U2OS WT
Mono-ADPr Fab probe AbD33205
C
Live cell imaging with monoADPr antibodies
Antibody labelling
Antibody loading
Live cell imaging
λ = 405 nm
5 min
1 h
A. resuspend in Fluorophore solution
B. purify Antibody
C. concentrate to 0.5 µg/µl
D. add 5 µg coupled Antibody
E. add monolayer of glass beads
F. Tap dish 10x
G. exchange medium add Hoechst
H. change to imaging medium
I. laser irradiate cells
D
WT
WT + Olaparib
Mono-ADPr probe (AbD33205) recruitment
Time post-irradiation (sec)
Pre
10 s
100 s
200 s
300 s
Untreated
+ Olaparib
E
WT
HPF1-KO
Mono-ADPr probe (MacroD2) recruitment
Time post-irradiation (sec)
Pre
10 s
100 s
200 s
300 s
WT
HPF1-KO
F
HPF1-KO + mCh-HPF1-WT
HPF1-KO + mCh-HPF1-E284A
mono-ADPr probe (MacroD2) recruitment
Time post-irradiation (sec)
Pre
50 s
100 s
200 s
300 s
+ HPF1-WT
+ HPF1-E284A

Figure S3. Fluorescence-based sensors reveal DNA damage-induced serine mono-ADPr as second wave of PARP1 signaling, related to Figure 2

(**A**) IF analysis of WT U2OS cells treated or not with 2 mM H_2O_2 for 10 min, fixed and stained with AbD33205 coupled to dyLIGHT550, used as live cell mono-ADPr Fab probe (see also **Figure 1A and 2A**). Data is mean ± SEM from a representative of n = 3 biological replicates. **** $P < 0.0001$ (unpaired Student's t test). (**B**) Representative confocal image of AbD33205 coupled to dyLIGHT550 (mono-ADPr Fab probe) showing nuclear localization of the antibody loaded into live cells using the bead-loading method. (**C**) Schematic illustration showing the antibody labeling, bead-loading and laser microirradiation workflow used to detect mono-ADPr in live cells. Briefly, an antibody solution is added to the cells and immediately covered by a monolayer of beads. The antibodies are not attached to the glass beads and the beads do not enter the nuclei, but are simply used to induce a temporary mechanical disruption to the cell membrane, allowing macromolecules, including antibodies, to enter the cells. After incubation, the beads are washed off and the medium replaced by live cell imaging solution. See also the "Antibody labelling", "Antibody loading", and "Protein recruitment kinetics at sites of laser irradiation" sections of the STAR Methods for details. (**D**) Top: recruitment kinetics of mono-ADPr Fab probe (AbD33205) in WT U2OS cells untreated (black) or treated with 30 μM Olaparib immediately prior to irradiation (red). The mean ± SEM from 10 cells from a representative of 3 independent experiments is shown. Bottom: representative confocal images. Scale bars: 10 μm. (**E**) Top: recruitment kinetics of mono-ADPr probe (GFP-tagged macrodomain of macroD2) to sites of DNA damage induced by laser irradiation, in WT and HPF1-KO U2OS cells. The mean ± SEM from 10–12 cells from a representative of 3 independent experiments is shown. Bottom: representative confocal images. Scale bar: 5 μm. (**F**) Top: recruitment kinetics of mono-ADPr probe (GFP-tagged macrodomain of macroD2) to sites of DNA damage induced by laser irradiation in HPF1-KO U2OS cells expressing mCherry-tagged HPF1-WT or HPF1-E284A. The mean ± SEM from 13–16 cells from a representative of 3 independent experiments is shown. Bottom: representative confocal images. Scale bar: 5 μm.

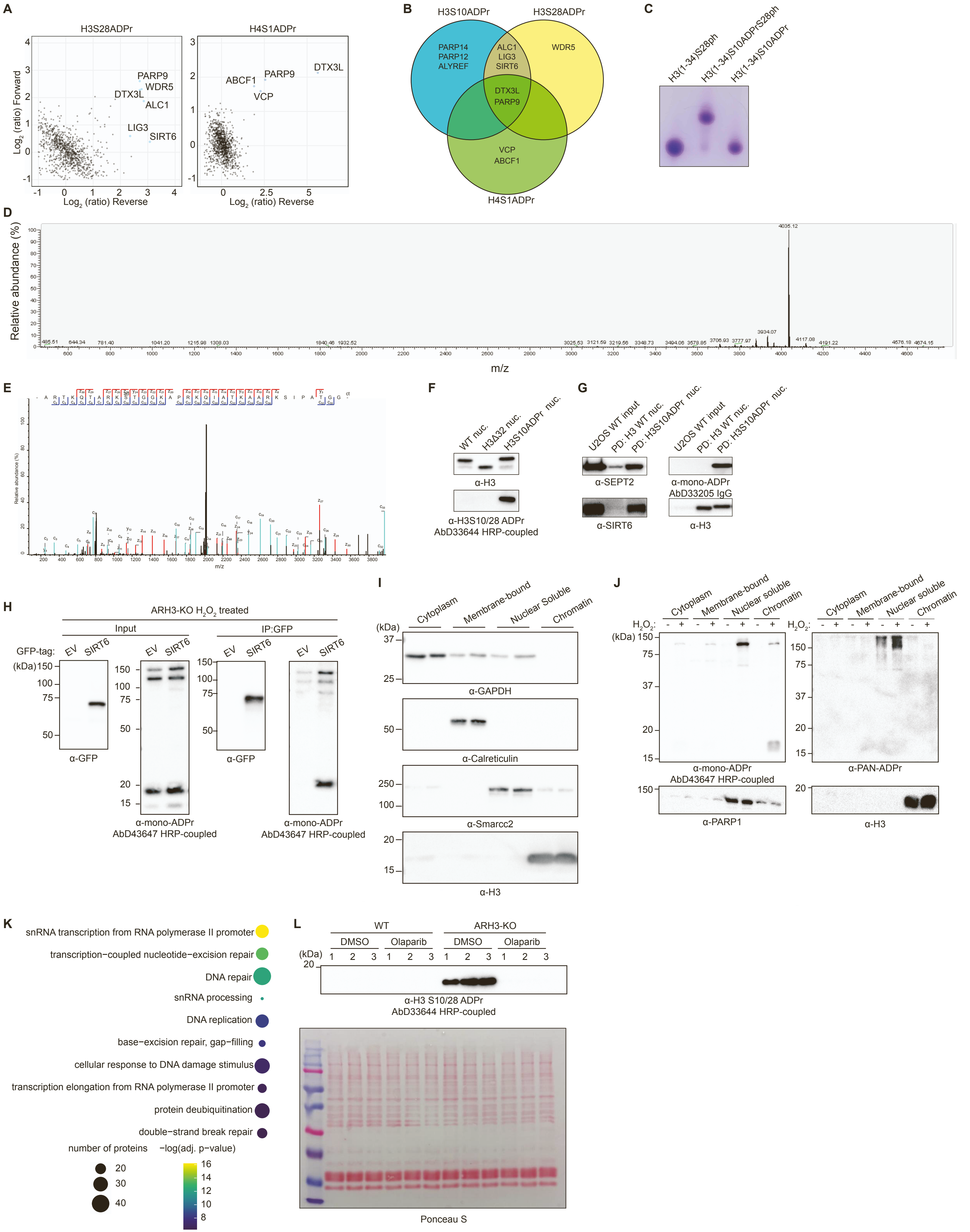
A
H3S28ADPr
H4S1ADPr
Log2 (ratio) Forward
Log2 (ratio) Reverse
PARP9
WDR5
DTX3L
ALC1
LIG3
SIRT6
ABCF1
VCP
B
H3S10ADPr
H3S28ADPr
H4S1ADPr
PARP14
PARP12
ALYREF
ALC1
LIG3
SIRT6
WDR5
DTX3L
PARP9
VCP
ABCF1
C
H3(1-34)S28ph
H3(1-34)S10ADPrS28ph
H3(1-34)S10ADPr
D
Relative abundance (%)
m/z
4035.12
3934.07
4117.08
E
Relative abundance (%)
m/z
F
WT nuc.
H3Δ32 nuc.
H3S10ADPr nuc.
α-H3
α-H3S10/28 ADPr
AbD33644 HRP-coupled
G
U2OS WT input
PD: H3 WT nuc.
PD: H3S10ADPr nuc.
α-SEPT2
α-SIRT6
α-mono-ADPr
AbD33205 IgG
α-H3
H
ARH3-KO H2O2 treated
Input
IP:GFP
GFP-tag:
EV
SIRT6
(kDa)
α-GFP
α-mono-ADPr
AbD43647 HRP-coupled
I
(kDa)
Cytoplasm
Membrane-bound
Nuclear Soluble
Chromatin
α-GAPDH
α-Calreticulin
α-Smarcc2
α-H3
J
Cytoplasm
Membrane-bound
Nuclear soluble
Chromatin
H2O2:
(kDa)
α-mono-ADPr
AbD43647 HRP-coupled
α-PAN-ADPr
α-PARP1
α-H3
K
snRNA transcription from RNA polymerase II promoter
transcription−coupled nucleotide−excision repair
DNA repair
snRNA processing
DNA replication
base−excision repair, gap−filling
cellular response to DNA damage stimulus
transcription elongation from RNA polymerase II promoter
protein deubiquitination
double−strand break repair
number of proteins
−log(adj. p−value)
L
WT
ARH3-KO
DMSO
Olaparib
(kDa)
α-H3 S10/28 ADPr
AbD33644 HRP-coupled
Ponceau S

Figure S4. Identification of mono-ADPr readers by chromatin proteomics, related to Figure 4

(**A**) Scatterplot showing proteins enriched by H3S28ADPr (left) and H4S1ADPr (right) peptides, compared to the unmodified peptide. Enriched proteins are shown in light blue. (Shown are n=2 forward and reverse samples as representative from 3 independent experiments). (**B**) Venn diagram illustrating the overlap between the H3S10ADPr, H3S28ADPr, H4S1ADPr peptide pulldown datasets, related to **Figure 4B and S4A**. (**C**) Inverted Polarity-TBE gel showing the steps of the phospho-guided ADPr reaction: (i) synthetic peptide precursor with phosphate as protecting group H3(1-34)S28ph; (ii) phosphopeptide ADP-ribosylated on the desired serine H3(1-34)S10ADPrS28ph; and (iii) site-specifically ADP-ribosylated peptide H3(1-34)S10ADPr. (**D**) Deconvoluted chromatogram showing purity of the H3(1-34)S10ADPr peptide. Mass = 4035.12. (**E**) ETD spectrum of H3(1-34)S10ADPr used for nucleosome generation showing site-specific ADPribosylation (ad) at the intended site. (**F**) WT unmodified nucleosomes, truncated nucleosomes used as input for native chemical ligation, and produced H3S10ADPr nucleosomes were probed with the indicated antibodies. (**G**) Immunoblot analysis of nucleosome pulldown showing enrichment of SEPT2 and SIRT6 to H3S10ADPr nucleosomes. PD = pulldown. Related to **Figure 4D.** (**H**) Immunoblotting images of ARH3-KO U2OS cells transfected with GFP- SIRT6, treated with 2 mM H_2O_2 for 30 min and subjected to immunoprecipitation using an anti-GFP antibody. Bound proteins were analyzed by immunoblotting using the indicated antibodies. (**I**) WT U2OS cells were subjected to subcellular fractionation, the resulting fractions were resolved by SDS-PAGE and fractionation efficiency was checked using GAPDH (Mainly cytosolic), Calreticulin (Membrane-bound), Smarcc2 (Nuclear Soluble), and H3 (Chromatin Bound). (**J**) WT U2OS cells were untreated or treated with 1 mM H_2O_2 for 20 min and subjected to subcellular fractionation. The samples were immunoblotted with the indicated antibodies. ADPr PARP1 is mainly nuclear soluble and ADPr histones mainly chromatin-bound, confirming previous reports (Prokhorova et al, mol cell). (**K**) Gene ontology (GO) analysis of the proteins with positive $\log_2$ fold change (enriched in H_2O_2-treated cells) from **Figure 4F**. Shown are the significant Biological Processes terms. (**L**) Ponceau-S loading control and corresponding mono-ADPr immunoblots of the chromatin extract samples used in the proteomics experiment in **Figure 4H-J**.

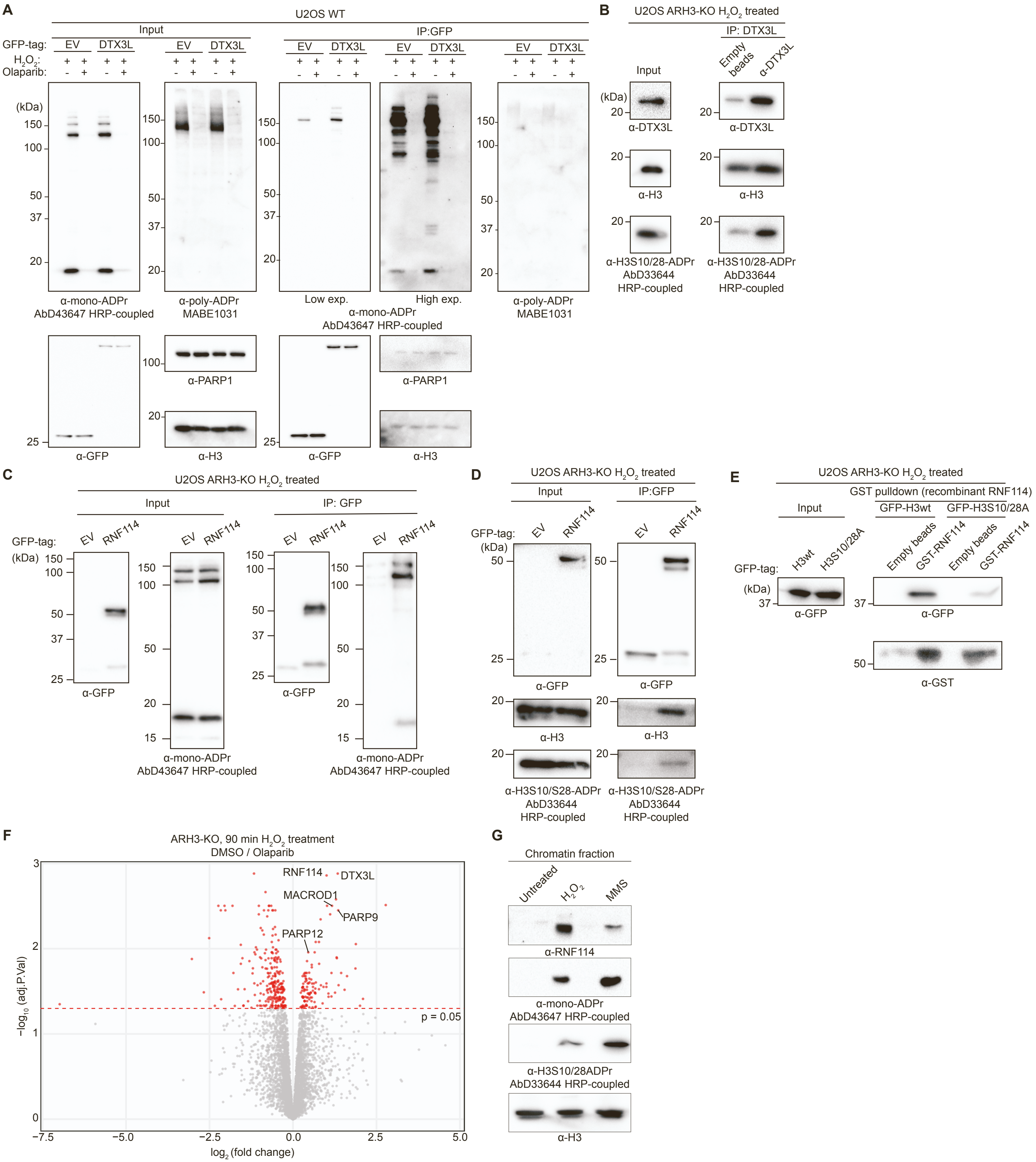
A
U2OS WT
Input
IP:GFP
GFP-tag: EV DTX3L
H_2O_2:
Olaparib:
(kDa)
α-mono-ADPr AbD43647 HRP-coupled
α-poly-ADPr MABE1031
Low exp.
High exp.
α-PARP1
α-GFP
α-H3
B
U2OS ARH3-KO H_2O_2 treated
IP: DTX3L
Input
Empty beads
α-DTX3L
α-H3
α-H3S10/28-ADPr AbD33644 HRP-coupled
C
U2OS ARH3-KO H_2O_2 treated
IP: GFP
RNF114
D
IP:GFP
α-H3S10/S28-ADPr AbD33644 HRP-coupled
E
GST pulldown (recombinant RNF114)
GFP-H3wt
GFP-H3S10/28A
H3wt
H3S10/28A
GST-RNF114
α-GST
F
ARH3-KO, 90 min H_2O_2 treatment
DMSO / Olaparib
RNF114
DTX3L
MACROD1
PARP9
PARP12
p = 0.05
$-\log_{10}$ (adj.P.Val)
$\log_2$ (fold change)
G
Chromatin fraction
Untreated
H_2O_2
MMS
α-RNF114
α-H3S10/28ADPr AbD33644 HRP-coupled

Figure S5. Validation of mono-ADPr readers identified by chromatin proteomics, related to Figure 4

(**A**) Immunoblotting images of WT U2OS cells, transfected with GFP-Empty Vector (GFP-EV) or GFP-DTX3L, treated with DMSO or 1 μM Olaparib for 30 min, then treated with 2 mM H_2O_2 for 30 min. Cell lysates were incubated with magnetic beads conjugated to an anti-GFP antibody. Bound proteins were immunoblotted and stained with the indicated antibodies. (**B**) Immunoblotting images of WT U2OS cells treated with 2 mM H_2O_2 for 30 min. Cell lysates were incubated with magnetic protein A beads, either empty or coupled to anti-DTX3L antibody. Bound proteins were immunoblotted and stained with the indicated antibodies. (**C, D**) Immunoblotting images of ARH3-KO U2OS cells, transfected with GFP-Empty Vector (GFP-EV) or GFP-RNF114, treated with 2 mM H_2O_2 for 30 min. Cell lysates were incubated with magnetic beads conjugated to an anti-GFP antibody. Bound proteins were immunoblotted and stained with the indicated antibodies. (**E**) Immunoblotting images of ARH3-KO U2OS cells transfected with GFP-tagged H3-WT or H3-S10/28A mutant, treated with 2 mM H_2O_2 for 30 min. Cell lysates were incubated with either empty GST agarose beads or beads coupled to GST-tagged RNF114. Bound proteins were immunoblotted and stained with the indicated antibodies. (**F**) ARH3-KO U2OS cells were treated with DMSO or 1 μM Olaparib for 48 h, then either left untreated or treated with 1 mM H_2O_2 for 90 min. Cells were then subjected to subcellular fractionation and the resulting chromatin bound fraction was analyzed by DIA LC-MS/MS. Volcano plot showing the log2-fold change of detected proteins. the red dotted line represents significance with P-value=0.05 ($-\log_{10}$(adj. P-value)>1.3) cut-off, Significant proteins are indicated in red. (n = 4, biological replicates). (**G**) U2OS WT cells were either left untreated, treated with 1 mM H_2O_2 for 20 min, or treated with 4 mM MMS for 30 min and subjected to subcellular fractionation. The chromatin bound fraction was analyzed by immunoblotting using the indicated antibodies.

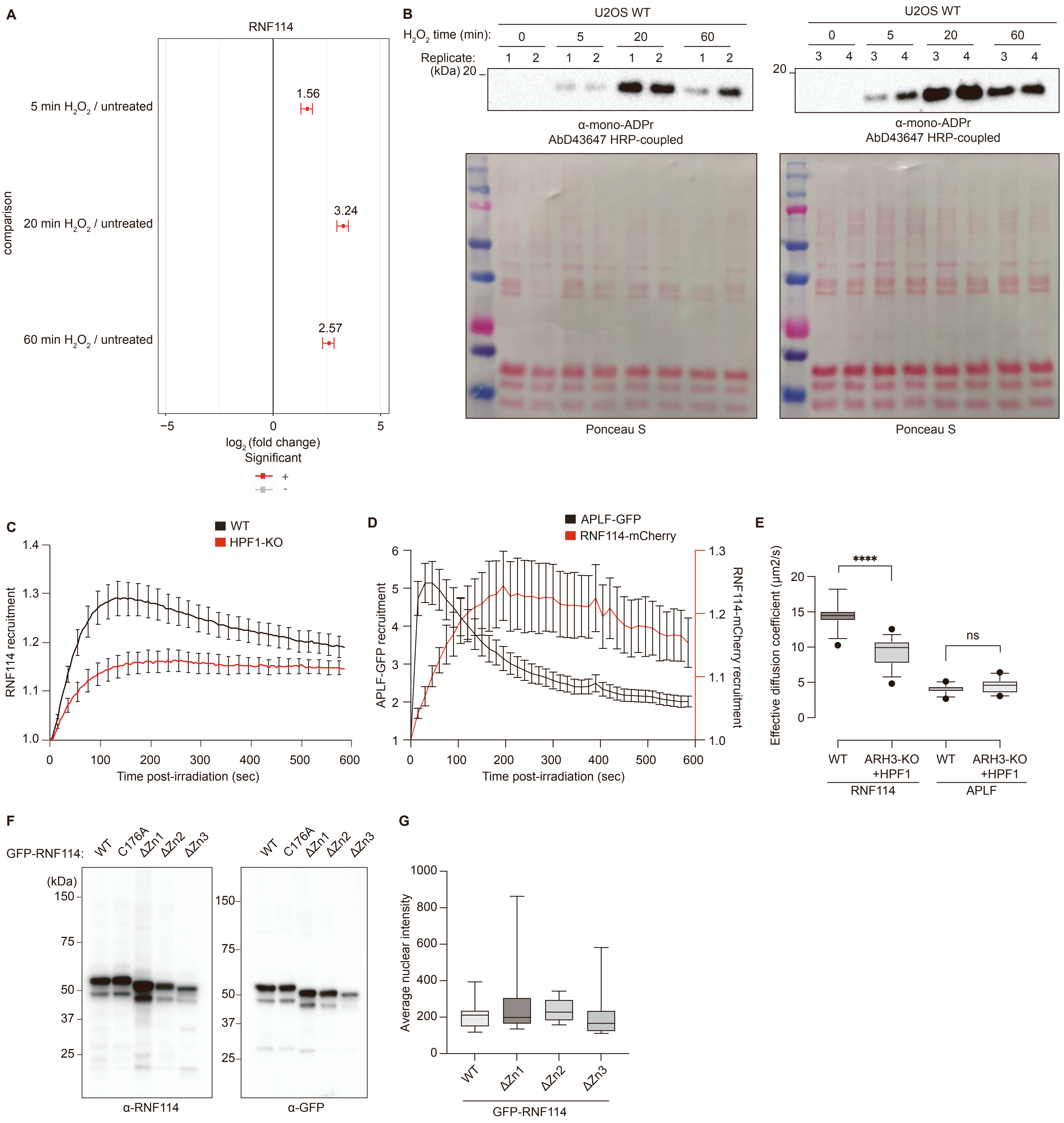
A
RNF114
5 min H2O2 / untreated
20 min H2O2 / untreated
60 min H2O2 / untreated
comparison
1.56
3.24
2.57
−5
0
5
log2 (fold change)
Significant
+
-
B
U2OS WT
H2O2 time (min):
0
5
20
60
Replicate:
1
2
3
4
(kDa) 20
α-mono-ADPr
AbD43647 HRP-coupled
Ponceau S
C
WT
HPF1-KO
RNF114 recruitment
1.4
1.3
1.2
1.1
1.0
0
100
200
300
400
500
600
Time post-irradiation (sec)
D
APLF-GFP
RNF114-mCherry
APLF-GFP recruitment
RNF114-mCherry recruitment
Time post-irradiation (sec)
E
Effective diffusion coefficient (µm2/s)
****
ns
WT
ARH3-KO +HPF1
RNF114
APLF
F
GFP-RNF114:
WT
C176A
ΔZn1
ΔZn2
ΔZn3
(kDa)
150
75
50
37
25
α-RNF114
α-GFP
G
Average nuclear intensity
1000
800
600
400
200
0
GFP-RNF114

Figure S6. Chromatin mono-ADPr functions as a recruitment signal for RNF114, related to Figure 5 and 6

(**A**) Log_2 fold change plots for RNF114 corresponding to **Figure 5A**. (**B**) Ponceau S loading control and corresponding mono-ADPr immunoblots of the chromatin extract samples used in the proteomics experiment in **Figure 5A**. (**C**) Recruitment kinetics of GFP-RNF114 in WT (black) or HPF1-KO (red) U2OS cells. The mean ± SEM of 16 cells from a representative of 3 independent experiments is shown. Related to **Figure 5C**. (**D**) Recruitment kinetics of co-expressed GFP-tagged APLF (black) and mCherry-tagged RNF114 (red) in WT U2OS cells. The mean ± SEM from 16 cells from a representative experiment is shown. (**E**) Effective diffusion coefficient measured by FCS for RNF114 (left) and APLF (right). WT or ARH3-KO U2OS cells were transfected with GFP-tagged RNF114 or APLF alone or together with mCherry-tagged HPF1. ****, P-value < 0.0001; ns, not significant (unpaired Student's t test assuming unequal variances). n=3 independent replicates. (**F**) Immunoblotting analysis of the GFP-tagged RNF114-WT and corresponding mutants expressed in U2OS WT cells, see **Figure 6C**. (**G**) Corresponding relative average nuclear intensity for the constructs used in **Figure 6D**, showing that despite the lower expression level of RNF114-ΔZn3 (**Figure S6F**) for the live cell experiment we selected cells with similar expression levels across all constructs.

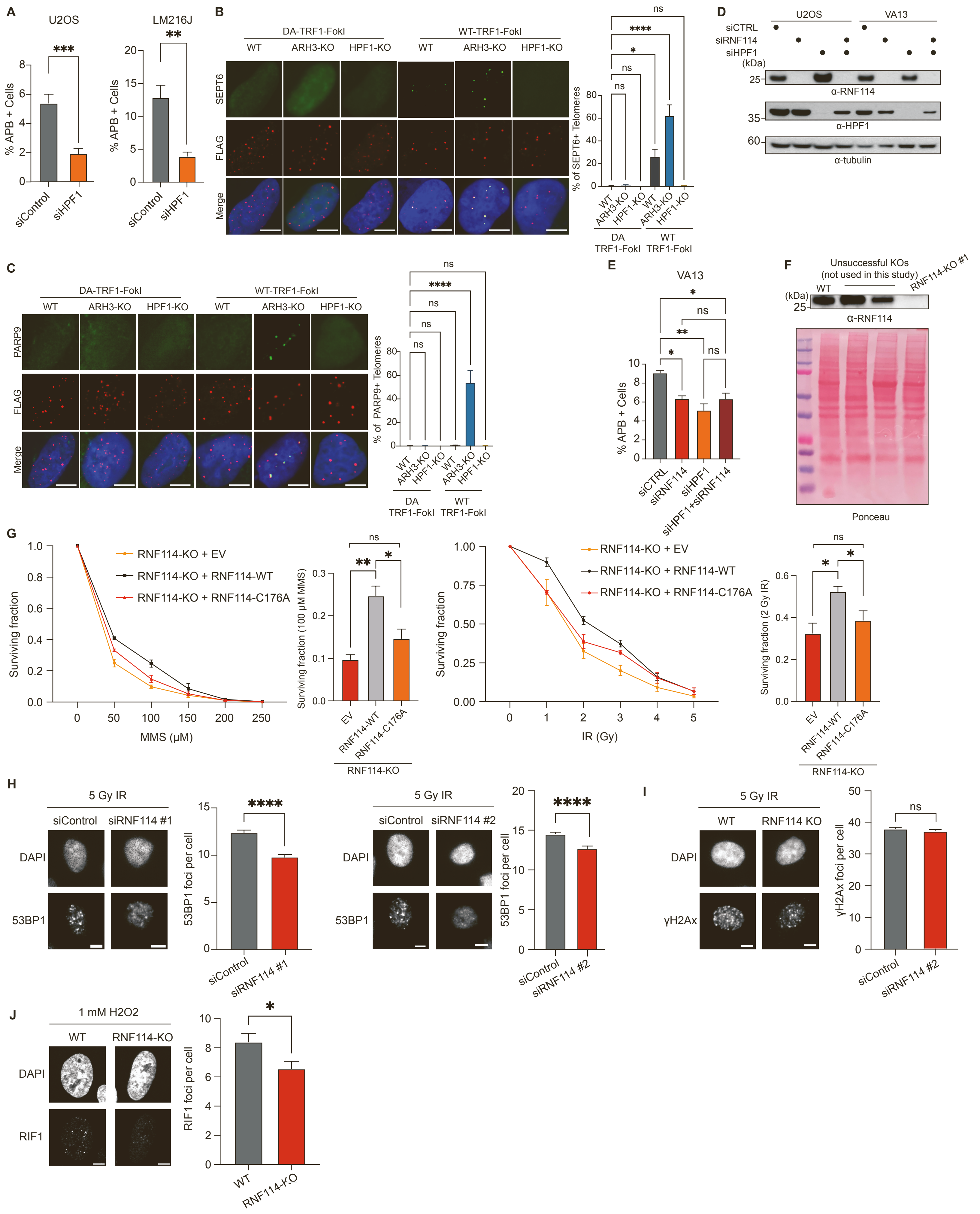
A
U2OS
LM216J
% APB + Cells
siControl
siHPF1
B
DA-TRF1-FokI
WT-TRF1-FokI
WT
ARH3-KO
HPF1-KO
SEPT6
FLAG
Merge
% of SEPT6+ Telomeres
DA TRF1-FokI
WT TRF1-FokI
ns
D
U2OS
VA13
siCTRL
siRNF114
siHPF1
(kDa)
α-RNF114
α-HPF1
α-tubulin
C
PARP9
% of PARP9+ Telomeres
E
VA13
% APB + Cells
siCTRL
siRNF114
siHPF1
siHPF1+siRNF114
F
Unsuccessful KOs
(not used in this study)
RNF114-KO #1
α-RNF114
Ponceau
G
RNF114-KO + EV
RNF114-KO + RNF114-WT
RNF114-KO + RNF114-C176A
Surviving fraction
MMS (μM)
Surviving fraction (100 μM MMS)
EV
RNF114-WT
RNF114-C176A
RNF114-KO
IR (Gy)
Surviving fraction (2 Gy IR)
H
5 Gy IR
siControl
siRNF114 #1
siRNF114 #2
DAPI
53BP1
53BP1 foci per cell
I
WT
RNF114 KO
γH2Ax
γH2Ax foci per cell
J
1 mM H2O2
RNF114-KO
RIF1
RIF1 foci per cell

Figure S7. RNF114 modulates the alternative lengthening of telomeres pathway and the DNA damage response, related to Figure 7

(**A**) Representative IF images (left) and quantification (right) of ALT-associated PML bodies (APBs) in U2OS and LM216J cells transfected with siControl or siRNA for HPF1 (siHPF1). ***, P-value < 0.001; **, P-value < 0.01. (unpaired Student's t test). (**B**) Left: Representative images of WT, ARH3-KO, or HPF1-KO U2OS cells co-transfected with FLAG-TRF1-FokI WT or FLAG-TRF1-FokI DA (D450A, catalytic dead mutant) and GFP-SEPT6, fixed and stained with the indicated antibodies. Right: quantification of SEPT6 positive telomeres (%). Data are mean ± SEM. (n = 3, biological replicates each 50 cells). ****, P-value < 0.0001; *, P-value < 0.05; ns, not significant (one-way ANOVA). Scale bars: 5 µm. (**C**) Left: Representative images of WT, ARH3-KO, or HPF1-KO U2OS cells co-transfected with FLAG-TRF1-FokI WT or FLAG-TRF1-FokI DA (D450A, catalytic dead mutant) and GFP-PARP9, fixed and stained with the indicated antibodies. Right: quantification of PARP9 positive telomeres (%). Data are mean ± SEM. (n = 3, biological replicates each 50 cells). ****, P-value < 0.0001; ns, not significant (one-way ANOVA). Scale bars: 5 µm. (**D**) Immunoblot images showing efficient siRNA of RNF114 (siRNF114) and HPF1 (siHPF1) in U2OS and VA13 cell lines. Related to **Figure 7C and S7E**. (**E**) Quantification of APBs in VA13 WT cells tranfected with siRNA for control (siControl), HPF1 (siHPF1), RNF114 (siRNF114), or HPF1 + siRNF114. Data are mean ± SEM. ** P-value < 0.01; * P-value < 0.05; ns, not significant. Related to **Figure 7C**. (**F**) Immunoblot images of WT and RNF114-KO U2OS cells. Ponceau was used as loading control. (**G**) Clonogenic cell survival assay of RNF114-KO U2OS cells stably complemented with either GFP-EV, GFP-RNF114-WT, or GFP-RNF114-C176A. DNA damage was induced by continuous MMS treatment (left) and IR (right) at the indicated doses. Bar plots represent corresponding mean ±SEM of n=3 independent replicates at the indicated MMS and IR doses. ** P-value < 0.01; * P-value < 0.05; ns, not significant (two-tailed Student's t test). (**H**) IF images and quantification of 53BP1 foci after 1 h 5 Gy IR treatment in WT HeLa cells transfected with siControl, siRNA #1 (left) or siRNA #2 (right) for RNF114. The mean ± SEM from 100 cells from a representative of 3 independent experiment is shown. **** P-value < 0.0001 (two-tailed Student's t test). Scale bars: 5 µm. (**I**) Representative IF images (left) and quantification (right) of γH2Ax foci after 5 Gy IR in HeLa WT cells transfected with siControl or siRNA #2 for RNF114. The mean ± SEM from 100 cells from a representative of 2 independent experiment is shown. ns, not significant (two-tailed Student's t test) Related to **Figure 7H**. (**J**) Representative IF images (left) and quantification (right) of RIF1 foci after 1 h 1 mM H_2O_2 treatment in WT or RNF114-KO U2OS cells. The mean ± SEM from 100 cells from a representative of 2 independent experiment is shown. * P-value < 0.05 (two-tailed Student's t test). Scale bars: 5 µm. Related to **Figure 7K**.
