# Supplementary material for: Modular antibodies reveal DNA damage-induced mono-ADP-ribosylation as a second wave of PARP1 signaling
Source: Mol Cell. Author manuscript; Available in PMC 2025 Apr 24. (PMC10205078; doi:10.1016/j.molcel.2023.03.027)
Supplement: Methods S1 [file NIHMS1899415-supplement-Methods_S1.pdf]

Methods S1

Detailed protocol for immunoblotting using the HRP-coupled SpyTag format

In immunoblotting, detection of primary antibodies is conventionally caried out using corresponding secondary antibodies labeled with horseradish peroxidase (HRP). However, as shown in **Figures S1A and S1I**, we observed that site-directed labelling with three copies of horseradish peroxidase (HRP) through SpyTag coupling increased the sensitivity dramatically compared to the standard combination of conventional IgG format and HRP-labeled secondary antibody. Although HRP labeling of primary antibodies can also be achieved by randomly conjugating HRP to the lysine residues of the antibody, this chemical labeling strategy is often laborious, time-consuming and expensive. Second, as the latter approach is not site-specific, it renders a fraction of the antibody unable to bind the corresponding antigen whenever its antigen-binding site is blocked by the chemical HRP conjugation. Third, each antibody carries a different number of HRPs at different locations and a new batch of chemically labeled antibody will probably have a different degree of HRP conjugation. In contrast, SpyTag-based HRP labeling avoid these drawbacks since this modular strategy is highly reproducible, site-specific with HRP conjugated away from the antigen binding site. The resulting antibodies have a fixed number of HRPs at defined positions and can be easily obtained either as final off-the-shell products, such as AbD43647pap (Bio-Rad, Catalog # TZA020P), or by simply mixing the SpyTagged Fab with BiCatcher2:HRP (Bio-Rad, Catalog # TZC002P) and incubating them at room temperature for one hour, as detailed in this protocol (https://www.bio-rad-antibodies.com/static/2021/custom/fab-antibody-coupling-to-bivalent-bicatchers.pdf).

An important feature of our HRP-coupled mono-ADPr antibodies is their very high sensitivity, which has two important advantages. First, for samples with abundant mono-ADPr, such as when HPF1/PARP1 is activated upon H_2O_2 treatment, these antibodies should be used at higher dilutions (see below for details) than when immunoblotting is performed using conventional IgG

primary/secondary pairs. This not only significantly lowers the costs of performing immunoblotting (low amounts of primary and no secondary antibody), but also avoids the risk of signal oversaturation that can occur as a result of the high sensitivity of this format. Second, for lower mono-ADPr levels it is possible and recommended to dramatically increase the concentration (up to 1-2 µg/ml and possibly even higher). Thus, to take full advantage of this format, we recommend considering a wide range of dilutions by taking into account both the affinity of the specific antibody used (for example AbD33205, AbD34251 and AbD33644 have lower affinity than AbD43647) and levels of mono-ADPr investigated. Please refer to our step-by-step guidelines for best practices with these antibodies:

- Membrane transfer can be performed with any standard protocol. In our experience, for detection of ADPr on low- and high-molecular weight proteins, we obtain best results with wet overnight transfer at 90 mA on PVDF membranes with 0.2 µm pore size. Semi-dry turbo transfer (10 min) may also be used and is particularly indicated for low-molecular weight ADP-ribosylated proteins (e.g. histones).
- Antibody incubation (in 5% BSA or 5% milk TBS-T with 0.05% tween) can be performed overnight at 4˚C or for 2h at room temperature and in our experience yield similar results.
    - If the antibody incubation is performed at 4˚C, it can be stored and reused several times. For medium-term storage >2-3 days, up to 2 weeks, we recommend storage at 4˚C with addition of a preservative such as ProClin (Sigma-Aldrich cat# 48914-U) at 1:1000 dilution. Sodium azide use it not recommended as it acts as HRP inhibitor. For long-term storage the antibody solution can be frozen and stored at -20˚C although repeated freeze-thaw cycles will decrease the performance of the antibody.
- For samples with high levels of mono-ADPr, such as upon DNA damage by H_2O_2 or MMS treatment, we recommend the following starting concentrations:
    - AbD43647pap 0.05 µg/ml
    - AbD43205pap 0.1 µg/ml

- AbD33644pap 0.2 µg/ml
- AbD34251pap 0.4 µg/ml

- After antibody incubation and extensive washes (x6 washes for 5-10 minutes in TBS-T), acquire the signal. If the obtained signal is faint or undetectable, the membrane may be reincubated with higher amounts of antibody, such as 1-2 µg/ml. The use of a high-sensitivity detection reagent, such as SuperSignal West Atto (Thermo Scientific, Cat# A38554) may also be used as in our experience yields good results.
