# Supplementary material for: Modular antibodies reveal DNA damage-induced mono-ADP-ribosylation as a second wave of PARP1 signaling
Source: Mol Cell. 2023 May 18;83(10):1743–1760.e11. doi: 10.1016/j.molcel.2023.03.027 (PMC10205078; doi:10.1016/j.molcel.2023.03.027)
Supplement: Supplementary file 1 [file mmc4.pdf]

Technology

Molecular Cell

Modular antibodies reveal DNA damage-induced mono-ADP-ribosylation as a second wave of PARP1 signaling

Graphical abstract

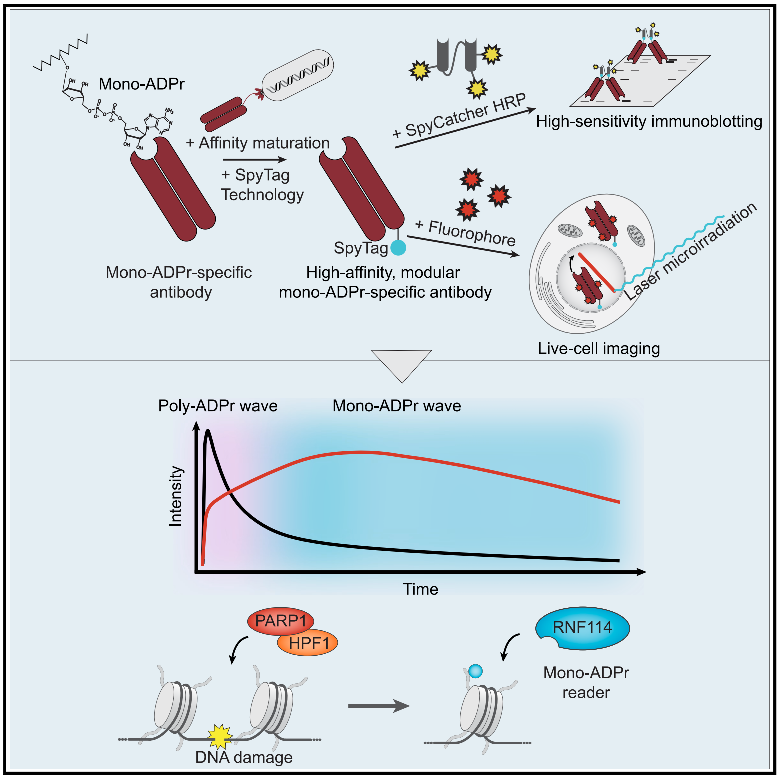

Highlights

- A broadly applicable technology for sensitive and versatile detection of mono-ADPr
- Fluorescent probes reveal serine mono-ADPr as a second wave of PARP1 signaling
- Multilevel chromatin proteomics identifies histone mono-ADPr readers
- RNF114 is a mono-ADPr reader in telomere maintenance and DNA repair signaling

Authors

Edoardo José Longarini, Helen Dauben, Carolina Locatelli, ..., Roderick J. O'Sullivan, Sébastien Huet, Ivan Matic

Correspondence

sebastien.huet@univ-rennes.fr (S.H.), imatic@age.mpg.de (I.M.)

In brief

Longarini et al. generate high-affinity and sensitivity antibodies for mono-ADP-ribosylation detection in various applications and show that serine mono-ADP-ribosylation constitutes a second wave of PARP1 signaling. They reveal a role of mono-ADPr as a recruitment signal for RNF114, modulating the DNA damage response and telomere maintenance.

Longarini et al., 2023, Molecular Cell *83*, 1743–1760
May 18, 2023 © 2023 The Author(s). Published by Elsevier Inc.
https://doi.org/10.1016/j.molcel.2023.03.027

CellPress

Molecular Cell

CellPress
OPEN ACCESS

Technology

Modular antibodies reveal DNA damage-induced mono-ADP-ribosylation as a second wave of PARP1 signaling

Edoardo José Longarini,[1] Helen Dauben,[1] Carolina Locatelli,[1] Anne R. Wondisford,[2] Rebecca Smith,[3,10] Charlotte Muench,[1] Andreas Kolvenbach,[1] Michelle Lee Lynskey,[2] Alexis Pope,[1] Juan José Bonfiglio,[1,11] Eva Pinto Jurado,[3,4,5] Roberta Fajka-Boja,[4,6] Thomas Colby,[1] Marion Schuller,[7] Ivan Ahel,[7] Gyula Timinszky,[4] Roderick J. O'Sullivan,[2] Sébastien Huet,[3,8,*] and Ivan Matic[1,9,12,*]

[1]Research Group of Proteomics and ADP-Ribosylation Signaling, Max Planck Institute for Biology of Ageing, 50931 Cologne, Germany
[2]Department of Pharmacology and Chemical Biology, UPMC Hillman Cancer Center, University of Pittsburgh, Pittsburgh, PA, USA
[3]Univ Rennes, CNRS, IGDR (Institut de Génétique et Développement de Rennes) – UMR 6290, BIOSIT (Biologie, Santé, Innovation Technologique de Rennes) – UMS 3480, US 018, 35000 Rennes, France
[4]Laboratory of DNA Damage and Nuclear Dynamics, Institute of Genetics, Biological Research Centre, Eötvös Loránd Research Network (ELKH), 6276 Szeged, Hungary
[5]Doctoral School of Multidisciplinary Medical Sciences, University of Szeged, 6276 Szeged, Hungary
[6]Department of Immunology, Albert Szent-Györgyi Medical School, Faculty of Science and Informatics, University of Szeged, 6720 Szeged, Hungary
[7]Sir William Dunn School of Pathology, University of Oxford, Oxford OX1 3RE, UK
[8]Institut Universitaire de France, Paris, France
[9]Cologne Excellence Cluster for Stress Responses in Ageing-Associated Diseases (CECAD), University of Cologne, 50931 Cologne, Germany
[10]Present address: Sir William Dunn School of Pathology, University of Oxford, Oxford OX1 3RE, UK
[11]Present address: Large Molecule Research, Pharma Research and Early Development, Roche Innovation Center Munich, 82377 Penzberg, Germany
[12]Lead contact
*Correspondence: sebastien.huet@univ-rennes.fr (S.H.), imatic@age.mpg.de (I.M.)
https://doi.org/10.1016/j.molcel.2023.03.027

SUMMARY

PARP1, an established anti-cancer target that regulates many cellular pathways, including DNA repair signaling, has been intensely studied for decades as a poly(ADP-ribosyl)transferase. Although recent studies have revealed the prevalence of mono-ADP-ribosylation upon DNA damage, it was unknown whether this signal plays an active role in the cell or is just a byproduct of poly-ADP-ribosylation. By engineering SpyTag-based modular antibodies for sensitive and flexible detection of mono-ADP-ribosylation, including fluorescence-based sensors for live-cell imaging, we demonstrate that serine mono-ADP-ribosylation constitutes a second wave of PARP1 signaling shaped by the cellular HPF1/PARP1 ratio. Multilevel chromatin proteomics reveals histone mono-ADP-ribosylation readers, including RNF114, a ubiquitin ligase recruited to DNA lesions through a zinc-finger domain, modulating the DNA damage response and telomere maintenance. Our work provides a technological framework for illuminating ADP-ribosylation in a wide range of applications and biological contexts and establishes mono-ADP-ribosylation by HPF1/PARP1 as an important information carrier for cell signaling.

INTRODUCTION

PARP1, a much-studied target for cancer therapy, plays key roles in the DNA damage response (DDR) by covalently transferring ADP-ribose from NAD^+ to a target substrate, generating ADP-ribosylation (ADPr).[1] For more than 50 years, this enzyme has been studied exclusively in the context of poly-ADPr on aspartate and glutamate.[2–4] The unexpected identification of histone serine ADPr (Ser-ADPr) marks[5] has quickly led to the establishment of serine as the primary target residue for PARP1 upon DNA damage[6–9] and to the discovery of HPF1[10] (in complex with PARP1) and ARH3 as the writer and the eraser, respectively, of Ser-ADPr.[6,9,11] When bound to PARP1, HPF1 catalyzes the addition of single units of ADP-ribose to serine residues while blocking their addition to poly-ADPr chains.[12] HPF1 rapidly dissociates, permitting PARP1 to extend the initial modification to poly-ADP-ribose.[13,14] Recent reports have shown the prevalence of cellular mono-ADPr upon DNA damage[15,16] and the impact of specific histone mono-ADPr marks on chromatin structure in biochemical assays.[17,18] However, its cellular

This is an open access article under the CC BY license (http://creativecommons.org/licenses/by/4.0/).

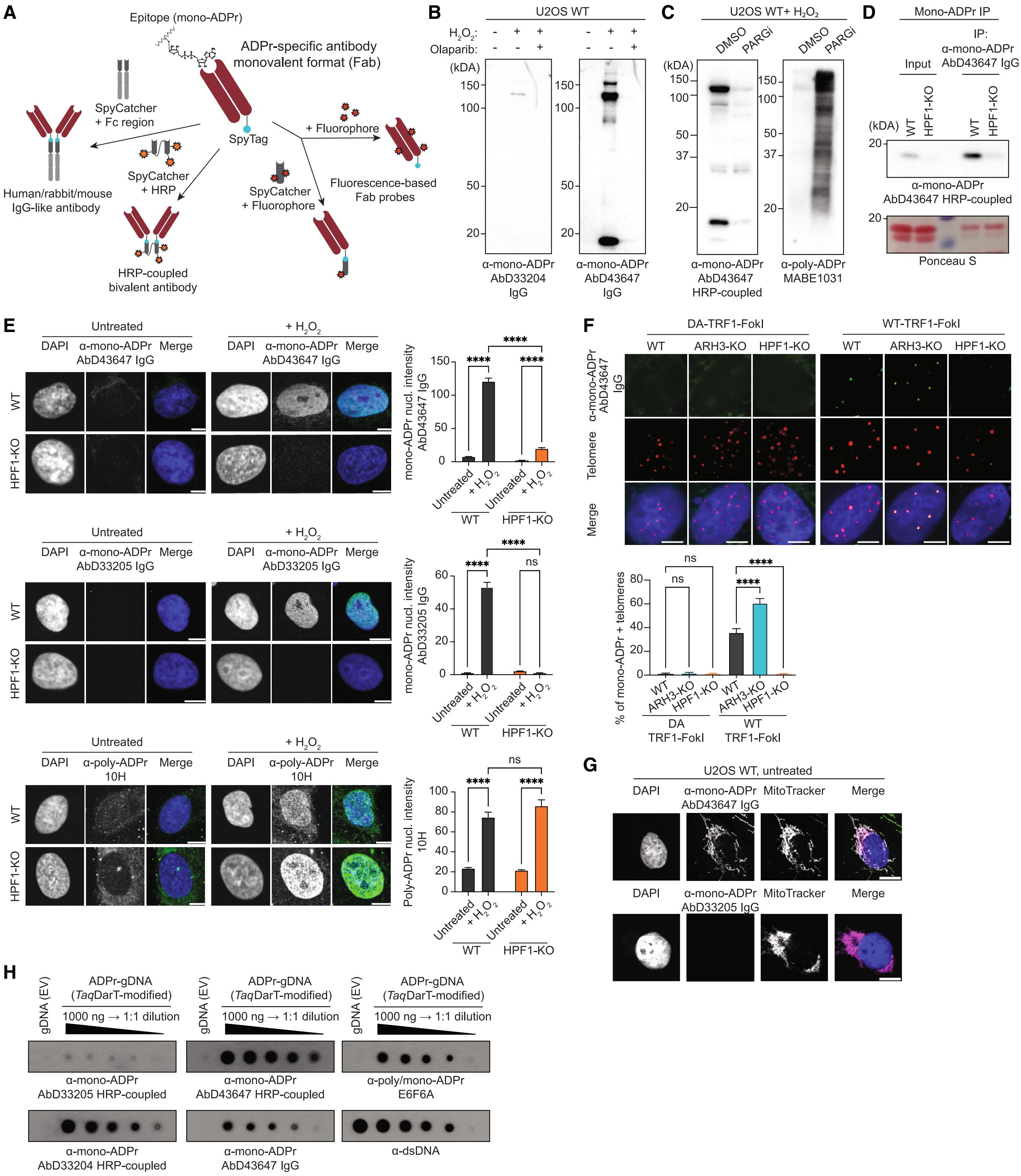

Figure 1. SpyTag-based modular antibodies enable the sensitive and versatile detection of mono-ADPr

(A) Schematic illustration of modular antibody engineering: a monovalent Fab antibody with the SpyTag peptide is covalently conjugated to functionalized SpyCatchers to generate various formats, including but not limited to "IgG-like" antibodies with different Fc chains (i.e., rabbit, mouse, and human), HRP-coupled antibodies, and fluorophore-coupled antibodies ("Fab probe").

(B) Mono-ADPr immunoblotting with AbD33204 or AbD43647.

(C) Specificity of AbD43647 by immunoblotting. See also Figure S2A.

(D) Immunoprecipitation and immunoblotting of histones with AbD43647 (top panel). Histone levels by Ponceau staining (bottom panel).

(legend continued on next page)

CellPress
OPEN ACCESS

abundance was interpreted as an intermediate in the formation and/or degradation of poly-ADPr, and direct cellular roles of mono-ADPr by HFP1/PARP1 remained unknown. Here, we consider the possibility that monomeric ADPr is a fully fledged histone mark, written and erased by dedicated enzymes, and recognized by specific effectors. To test this hypothesis and enable sensitive, versatile mono-ADPr detection both within and beyond PARP1 signaling, we have developed modular antibodies based on SpyTag technology.

DESIGN

Despite the clinical development of PARP inhibitors and their broad biological significance, the chemical nature of ADPr has long hampered our ability to study this PTM. Accordingly, considerable efforts have recently been invested in developing new tools for ADPr research. The Kraus laboratory has pioneered the conversion of protein domains recognizing ADPr into antibody-like reagents.[19] Moreover, sophisticated approaches have improved the chemical synthesis of ADP-ribosylated substrates,[20] and our phospho-guided enzymatic strategy has provided antigens for generating broad- and site-specific antibodies.[15] However, detection of ADPr in cellular contexts is still in its infancy compared with other PTMs. Although it is becoming clear that most PARPs and other transferases conjugate monomeric ADPr,[21] researchers still lack a widely applicable toolbox for sensitive detection of this modification in cellular contexts.

Given the difficulties in generating conventional anti-ADPr antibodies, most currently available tools are recombinant, either domain-based reagents or phage-display antibodies.[15,19,22] We aimed to exploit the recombinant nature of these tools to expand their functionality by applying SpyTag technology.[23] In this protein ligation system, the SpyTag peptide and the SpyCatcher domain covalently bond when brought together; hence, antibodies generated as SpyTagged antigen-binding fragments (Fabs) can be ligated to various domains and chemical labels via the SpyCatcher, yielding an expandable library of antibody formats.[24] The availability of the SpyCatcher reagents allows the implementation of this strategy in any biological laboratory. Here, we have developed an entire toolkit for flexible and sensitive detection of mono-ADPr (Figure 1A) by applying affinity maturation and the SpyTag/SpyCatcher system to our previously generated antibodies[15] and show the utility of these modular antibodies in immunoblotting, immunofluorescence, immunoprecipitation, enzyme-linked immunosorbent assay (ELISA), and live-cell imaging.

RESULTS

SpyTag-based modular antibodies enable sensitive and versatile detection of mono-ADPr

Although detection of ADPr has been significantly advanced by recent introduction of specific tools,[15,19,22,25] important ADPr events have remained undetectable. A striking illustration is that histone mono-ADPr was undetectable in the absence of DNA damage, although the high levels detected in undamaged cells lacking the serine mono-ADP-ribose hydrolase ARH3[14,15,26] indicate that it is constantly formed. For broader investigations of mono-ADPr, we have re-engineered the recombinant antibodies generated with our HPF1/PARP1-based chemical biology approach.[15] To increase their sensitivity and versatility, we applied a modular platform[24] based on the SpyTag system[23] to couple multiple features onto Fabs (Figure 1A). Among the formats we obtained for all our antibodies,[15] site-directed labeling with three copies of horseradish peroxidase (HRP) through SpyTag coupling[24] increased the immunoblotting sensitivity dramatically compared with the conventional IgG format (Figure S1A; Methods S1). We further improved the detection of mono-ADPr by using our serine mono-ADP-ribosylated peptides[15] for phage-display-based affinity maturation of AbD33204, whose binding affinity was ~4.4 μM,[15] to obtain AbD43647, a mono-ADPr-specific SpyTag antibody with a binding affinity of 248 nM and very minor unspecific binding (Figures 1B and S1B–S1K). Even when the cellular levels of poly-ADPr were massively boosted by inhibiting the poly-ADPr eraser PARG, AbD43647 did not show any cross-reactivity toward poly-ADPr (Figures 1C and S2A). An ELISA competition assay indicates that binding of AbD43647 to ADP-ribose requires adenine and a free 2′-hydroxyl group on the adenine-proximal ribose (Figure S2B). The high affinity of AbD43647 together with the HRP-conjugated format for immunoblotting rendered HPF1-dependent histone mono-ADPr detectable in WT cells in the absence of exogenous DNA damage (Figure 1D). The signal was significantly improved when immunoblotting was preceded by immunoprecipitation with AbD43647 IgG, illustrating the synergy of multiple conjugate formats of an affinity-matured antibody to maximize mono-ADPr detection (Figures 1D, S2C, and S2D). Upon H_2O_2 treatment, mono-ADPr was dramatically reduced in HPF1-KO cells, compared with WT cells (Figures 1E, S2E, and S2F), and restored by HPF1-WT expression in HPF1-KO cells, but not HPF1-E284A (Figure S2G), a catalytically inactive mutant that still interacts with PARP1.[12]

Recently, ARH3 has been implicated in ALT-mediated telomere maintenance,[14] suggesting a role for mono-ADPr at telomeres. To test this, we used the TRF1-FokI system, which specifically cleaves telomeric DNA to induce telomere DDR.[27] Our antibodies reveal the formation of mono-ADPr foci at telomeres with WT-TRF1-FokI, but not with the catalytically dead (D450A) mutant. Compared with WT cells, this mono-ADPr was increased in ARH3-KO cells and abrogated in HPF1-KO cells (Figures 1F and S2H).

To demonstrate the broad value of AbD43647, we have gone beyond PARP1 signaling and protein ADPr. In untreated cells, AbD43647 gives a clear mitochondrial signal,[28] independent of

(E) Left: immunofluorescence (IF) of mono- and poly-ADPr. Scale bars, 10 μm. Right: quantified nuclear signal intensities.

(F) Top: IF images of mono-ADPr in telomere-localized DNA damage. Bottom: quantified mono-ADPr positive telomeres (%). Scale bars, 5 μm.

(G) IF images showing mitochondria and mono-ADPr co-localization. Scale bars, 10 μm.

(H) Dot blot of genomic DNA (gDNA) ADPr by the indicated antibodies. See also Figures S1 and S2 and Methods S1. Graphs indicate mean ± SEM from a representative of 3 independent experiments. ****$p < 0.0001$; ns, not significant.

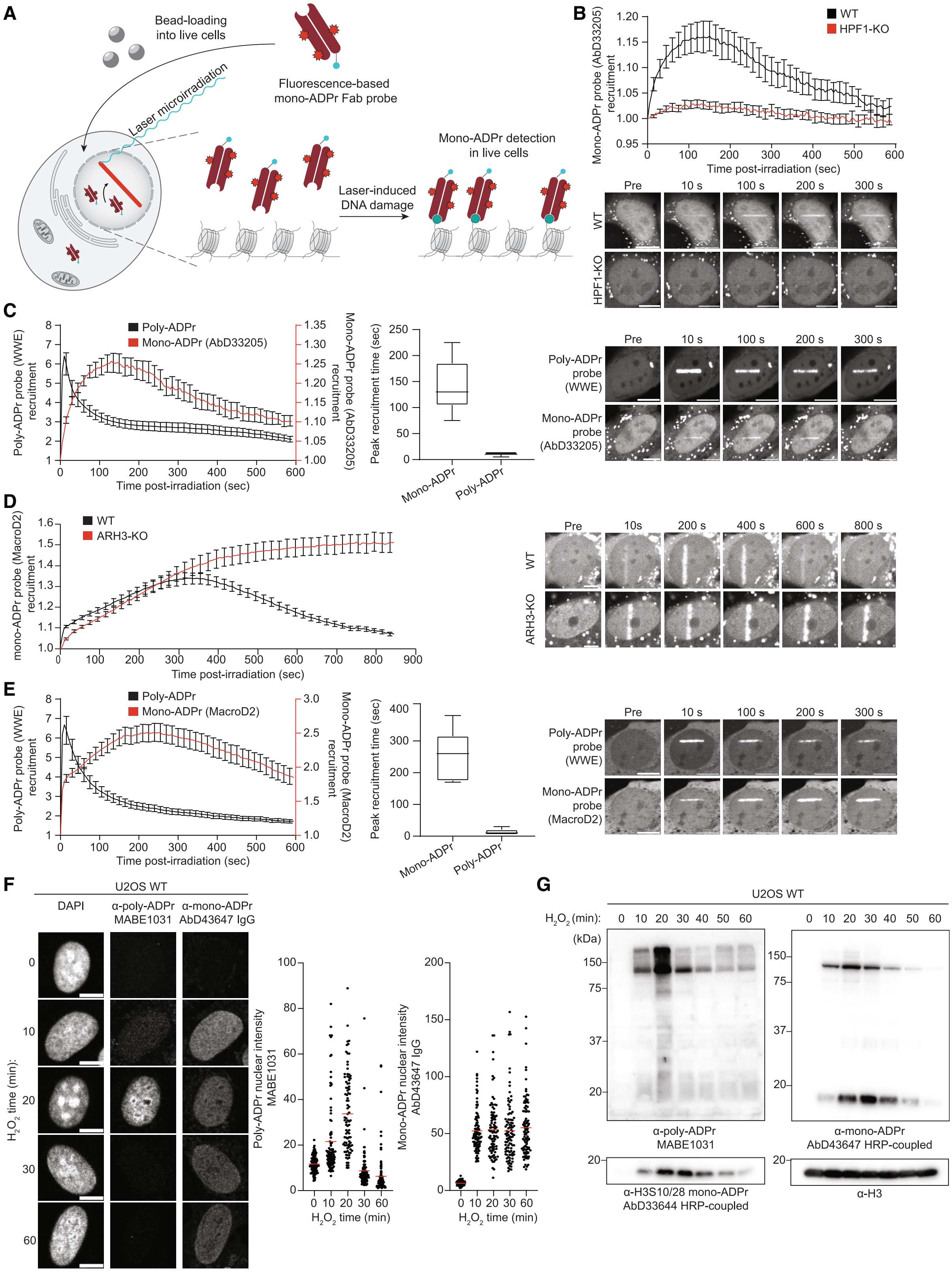

(legend on next page)

HPF1 or ARH3 and undetectable by AbD33205 (Figures 1G and S2I). Moreover, we tested our antibodies on DNA ADPr.[29] Although AbD33205 detects ADP-ribosylated DNA weakly, AbD43647 gives a strong signal, especially in the HRP-coupled format (Figures 1H and S2J). Thus, the range of applications for our new high-affinity antibody AbD43647 is much wider than for the “anti-protein mono-ADPr” AbD33205.[15]

Fluorescence-based sensors reveal DNA damage-induced serine mono-ADPr as a second wave of PARP1 signaling

With these tools for sensitive detection of endogenous mono-ADPr in various applications (Figure 1), we set out to investigate mono-ADPr dynamics in the DDR. To visualize mono-ADPr in living cells, we took advantage of the multiple formats of our SpyTagged recombinant antibodies (Figure 1A) and combined laser irradiation with bead loading, a simple method for introducing proteins into the cells.[30] Reasoning that the recruitment kinetics of mono-ADPr-specific, fluorescence-based probes should reflect the dynamics of DNA damage-induced mono-ADPr in living cells, we conjugated the Fab version of AbD33205 to a fluorescent dye (Figure S3A). A key feature of the Fab format is its small size, allowing the sensor to enter the nucleus after bead loading into the cytoplasm (Figures 2A, S3B, and S3C). To test the specificity of our Fab-based probe, we treated the cells with olaparib and observed that PARP1 inhibition abolished the accumulation of the mono-ADPr sensor at DNA damage sites (Figure S3D). Recruitment of the probe was also abolished in HPF1-KO cells (Figure 2B), corroborating the dependence of serine mono-ADPr on HPF1 in cells (Figure 1E). PARP1 signaling is one of the earliest pathways activated during the DDR, as illustrated by the rapid formation of poly-ADPr upon DNA damage.[31,32] Surprisingly, we observed a more gradual mono-ADPr response (Figures 2B and S3D). To directly compare the dynamics of these two forms of ADPr in living cells, we bead loaded the anti-mono-ADPr fluorescent Fab and expressed GFP-WWE, an established probe for poly-ADPr.[25,31,33] Strikingly, live-cell microscopy revealed that, in contrast to the ultrafast, transient formation of poly-ADP-ribose, mono-ADPr levels increase more gradually within the first few minutes after damage before reaching a plateau and then slowly declining (Figure 2C). To confirm these observations, we used the macrodomain of MacroD2, which is specific for mono-ADPr and unable to hydrolyze Ser-ADPr,[11,34] as a fluorescence-based sensor for mono-ADPr in live-cell imaging. In ARH3-KO cells, which have high and long-lasting levels of mono-ADPr upon DNA damage,[14,15,26] the recruitment of this macrodomain persists longer compared with WT cells, reaching a plateau 6 min after damage (Figure 2D). By contrast, its recruitment is either abolished or greatly diminished in the olaparib-treated and HPF1-KO cells, respectively (Figures S3E and S3F),[10,34] establishing this macrodomain construct as a second sensor of mono-ADPr. The accumulation of this probe confirmed that mono-ADPr is a delayed, persistent signal upon DNA damage induction by microirradiation (Figure 2E), in line with the results obtained with our Fab-based fluorescent sensor (Figure 2C). Although there are differences in the precise recruitment kinetics between these two probes (Figures 2C and 2D), this is not surprising considering the varied binding preferences of each mono-ADPr-binding tool, the distinct dynamics of different mono-ADPr targets[15] and the possibility that MacroD2 hydrolyzes mono-ADPr at non-serine residues.[34] We further corroborated our observation of transient poly-ADPr and persistent mono-ADPr by immunoblotting and immunofluorescence using orthogonal detection reagents. In H_2O_2-treated cells, poly-ADPr peaks at 20 min followed by a rapid decline. Conversely, mono-ADPr peaks at 30 min and remains elevated much longer (Figures 2F and 2G).

Together, these results demonstrate that, upon DNA damage, serine mono-ADPr is a delayed, persistent signal in the DDR distinct from poly-ADPr, implicating this modification as the second wave of PARP1 signaling and suggesting a dual role for PARP1 as both an early and late responder to DNA damage.

Cellular HPF1/PARP1 ratios regulate mono-ADPr levels

Considering the distinctive poly- and mono-ADPr dynamics observed (Figure 2), we were intrigued by the recent report that HPF1 dissipates from DNA lesions more slowly than PARP1,[32] a behavior reminiscent of prolonged mono-ADPr. Given the dependence of mono-ADPr on HPF1 (Figures 1E and 2B), we hypothesized that the HPF1/PARP1 ratio dynamically regulates the mono-ADPr response to DNA damage, supported by the increased generation of mono-ADPr by higher concentrations of HPF1 observed in biochemical reactions (Figure 3A).[13,35] Strikingly, we observe a dramatic increase in cellular mono-ADPr upon HPF1 overexpression (Figures 3B and 3C). By live-cell imaging, we detect much higher mono-ADPr levels at laser tracks when we overexpress HPF1-WT compared with HPF1-E284A, demonstrating that the increase in mono-ADPr relies on the catalytic activity of HPF1 (Figure 3D). In ARH3-KO cells, where mono-ADPr is already elevated under basal conditions,[14,15,26] overexpression of HPF1 further increased the mono-ADPr levels (Figure 3E). Collectively, these findings demonstrate that mono-ADPr depends on cellular HPF1/PARP1 molarity ratios, implying the transient association of HPF1 to PARP1[12,13] as a mechanism regulating the cellular

Figure 2. Fluorescence-based sensors reveal DNA damage-induced serine mono-ADPr as second wave of PARP1 signaling
(A) Real-time live-cell detection of mono-ADPr by bead-loaded Fab antibodies.
(B–E) Recruitment kinetics and representative confocal images of: (B) mono-ADPr Fab probe (fluorophore-coupled AbD33205), scale bars, 10 μm.
(C) Genetically encoded poly-ADPr probe (RNF146 WWE domain), scale bars, 10 μm.
(D) Genetically encoded mono-ADPr probe (macrodomain of MacroD2), scale bars, 5 μm.
(E) Poly- and mono-ADPr probes, scale bars, 10 μm.
(F) Left: IF images of WT U2OS cells, treated with H_2O_2 for the indicated times. Right: quantified mean nuclear intensity from mono- or poly-ADPr antibodies. Scale bar, 10 μm.
(G) Immunoblotting of WT U2OS cells treated with H_2O_2 for the indicated time. See also Figure S2. Data in (B)–(E) are shown as mean ± SEM from a representative of 3 independent experiments.

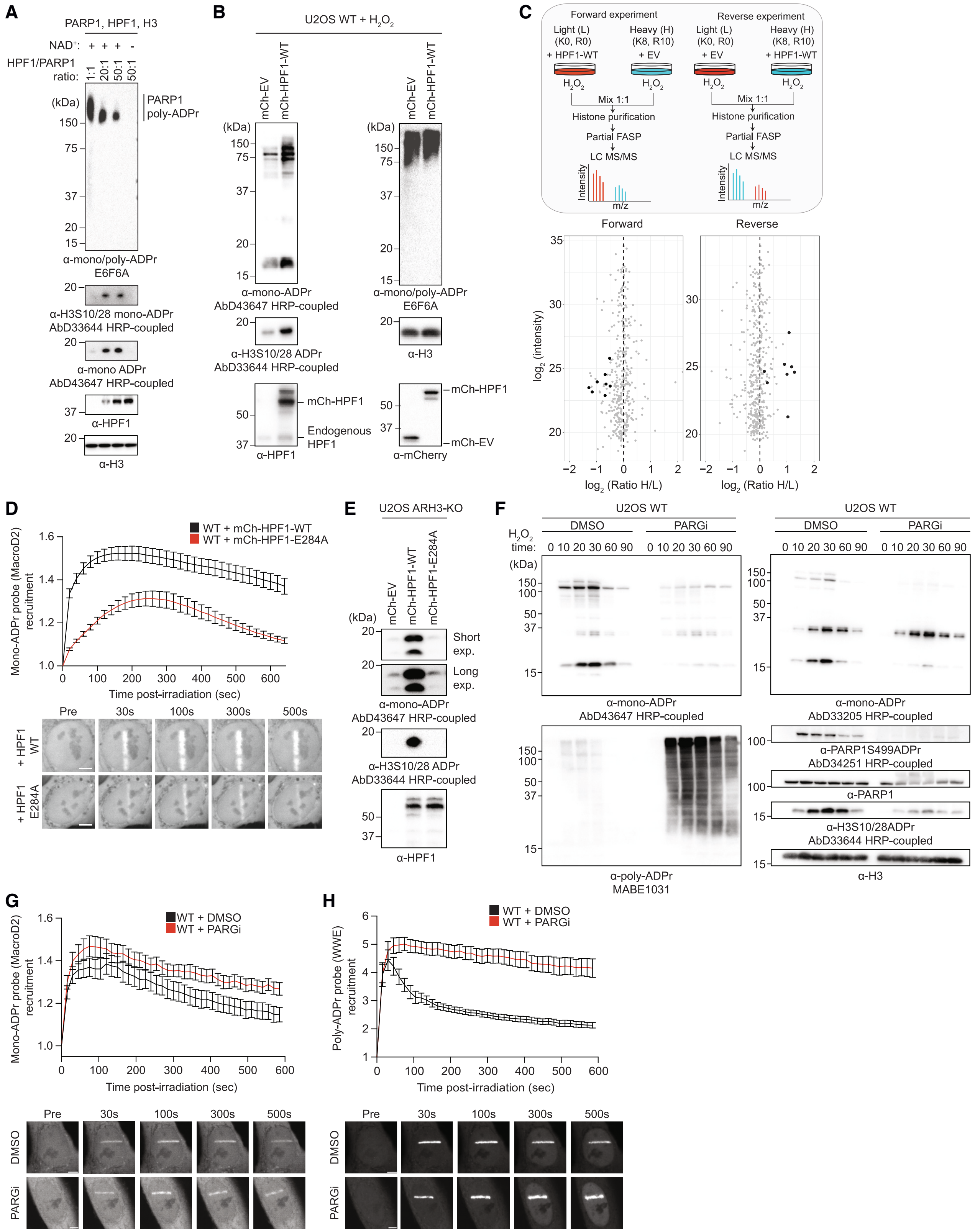

(legend on next page)

levels of serine mono-ADPr (Figures 3B–3E). Although it was recently reported that HPF1 modulates the number and length of poly-ADP-ribose chains present at DNA lesions,[32] our modular antibodies reveal the specific effect of HPF1 on cellular mono-ADPr.

Having explored regulation of mono-ADPr at the initiation phase, we investigated whether the mono-ADPr wave results from poly-ADPr degradation. Previously, we have shown that mono-ADPr partly depends on PARG,[15] although the precise dynamic and substrate specificity were not defined. In a time-course western blotting experiment, we discovered that the degree of dependence on PARG differs for mono-ADPr substrates (Figure 3F). Consistent with our previous study,[15] mono-ADPr on PARP1 and core histones largely depends on PARG. Intriguingly, mono-ADPr of other primary substrates is virtually independent of PARG (Figure 3F) and, therefore, must result directly from initial serine mono-ADPr. When specifically looking at mono-ADPr dynamics at DNA lesions, we observed that prolonged mono-ADPr is not decreased by PARG inhibition, indicating that mono-ADPr is not a remnant of poly-ADPr (Figures 3G and 3H). Thus, although mono-ADPr on PARP1, most of which is not on chromatin,[14] originates from poly-ADPr removal, the HPF1/PARP1-dependent mono-ADPr wave at DNA lesions appears to be independent of PARG action.

Identification of mono-ADPr readers by chromatin proteomics

Our evidence of serine mono-ADPr as a second wave of PARP1 signaling controlled in cells by the HPF1/PARP1 ratio (Figures 2 and 3) prompted us to determine the consequences of this PTM as a regulatory signal, complementary to but distinct from poly-ADPr. Considering its dynamics at DNA lesions, we reasoned that mono-ADPr might function as a recruitment signal at later stages of DNA repair signaling. To identify putative readers of histone mono-ADPr, we applied three complementary proteomics approaches. For two strategies, we broadened our chemical biology strategy,[15] generating three biotinylated ADP-ribosylated peptides (H3S10ADPr, H3S28ADPr, and H4S1ADPr) corresponding to primary histone mono-ADPr marks[5] for a SILAC-based peptide pull-down approach (Figure 4A). Among the interactors, we identified DTX3L/PARP9, a complex that binds mono- and oligo-ADPr and mediates the attachment of ADP-ribose to ubiquitin,[36,37] and ALC1, an established poly-ADPr binder.[38–40] In addition, we identified proteins that were unknown as ADPr interactors, including ALYREF/THOC4 and SIRT6 (Figures 4B, S4A, and S4B; Table S1), which is intriguing, given the SIRT6 participation in BER and DSB repair[41,42] and its promotion of XRCC1 and POLB recruitment to DNA damage.[33]

Next, to probe mono-ADPr interactors in a more physiological context, we generated nucleosomes carrying mono-ADPr on H3S10. Although specialized approaches for generating ADP-ribosylated nucleosomes were recently proposed,[17,35] we designed an approach combining our phospho-guided strategy[15] to ADP-ribosylate long peptides site-specifically and a widely accessible approach for ligating peptides to a fully assembled tailless nucleosome (Figures 4C and S4C–S4F). Nucleosome pull-down screens unexpectedly revealed several members of the Septin family as interactors of H3S10 mono-ADP-ribosylated nucleosomes (Figures 4D and S4G; Table S1). Immunoblotting after nucleosome pull-down confirmed mono-ADPr binding of SEPT2 and SIRT6, an interactor identified only with peptide pull-down-based proteomic analyses (Figures S4G and S4H). This suggests that in comparison with peptide pull-downs, the abundant recombinant histones hamper proteomic identification of low-abundance mono-ADPr interactors and underscores the complementarity of these two approaches.

Finally, to identify proteins whose recruitment to chromatin is regulated specifically by mono-ADPr directly in cells, we combined quantitative proteomics of chromatin-associated proteins with targeted modulations of cellular mono-ADPr described above (Figures 4E, S4I, and S4J). As expected, upon H_2O_2 treatment, we observed a broad rearrangement of the chromatin-associated proteome (Figure 4F; Table S1) and enrichment of proteins involved in the DDR (Figure S4K), validating our approach. We reasoned that we could specifically determine the mono-ADPr-dependent chromatin-associated proteome by analysis of H_2O_2-treated HPF1-KO cells complemented with HPF1-WT or HPF1-E284A. Two ubiquitin E3 ligases, RNF114 and the DTX3L/PARP9 complex, were exclusively enriched on chromatin under HPF1-WT overexpression compared with HPF1-E284A overexpression (Figure 4G). To explore the chromatin-associated proteome beyond exogenously induced DNA damage, we abolished mono-ADPr in ARH3-KO cells with olaparib (Figure S4L) and observed reduced chromatin associations of RNF114 and DTX3L/PARP9, as well as several known PARP1-modulated proteins, including XRCC1, LIG3, and APLF (Figure 4H). Direct comparisons between WT and ARH3-KO cells revealed that chromatin binding of endogenous RNF114 and DTX3L/PARP9 is controlled by mono-ADPr (Figures 4I, 4J, and S4L; Table S1). To corroborate the interactions of DTX3L and RNF114 with mono-ADPr, we immunoprecipitated GFP-RNF114 and GFP-DTX3L and observed co-precipitation of

Figure 3. Cellular HPF1/PARP1 ratios regulate mono-ADPr levels

(A) Immunoblotting of *in vitro* HPF1/PARP1 ADPr reactions with increasing concentrations of recombinant HPF1.

(B) Immunoblotting of WT U2OS cells transfected with mCherry-empty vector (mCh-EV) or mCherry-HPF1-WT (mCh-HPF1-WT) and H_2O_2 treated.

(C) Top: schematics of SILAC-based proteomics of histone mono-ADPr marks on HPF1 overexpression and H_2O_2 treatment. Bottom: scatterplot showing SILAC quantification. Mono-ADPr peptides (black) and other peptides (gray).

(D) Top: mono-ADPr probe recruitment kinetics in WT U2OS cells overexpressing mCherry-HPF1-WT (black) or mCherry-HPF1-E284A (red). Bottom: representative confocal images.

(E) Immunoblotting of ARH3-KO U2OS cells transfected with mCherry-EV, mCherry-HPF1-WT, or mCherry-HPF1-E284A.

(F) Immunoblotting showing mono-ADPr levels on PARGi and H_2O_2 time-course treatment.

(G) Top: mono-ADPr probe recruitment kinetics in WT U2OS cells treated with DMSO (black) or PARGi (red). Bottom: representative confocal images.

(H) Top: poly-ADPr probe recruitment kinetics in WT U2OS cells treated with DMSO (black) or 1 μM PARGi (red). Bottom: representative confocal images. Data in (D), (G), and (H) are shown as mean ± SEM from a representative of 3–4 independent experiments. Scale bars, 5 μm.

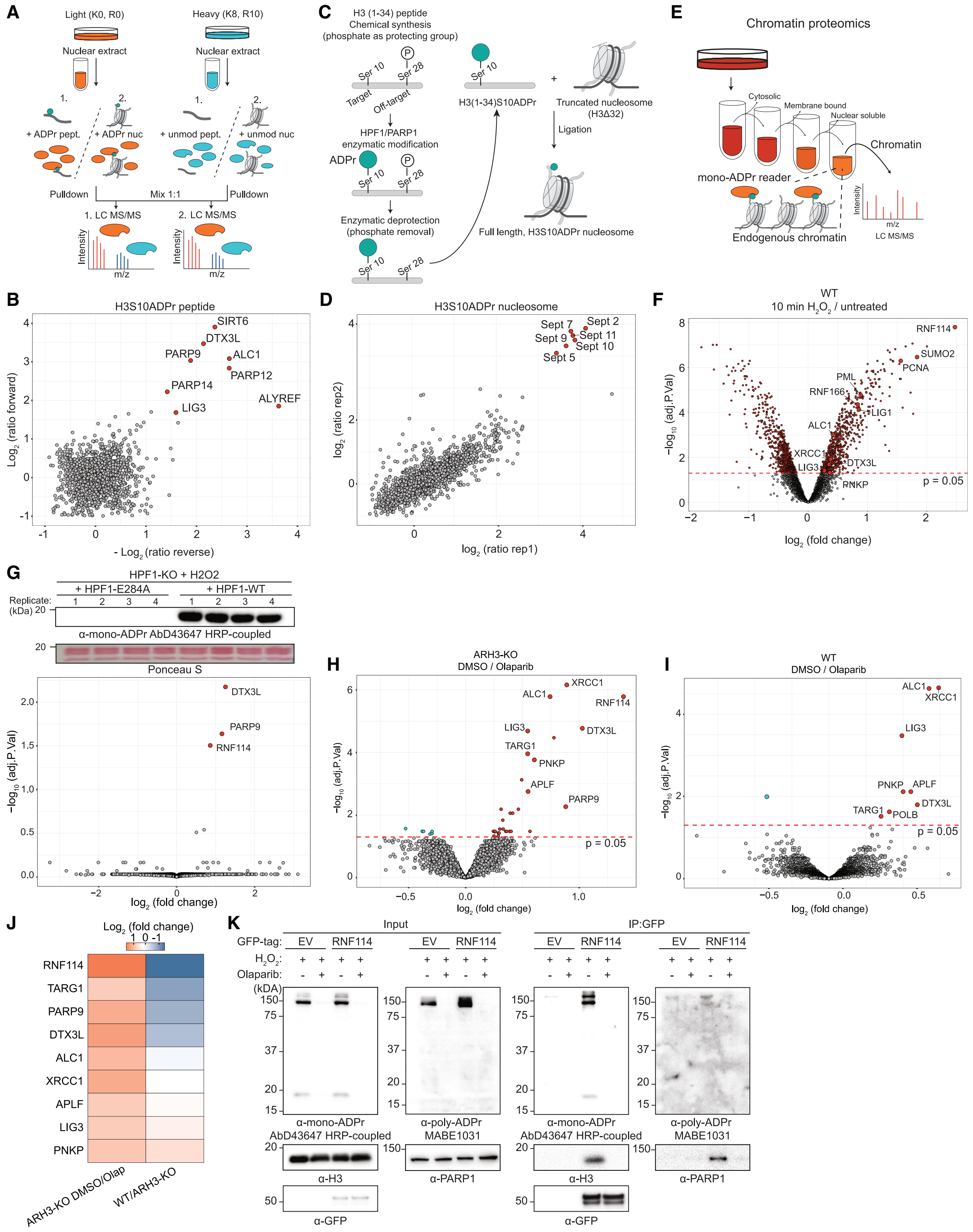

(legend on next page)

mono-ADPr-ribosylated H3 and PARP1, interactions that were abolished by olaparib (Figures 4K and S5A–S5C). Using AbD33644, our site-specific H3S10/S28mono-ADPr antibody,[15] we observed that these sites are recognized by RNF114 and that serine-to-alanine substitutions almost completely disrupted the interaction (Figures S5D and S5E). Continuous H_2O_2 treatment in ARH3-KO cells produces persistently high levels of mono-ADPr up to 90 min after induction of treatment,[14,15] and accordingly, proteomics revealed greater levels of chromatin-bound RNF114 and DTX3L/PARP9 after 90 min of H_2O_2 treatment, which were abolished by olaparib (Figure S5F). Under such high levels of mono-ADPr, we also observed recruitment of other known ADPr binders, such as PARP12,[40] to chromatin. These observations are not limited to DNA damage by H_2O_2, since methylmethanesulfonate (MMS) treatment also robustly enriched RNF114 on chromatin (Figure S5G). Collectively, these proteomics datasets provide a systematic overview of protein binding to chromatin mono-ADPr on physiologically relevant substrates and represent a resource for future studies.

Chromatin mono-ADPr functions as a recruitment signal for RNF114

Among the identified putative readers of mono-ADPr (Figure 4), we were particularly intrigued by RNF114.[43] To better understand its chromatin dynamics in the context of other chromatin-associated DNA damage responders, we performed time-resolved chromatin proteomics experiments following H_2O_2 treatment. Chromatin association of RNF114 peaked at 20 min and continued until at least 60 min after the treatment, a behavior consistent with mono-ADPr dynamics (Figures 5A, S6A, and S6B; Table S1). Next, we sought to characterize RNF114 accumulation at DNA lesions in living cells. GFP-RNF114 accumulated at the sites of damage within 2 min and persisted for >10 min with a slowly decreasing plateau (Figure 5B), in line with the chromatin association of endogenous RNF114 observed in proteomics analyses (Figures 5A and S6A). RNF114 recruitment was abolished by olaparib, indicating PARP1 dependence, and greatly enhanced in HPF1-KO cells expressing HPF1-WT compared with HPF1-E284A (Figures 5B, 5C, and S6C), consistent with higher mono-ADPr levels observed at DNA lesions upon HPF1-WT overexpression (Figure S3F). In ARH3-KO cells, with persistent mono-ADPr at DNA lesions (Figure 2D), we observed enhanced late accumulation of RNF114 at the sites of damage (Figure 5D). To confirm that mono-ADPr drives RNF114 accrual at DNA lesions, we reasoned that, due to the high activity of PARG, late inhibition of PARP1 would abolish poly-ADPr but have only a minor effect on mono-ADPr. As expected, late olaparib treatment resulted in fast degradation of poly-ADPr demonstrated by the rapid decrease in the levels of WWE and the poly-ADPr reader APLF (Figures 5E and 5F). In contrast, mono-ADPr remained largely unaffected, and importantly, RNF114 recruitment was not impaired by late olaparib treatment (Figures 5G and 5H). Conversely, abolishing mono-ADPr by ARH3 overexpression while preserving poly-ADPr prevented the recruitment of RNF114 (Figures 5I–5K). In addition, although the poly-ADPr binders ALC1 and APLF[38,44] are immediately recruited to DNA lesions, accumulation of RNF114 clearly follows the dynamics of the mono-ADPr wave (Figures 5L and S6D). Altogether, these data provide conclusive evidence that mono-ADPr recruits RNF114 to DNA lesions. Next, considering our chromatin proteomics analysis of ARH3-KO cells (Figures 4H–4J), we expected RNF114 recruitment to chromatin under physiological conditions when histone mono-ADPr is elevated in the absence of exogenous DNA damage. Using fluorescence correlation spectroscopy (FCS),[45] we observed a reduced diffusion of RNF114 and the mono-ADPr binding domain in the ARH3-KO compared with WT cells, suggesting increased binding to mono-ADP-ribosylated chromatins. This effect was abolished by long-term olaparib treatment and boosted by HPF1 overexpression, whereas the recruitment of the poly-ADPr binder APLF was unaffected (Figures 5M and S6E), indicating that mono-ADPr-mediated chromatin association of proteins might represent a response to HPF1/PARP1 signaling beyond the repair of exogenous DNA damage. Overall, these results demonstrate that recruitment and chromatin retention of RNF114 depends on mono-ADPr.

RNF114 recruitment to DNA lesions is mediated by its zinc-finger domains

To determine whether RNF114 directly interacts with serine mono-ADPr, we tested the ability of recombinant RNF114 to bind a histone mono-ADP-ribosylated peptide.[15] Although RNF114 has not been reported to bind serine mono-ADPr, we were encouraged by evidence of mono-ADPr-dependent interaction between RNF114 and PARP10.[46] We observed binding

Figure 4. Identification of mono-ADPr readers by chromatin proteomics

(A) Quantitative proteomics workflows to identify interactomes of Ser-mono-ADPr peptides (1) and H3S10ADPr nucleosome (2).

(B) Scatterplot showing proteins enriched (red) by H3S10 mono-ADPr peptide compared with unmodified peptide. n = 2 biological replicates.

(C) Chemoenzymatic generation of site-specific H3S10ADPr nucleosomes.

(D) Scatterplot showing proteins enriched (red) by the H3S10ADPr nucleosome compared with unmodified nucleosome. n = 2 biological replicates.

(E) Subcellular fractionation proteomics workflows for analysis of the mono-ADPr-dependent chromatin-associated proteome.

(F) WT U2OS cells were H_2O_2-treated, and the chromatin fraction (as in E) was subjected to LC-MS/MS analysis. n = 3 biological replicates.

(G) Top: immunoblotting of HPF1-KO U2OS cells transfected with mCherry-HPF1 WT or mCherry-HPF1-E284A, treated with H_2O_2 for 20 min. Immunoblotting (top) or LC-MS/MS of chromatin fractions. Bottom: volcano plot showing the log_2-fold change of identified proteins. n = 4 biological replicates.

(H–J) ARH3-KO (H) and WT (I) U2OS cells were treated with DMSO or 1 μM olaparib for 48 h, and the chromatin fraction was subjected to LC-MS/MS. Volcano plot showing the log_2-fold change of identified proteins. (J) Heatmap showing log_2-fold change of chromatin-associated proteins in the indicated condition. Data from (H)–(J) come from the same experiment. n = 3 biological replicates.

(K) Immunoblotting of WT U2OS cells transfected with GFP-EV or GFP-RNF114, olaparib- and H_2O_2-treated then subjected to anti-GFP immunoprecipitation. For (B), (D), and (F)–(I), the red dotted line represents significance with p value = 0.05 ($-log_{10}$(adj. p value) > 1.3) cutoff. Significant proteins are indicated in red or blue. See also Figures S3–S5 and Table S1.

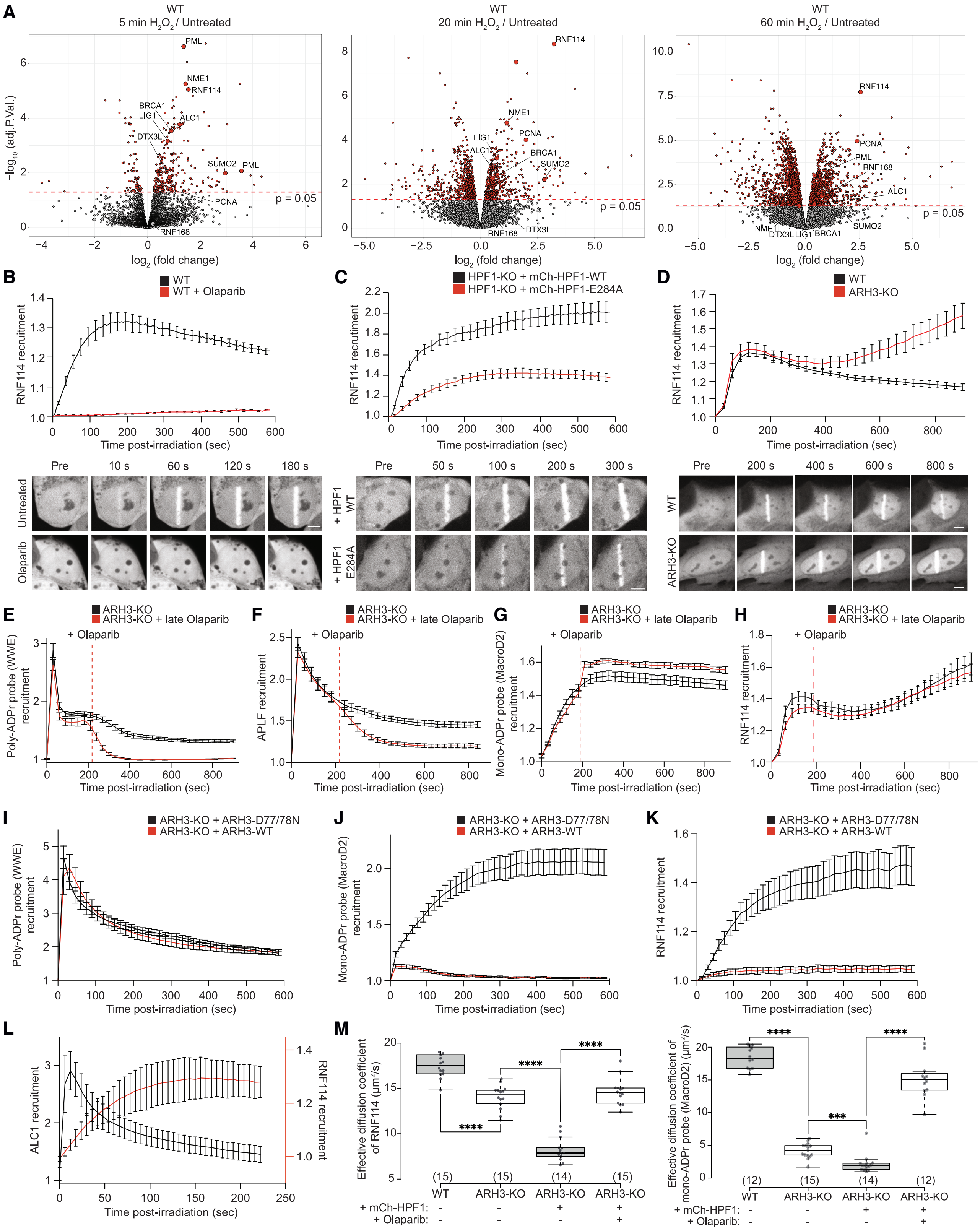

(legend on next page)

CellPress
OPEN ACCESS

to H3(1-21)S10ADPr, but not its unmodified counterpart, and only weak binding to poly-ADPr, especially when compared with known poly-ADPr binders (Figures 6A and 6B). The inability of RNF114 to bind mono-ADPr upon zinc depletion by EDTA (Figure 6A) and the existence of a PBZ motif[44] made us hypothesize that zinc fingers (Zns) of RNF114 are responsible for its mono-ADPr-dependent recruitment to DNA damage sites. To test this, we deleted the three Zns of RNF114 individually (Figures 6C and S6F) and monitored their recruitment to DNA lesions. The recruitment was abolished when either Zn2 or Zn3 was deleted, whereas the Zn1 deletion mutant mostly retained its ability to accumulate at DNA damage sites (Figures 6D and S6G). Zn2 and Zn3 are atypical C2H2 Zns that together constitute the drought-induced 19-protein type, zinc-binding domain (Di19_Zn-bd).[47] Mutation of the highly conserved cysteine C176 of Di19_Zn-bd prevented the recruitment of RNF114 to DNA damage sites (Figure 6E) and its binding to a mono-ADP-ribosylated peptide (Figure 6F). Consistently, immunoprecipitated GFP-RNF114-WT, but not GFP-RNF114-C176A, co-precipitated mono-ADP-ribosylated H3 and PARP1 (Figure 6G). From these results, we concluded that the Di19_Zn-bd domain is essential for mono-ADPr-dependent recruitment of RNF114 to DNA lesions.

RNF114 modulates the alternative lengthening of the telomeres pathway and the DDR

The loss of ARH3-KO was previously associated with impaired telomere extension by the ALT pathway,[14] suggesting a role of serine mono-ADPr in telomere maintenance. To investigate serine mono-ADPr in this process, we evaluated the formation of ALT-associated PML bodies (APBs) foci in HPF1-KO cells. Complementation with HPF1-WT rescued APB foci formation to the level of unperturbed WT cells, an effect that was not observed with HPF1-E284A (Figure 7A). These results were verified through the siRNA depletion of HPF1 in U2OS cells and LM216J, an additional ALT cancer cell line (Figure S7A). Given the occurrence of mono-ADPr at the sites of telomeric damage (Figure 1F) and its functional significance[14] (Figure 7A), we hypothesized that RNF114 could be recruited to telomeres. We observed the localization of RNF114-WT at the sites of telomeric damage in WT cells, but not in HPF1-KO cells (Figure 7B). Telomeric accumulation of RNF114 dramatically increased in ARH3-KO cells as expected, given the higher levels of mono-ADPr (Figure 1F). By contrast, GFP-RNF114-C176A failed to accumulate at damaged telomeres in WT and ARH3-KO cells (Figure 7B). Interestingly, we also observed the HPF1-dependent recruitment of other readers of mono-ADPr (Figure 4), namely SEPT6 and PARP9, to damaged telomeres (Figures S7B and S7C).

Our observation of RNF114 recruitment to damaged telomeres (Figure 7B) prompted us to investigate whether RNF114 also plays a role in telomere maintenance. Similar to siHPF1, siRNF114 alone significantly reduced the APB formation (Figures 7C, S7D, and S7E). Importantly, simultaneous siRNAs of HPF1 and RNF114 failed to further decrease the APB foci (Figures 7C, S7D, and S7E), indicating participation in the same pathway. To further investigate the role of RNF114 at telomeres, we generated the RNF114-KO cells (Figure S7F). Consistent with the results obtained with siRNAs of RNF114, APB formation is reduced in RNF114-KO cells and is rescued by the expression of RNF114-WT, but not RNF114-C176A (Figure 7D), indicating the importance of RNF114 as a mono-ADPr effector in this process. We obtained similar results with an assay to visualize the ALT telomere DNA synthesis, for which APBs are functionally important.[48] ALT DNA synthesis, measured by the number of EdU+ telomeres, is decreased in RNF114-KO cells, and RNF114-WT, but not RNF114-C176A, expression rescued this phenotype (Figure 7E). Taken together, these results suggest a role of mono-ADPr in ALT telomere maintenance and implicate RNF114 as an effector of mono-ADPr.

Next, we investigated the role of RNF114 in DDR and assessed the sensitivity of RNF114-deficient cells to DNA-damaging agents. Consistent with the role of RNF114 in the DDR, RNF114-KO cells exhibited significantly higher sensitivity to MMS and IR treatment than WT cells (Figures 7F and S7G). 53BP1 is a key regulator of non-homologous end-joining (NHEJ)-mediated double-strand break repair,[49] and PARP1 loss is associated with a reduction of 53BP1 foci accumulation.[50] Therefore, we investigated whether RNF114 plays a role in this pathway. RNF114-KO- and siRNF114-treated cells exhibited significantly reduced 53BP1 foci formation upon DNA damage (Figures 7G and S7H), without markedly altering γH2AX foci (Figures 7H and S7I). RNF114-WT, but not RNF114-C176A, rescues the reduced 53BP1 foci formation upon DNA damage in the RNF114-KO cells (Figure 7I). RNF114 depletion also significantly impaired the foci formation of RIF1, a key effector of 53BP1[49] (Figures 7K and S7J). Using a live-cell probe specific for H2AK13/15Ub,[51] the histone mark responsible for 53BP1 recruitment to DNA lesions, in RNF114-KO cells, we observed lower levels of this mark at DNA lesions (Figure 7J) and, accordingly, a decrease in foci

Figure 5. Chromatin mono-ADPr functions as a recruitment signal for RNF114

(A) Chromatin fraction analysis of H_2O_2-treated WT U2OS cells. Volcano plots showing the $\log_2$-fold change of identified proteins. Red dotted lines represent significance with p value = 0.05 ($-\log_{10}$(adj. p value) > 1.3) cutoff. Significant proteins are indicated in red. n = 4 biological replicates.

(B–D) Recruitment kinetics and representative confocal images for GFP-RNF114-WT in: (B) WT U2OS cell untreated (black) or 30 μm olaparib treated (red); (C) HPF1-KO U2OS cells expressing mCherry-HPF1-WT (black) or mCherry-HPF1-E284A (red); (D) WT (black) or ARH3-KO (red) U2OS cells. Scale bars, 5 μm.

(E–H) Recruitment kinetics of: poly-ADPr probe (E), APLF (F), mono-ADPr probe (G), and RNF114 (H) in ARH3-KO U2OS cells. Cells were treated (red) or not (black) with 30 μM olaparib 210 s after laser microirradiation.

(I–K) Recruitment kinetics of: poly-ADPr probe (I), mono-ADPr probe (J), and RNF114 (K) in ARH3-KO U2OS cells expressing mCherry-ARH3-WT (red) or mCherry-ARH3-D77/78N (black).

(L) Recruitment kinetics of GFP-RNF114 (red) and mCherry-ALC1 (black).

(M) Effective diffusion coefficient measured by FCS for GFP-RNF114 (left) and mono-ADPr probe (right). ****p value < 0.0001, ***p value < 0.001 (unpaired Student's t test assuming unequal variances). Data in (B)–(L) are shown as mean ± SEM from a representative of 3 independent experiments.

See also Figure S6 and Table S1.

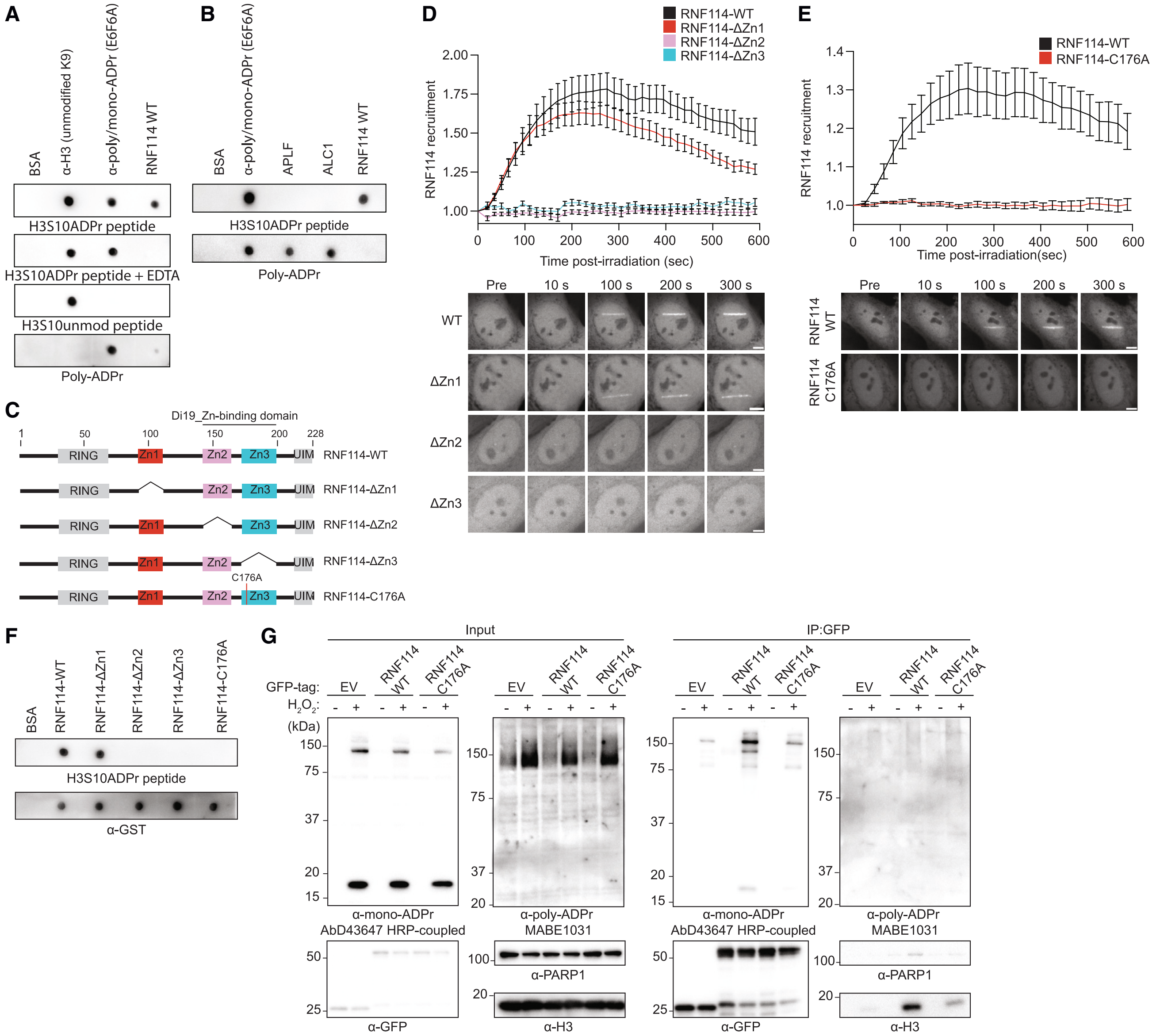

Figure 6. RNF114 recruitment to DNA lesions is mediated by its zinc-finger domains

(A) Dot blots of recombinant full-length RNF114 with indicated peptides or poly-ADP-ribose. Bovine serum albumin (BSA) and anti-mono/poly-ADPr (E6F6A) were used as negative and positive controls of ADPr binding, respectively.

(B) Dot blots of equal moles of recombinant APLF, ALC1, and RNF114.

(C) Domain architectures of RNF114 and deletion mutants. RING (RING-finger domain), Zn1 (zinc finger 1), Zn2 (zinc finger 2), Zn3 (zinc finger 3), and UIM (ubiquitin-interacting motif). Numbers indicate the motifs amino-acid positions.

(D) Top: recruitment kinetics of GFP-RNF114-WT or individual GFP-RNF114 deletion constructs (as in C). Bottom: representative confocal images. Scale bars, 5 μm.

(E) Top: recruitment kinetics of GFP-RNF114-WT or GFP-RNF114-C176A (as in C). Bottom: representative confocal images. Scale bars, 5 μm.

(F) Dot blot of recombinant RNF114 and deletion constructs.

(G) Immunoblotting images of WT U2OS cells transfected with indicated plasmids, H_2O_2 treated and subjected to anti-GFP immunoprecipitation. Bound proteins were immunoblotted and stained with the indicated antibodies. Data in (D) and (E) are shown as mean ± SEM from a representative of 5 independent experiments.

accumulation of RNF168, the writer of H2AK13/15Ub (Figure 7L). Altogether, these results are consistent with the role of RNF114 in the DDR and particularly in the NHEJ pathway. Direct assessments of RNF114 contribution to NHEJ with U2OS-EJ5 reporter cells[52] revealed that siRNF114 decreases the frequency of NHEJ events, similar to XRCC4, a core factor required for NHEJ (Figure 7M).

These results shed new light on HPF1/PARP1 signaling by linking the mono-ADPr effector RNF114 to telomere maintenance and DNA repair.

CellPress
OPEN ACCESS

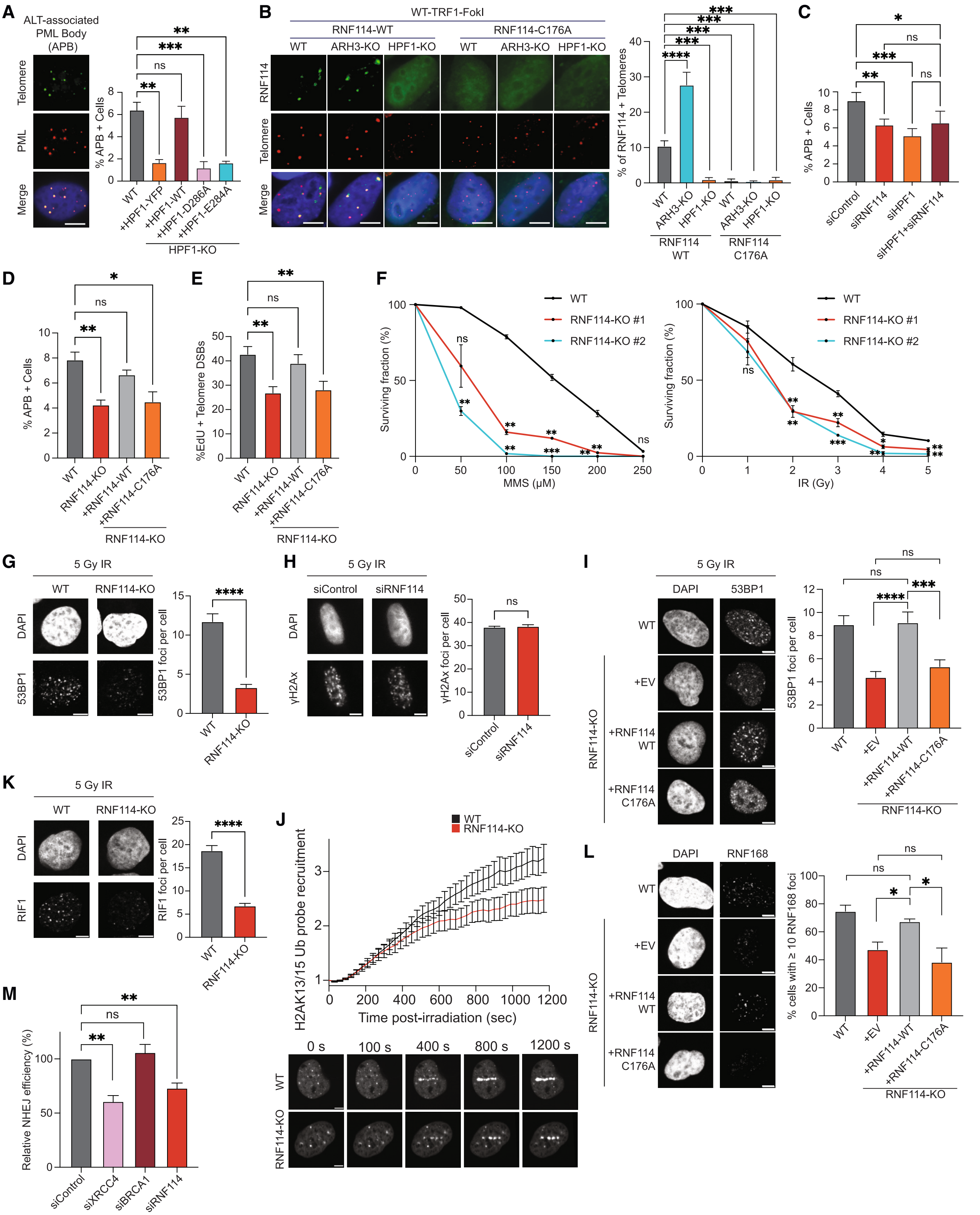

(legend on next page)

DISCUSSION

Although recently introduced tools have significantly advanced ADPr research,[15,19,22,25] our ability to study many forms of this elusive PTM still lags far behind other important PTMs, such as phosphorylation and ubiquitination, for which many mature tools have been made available over the course of several decades. To bridge this technical gap, in this work, we have applied the SpyTag technology[23,24] and affinity maturation to the recombinant antibodies generated with Ser-ADPr-ribosylated peptides according to our phospho-guided enzymatic approach.[15] This has advanced detection of ADPr at multiple levels, creating a blueprint for the future development of antibodies for the sensitive and versatile detection of various elusive targets. The expandable family of formats thus available for every generated recombinant antibody (Figure 1A) may even help ADPr "leapfrog" other PTMs, which rely primarily on non-recombinant antibodies, such as rabbit IgG. As illustrated here for our mono-ADPr antibodies, this system allows straightforward creation of fluorescent probes for live-cell imaging (Figure 2). Importantly, the ongoing development of site-specific ADPr antibodies[15] will soon enable us to track the dynamics of histone and other serine mono-ADPr marks in living cells. This prospect is particularly appealing, considering the distinct dynamics of different serine mono-ADPr targets on DNA damage, as shown for PARP1 and H3.[15] Furthermore, the high sensitivity that we achieve by combining a bivalent HRP-coupled Fab format with affinity maturation extends the reach of these antibodies to the investigation of low-level mono-ADPr events in various signaling pathways regulated by many known mono-ADP-ribosyltransferases.[21]

Limitations of the study

The SpyTag technology[23] has the fundamental limitation that it cannot be applied to non-recombinant antibodies, such as the poly-ADPr antibody clone 10H, and the SpyTag needs be added to existing recombinant reagents by cloning. Although an increasing number of SpyTagged anti-ADPr antibodies and SpyCatcher formats are becoming available, in most cases, the users must couple their own SpyTagged antibody to SpyCatcher reagent following a simple 1 h protocol.[24]

With the SpyTag-based antibodies described here, we show that serine mono-ADPr is the second wave of PARP1 signaling in the context of DNA damage. Its immediate formation and large size make poly-ADPr one of the earliest signals produced during the DDR, and it constitutes a potent "emergency" trigger of DNA repair initiation,[53] but its toxicity[14] makes it unsuitable as an enduring signal. If poly-ADPr were its only signal, the reach of PARP1 signaling would be severely limited by the transient nature of poly-ADPr. Instead, we propose that serine mono-ADPr extends the reach of PARP1 signaling in the form of a second, enduring PTM with which biological processes are regulated over an extended period of time. The persistence of mono-ADPr and transience of poly-ADPr explain the recent puzzling observations that mono-ADPr is more abundant than poly-ADPr in cells upon DNA damage.[15,16] Dynamic modulation of the PARP1/HPF1 ratio in the chromatin milieu constitutes the molecular basis of the dual activity of PARP1 as a poly-ADPr and mono-ADPr transferase and acts as a cellular mechanism to regulate the levels of chromatin mono-ADPr (Figure 3). By identifying the readers of chromatin mono-ADPr, we illustrate how such a two-speed signaling pathway operates in the recruitment of proteins to the sites of DNA damage. Although the recruitment of poly-ADPr readers is immediate and largely temporary (Figures 2 and 5L),[31] the assembly of a mono-ADPr reader, exemplified here by RNF114, is progressive and enduring (Figure 5). Chromatin retention of mono-ADPr readers also occurs in the absence of exogenous DNA damage, as we have shown for cells lacking ARH3. Although the toxicity of high poly-ADPr levels has been suggested as the cause of neurodegeneration in patients with ARH3 deficiency[14] in agreement with the current model of mono-ADPr as a primer for poly-ADPr,[12,13] toxic accumulation of mono-ADPr readers on chromatin might constitute a key factor underlying this inherited neurodegenerative disorder.[26,54–56]

Figure 7. RNF114 modulates the alternative lengthening of telomeres pathway and the DNA damage response

(A) IF images (left) and quantified ABPs (right) in WT and HPF1-KO U2OS cells transfected with indicated plasmids.

(B) Left: representative images of WT, ARH3-KO, and HPF1-KO U2OS cells co-transfected with indicated plasmids. Right: quantification of RNF114 positive telomeres (%). See also Figure S7.

(C) Quantification of APBs in WT U2OS cells transfected with siRNA for control (siControl), HPF1 (siHPF1), RNF114 (siRNF114), or HPF1 + siRNF114. See also Figure S7E.

(D) Quantified APBs in WT and RNF114-KO U2OS cells complemented with GFP-RNF114-WT or GFP-RNF114-C176A.

(E) Quantified relative amounts of DNA synthesis occurring at damaged telomeres (%Edu + telomeres).

(F) Clonogenic cell survival assay of WT and RNF114-KO U2OS cells.

(G) Representative IF images (left) and quantified 53BP1 foci (right) in IR-treated WT or RNF114-KO U2OS cells.

(H) IF images (left) and quantified γH2Ax foci (right) in IR-treated WT HeLa cells, transfected with siControl or siRNF114.

(I) Representative IF images (left) and quantified 53BP1 foci (right) in IR-treated WT, or RNF114-KO U2OS cells stably complemented with GFP-EV, GFP-RNF114-WT, or GFP-RNF114-C176A.

(J) Top: recruitment kinetics of GFP-tagged H2AK13/15Ub probe in WT (black) or ARH3-KO (red) U2OS cells. Bottom: representative confocal images.

(K) Representative IF images (left) and quantified RIF1 foci (right) in IR-treated WT or RNF114-KO U2OS cells. See also Figure S7.

(L) Representative IF images (left) and quantification (right) of RNF168 foci in WT, or RNF114-KO U2OS cells stably complemented with GFP-EV, GFP-RNF114-WT, or GFP-RNF114-C176A treated with 5 Gy IR for 1 h, fixed with PFA and stained with the indicated antibody. The mean ± SEM from 100 cells from a representative of 3 independent experiments is shown.

(M) Quantified relative NHEJ efficiency. Quantification of GFP-positive U2OS-EJ5 cells (relative NHEJ efficiency) after transfection with I-SceI and the indicated siRNAs. Data were normalized to siControl set to 100%.

Data in (A)–(M) are shown as mean ± SEM from 3 to 4 independent experiments. ****p value < 0.0001; ***p value < 0.001; **p value < 0.01; *p value < 0.05; ns, not significant (two-tailed Student's t test). (G–L) Scale bars, 5 μm.

CellPress
OPEN ACCESS

Our work provides the conceptual and technological framework for further dissecting mono-ADPr signaling pathways. Further progress may include the discovery that additional cellular processes are mediated by serine mono-ADPr. Beyond basic research, we anticipate that these new tools and principles will aid the ongoing development of PARP inhibitors that specifically target the composite active sites of the serine mono-ADPr writer HPF1/PARP1.[57] We hope that our findings of serine mono-ADPr as a distinct signal shaping a second wave of PARP1 signaling will trigger a broad reinterpretation, enabled by our modular antibodies, of the scope of the HPF1/PARP1 signaling complex by suggesting a further level of control to the DDR. We propose serine mono-ADPr as a general mechanism by which PARP1 regulate various biological processes with implications for development of clinical inhibitors.

STAR★METHODS

Detailed methods are provided in the online version of this paper and include the following:

- KEY RESOURCES TABLE
- RESOURCE AVAILABILITY
  - Lead contact
  - Materials availability
  - Data and code availability
- EXPERIMENTAL MODEL AND SUBJECT DETAILS
  - Cell culture, SILAC labeling, and drug treatments
  - Plasmids
- METHOD DETAILS
  - Transfections and siRNA treatments
  - Chromatin extraction
  - Acetone precipitation of proteins
  - Recombinant protein production
  - Preparation of nuclear extract for peptide and nucleosome immunoprecipitation
  - Peptide and nucleosome immunoprecipitation
  - Co-immunoprecipitation
  - Histone purification
  - Partial FASP
  - Mono-ADPr immunoprecipitation of purified untreated histones
  - Protein digestion and cleanup for mass spectrometry
  - LC-MS/MS data acquisition
  - *In vitro* HPF1/PARP1 ADP-ribosylation assay
  - Detection of ADP-ribosylated genomic DNA by dot blot assay
  - Recombinant ARH3/DarG treatment of ADPr-gDNA
  - Colony formation assay
  - NHEJ reporter assay
  - Affinity maturation, determination of antibody affinity, and SpyTag coupling
  - Generation of H3S10ADPr nucleosomes via native chemical ligation
  - Indirect enzyme-linked immunosorbent assay (ELISA)
  - Competition ELISA
  - SDS cell lysate preparation
  - Immunoblotting
  - Immunoblotting with on-membrane recombinant proteins treatment
  - Direct immunofluorescence (IF)
  - Antibody labeling
  - Antibody loading
  - Protein recruitment kinetics at sites of laser irradiation
  - Fluorescence correlation spectroscopy
- QUANTIFICATION AND STATISTICAL ANALYSIS
  - Bioinformatic analysis of mass spectrometry samples
  - Statistics on microscopy data
- ADDITIONAL RESOURCES
  - Detailed protocol

SUPPLEMENTAL INFORMATION

Supplemental information can be found online at https://doi.org/10.1016/j.molcel.2023.03.027.

ACKNOWLEDGMENTS

We thank Ilian Atanassov and Xinping Li (MPI-AGE Proteomics Facility), the MPI-AGE FACS/Imaging Facility, and Steffen Lawo (MPI-AGE CRISPR Facility) for help with proteomic analyses, immunofluorescence experiments, and the validation of RNF114-KO cells, respectively. We thank Domagoj Baretic and Evgeniia Prokhorova for providing recombinant RNF114, ALC1, and APLF. Matic laboratory was funded by the European Research Council (ERC) under the European Union's Horizon 2020 research and innovation programme (consolidator grant nbPTMs, agreement No. 864117 to I.M.), DFG (CECAD; grant EXC229 to I.M.), Marie Sklodowska-Curie program (grant 657501 to J.J.B.), and EMBO-YIP (to I.M.). We thank the Microscopy-Rennes-Imaging Center (BIOSIT, Université Rennes 1), members of France-BioImaging supported by the French National Research Agency (ANR-10-INBS-04), for providing access to imaging, and S. Dutertre and X. Pinson for technical assistance. S.H.'s group received financial support from the Agence Nationale de la Recherche (PRC-2018 REPAIRCHROM), the Institut National du Cancer (PLBIO-2019), and the Institut Universitaire de France. R.S. is supported by the Fondation ARC (PDF20181208405). Timinszky laboratory is supported by the Hungarian Academy of Sciences (LP2017-11/2017) and the National Research Development and Innovation Office (K128239 and K143248). O'Sullivan laboratory is funded by NCI (R01CA262316 and R01CA207209). I.A. is funded by the Wellcome Trust (grants 101794 and 210634), the Ovarian Cancer Research Alliance (813369), BBSRC (BB/R007195/1), and CRUK (C35050/A22284).

AUTHOR CONTRIBUTIONS

I.M. conceived the project. I.M., S.H., and E.J.L. designed research. H.D., E.J.L., and J.J.B. designed the phage-display strategy, and selected and validated the antibodies and the SpyTag formats. E.J.L., J.J.B., and T.C. performed proteomics analyses. H.D. established the bead-loading/Fab system. E.J.L., H.D., C.L., R.S., E.P.J., C.M., and A.R.W. performed immunofluorescence and live-cell imaging experiments. A.K. generated the RNF114 KO cell line. A.R.W., M.L.L., and R.J.O. performed telomere-related experiments. R.F.-B. and G.T. performed the NHEJ reporter assay. M.S. and I.A. provided recombinant proteins and performed the experiments with ADP-ribosylated gDNA. E.J.L., C.L., A.P., C.M., and H.D. performed immunoprecipitation and immunoblotting experiments. S.H. performed the FCS experiments. I.M. and E.J.L. prepared the manuscript with input from all authors.

DECLARATION OF INTERESTS

E.J.L., H.D., J.J.B., T.C., and I.M. declare the following competing financial interests: Max-Planck-Innovation, the technology transfer center of the Max Planck Society, has licensed the antibodies AbD33204, AbD33205, AbD33644, AbD34251, AbD33641, and AbD43647 to Bio-Rad Laboratories.

INCLUSION AND DIVERSITY

We support inclusive, diverse, and equitable conduct of research. One or more of the authors of this paper self-identifies as a member of the LGBTQIA+ community.

Received: October 10, 2022
Revised: February 14, 2023
Accepted: March 27, 2023
Published: April 27, 2023

REFERENCES

1. Gupte, R., Liu, Z., and Kraus, W.L. (2017). PARPs and ADP-ribosylation: recent advances linking molecular functions to biological outcomes. Genes Dev. *31*, 101–126. https://doi.org/10.1101/gad.291518.116.
2. Cohen, M.S., and Chang, P. (2018). Insights into the biogenesis, function, and regulation of ADP-ribosylation. Nat. Chem. Biol. *14*, 236–243. https://doi.org/10.1038/Nchembio.2568.
3. Gibson, B.A., Zhang, Y., Jiang, H., Hussey, K.M., Shrimp, J.H., Lin, H., Schwede, F., Yu, Y., and Kraus, W.L. (2016). Chemical genetic discovery of PARP targets reveals a role for PARP-1 in transcription elongation. Science *353*, 45–50. https://doi.org/10.1126/science.aaf7865.
4. Huang, D., and Kraus, W.L. (2022). The expanding universe of PARP1-mediated molecular and therapeutic mechanisms. Mol. Cell *82*, 2315–2334. https://doi.org/10.1016/j.molcel.2022.02.021.
5. Leidecker, O., Bonfiglio, J.J., Colby, T., Zhang, Q., Atanassov, I., Zaja, R., Palazzo, L., Stockum, A., Ahel, I., and Matic, I. (2016). Serine is a new target residue for endogenous ADP-ribosylation on histones. Nat. Chem. Biol. *12*, 998–1000. https://doi.org/10.1038/nchembio.2180.
6. Bonfiglio, J.J., Fontana, P., Zhang, Q., Colby, T., Gibbs-Seymour, I., Atanassov, I., Bartlett, E., Zaja, R., Ahel, I., and Matic, I. (2017). Serine ADP-ribosylation depends on HPF1. Mol. Cell *65*, 932–940.e6. https://doi.org/10.1016/j.molcel.2017.01.003.
7. Larsen, S.C., Hendriks, I.A., Lyon, D., Jensen, L.J., and Nielsen, M.L. (2018). Systems-wide analysis of serine ADP-ribosylation reveals widespread occurrence and site-specific overlap with phosphorylation. Cell Rep. *24*, 2493–2505.e4. https://doi.org/10.1016/j.celrep.2018.07.083.
8. Palazzo, L., Leidecker, O., Prokhorova, E., Dauben, H., Matic, I., and Ahel, I. (2018). Serine is the major residue for ADP-ribosylation upon DNA damage. eLife *7*, e34334. https://doi.org/10.7554/eLife.34334.
9. Abplanalp, J., Leutert, M., Frugier, E., Nowak, K., Feurer, R., Kato, J., Kistemaker, H.V.A., Filippov, D.V., Moss, J., Caflisch, A., et al. (2017). Proteomic analyses identify ARH3 as a serine mono-ADP-ribosylhydrolase. Nat. Commun. *8*, 2055. https://doi.org/10.1038/s41467-017-02253-1.
10. Gibbs-Seymour, I., Fontana, P., Rack, J.G.M., and Ahel, I. (2016). HPF1/C4orf27 is a PARP-1-Interacting protein that regulates PARP-1 ADP-ribosylation activity. Mol. Cell *62*, 432–442. https://doi.org/10.1016/j.molcel.2016.03.008.
11. Fontana, P., Bonfiglio, J.J., Palazzo, L., Bartlett, E., Matic, I., and Ahel, I. (2017). Serine ADP-ribosylation reversal by the hydrolase ARH3. eLife *6*, e28533. https://doi.org/10.7554/eLife.28533.
12. Suskiewicz, M.J., Zobel, F., Ogden, T.E.H., Fontana, P., Ariza, A., Yang, J.C., Zhu, K., Bracken, L., Hawthorne, W.J., Ahel, D., et al. (2020). HPF1 completes the PARP active site for DNA damage-induced ADP-ribosylation. Nature *579*, 598–602. https://doi.org/10.1038/s41586-020-2013-6.
13. Langelier, M.F., Billur, R., Sverzhinsky, A., Black, B.E., and Pascal, J.M. (2021). HPF1 dynamically controls the PARP1/2 balance between initiating and elongating ADP-ribose modifications. Nat. Commun. *12*, 6675. https://doi.org/10.1038/s41467-021-27043-8.
14. Prokhorova, E., Agnew, T., Wondisford, A.R., Tellier, M., Kaminski, N., Beijer, D., Holder, J., Groslambert, J., Suskiewicz, M.J., Zhu, K., et al. (2021). Unrestrained poly-ADP-ribosylation provides insights into chromatin regulation and human disease. Mol. Cell *81*, 2640–2655.e8. https://doi.org/10.1016/j.molcel.2021.04.028.
15. Bonfiglio, J.J., Leidecker, O., Dauben, H., Longarini, E.J., Colby, T., San Segundo-Acosta, P., Perez, K.A., and Matic, I. (2020). An HPF1/PARP1-based chemical biology strategy for exploring ADP-ribosylation. Cell *183*, 1086–1102.e23. https://doi.org/10.1016/j.cell.2020.09.055.
16. Hendriks, I.A., Buch-Larsen, S.C., Prokhorova, E., Elsborg, J.D., Rebak, A.K.L.F.S., Zhu, K., Ahel, D., Lukas, C., Ahel, I., and Nielsen, M.L. (2021). The regulatory landscape of the human HPF1- and ARH3-dependent ADP-ribosylome. Nat. Commun. *12*, 5893. https://doi.org/10.1038/s41467-021-26172-4.
17. Hananya, N., Daley, S.K., Bagert, J.D., and Muir, T.W. (2021). Synthesis of ADP-ribosylated histones reveals site-specific impacts on chromatin structure and function. J. Am. Chem. Soc. *143*, 10847–10852. https://doi.org/10.1021/jacs.1c05429.
18. Tashiro, K., Mohapatra, J., Brautigam, C.A., and Liszczak, G. (2022). A protein semisynthesis-based strategy to investigate the functional impact of linker histone serine ADP-ribosylation. ACS Chem. Biol. *17*, 810–815. https://doi.org/10.1021/acschembio.2c00091.
19. Gibson, B.A., Conrad, L.B., Huang, D., and Kraus, W.L. (2017). Generation and characterization of recombinant antibody-like ADP-ribose binding proteins. Biochemistry *56*, 6305–6316. https://doi.org/10.1021/acs.biochem.7b00670.
20. Liu, Q., van der Marel, G.A., and Filippov, D.V. (2019). Chemical ADP-ribosylation: mono-ADPr-peptides and oligo-ADP-ribose. Org. Biomol. Chem. *17*, 5460–5474. https://doi.org/10.1039/c9ob00501c.
21. Vyas, S., Matic, I., Uchima, L., Rood, J., Zaja, R., Hay, R.T., Ahel, I., and Chang, P. (2014). Family-wide analysis of poly(ADP-ribose) polymerase activity. Nat. Commun. *5*, 4426. https://doi.org/10.1038/ncomms5426.
22. Nowak, K., Rosenthal, F., Karlberg, T., Bütepage, M., Thorsell, A.G., Dreier, B., Grossmann, J., Sobek, J., Imhof, R., Lüscher, B., et al. (2020). Engineering Af1521 improves ADP-ribose binding and identification of ADP-ribosylated proteins. Nat. Commun. *11*, 5199. https://doi.org/10.1038/s41467-020-18981-w.
23. Zakeri, B., Fierer, J.O., Celik, E., Chittock, E.C., Schwarz-Linek, U., Moy, V.T., and Howarth, M. (2012). Peptide tag forming a rapid covalent bond to a protein, through engineering a bacterial adhesin. Proc. Natl. Acad. Sci. USA *109*, E690–E697. https://doi.org/10.1073/pnas.1115485109.
24. Hentrich, C., Kellmann, S.J., Putyrski, M., Cavada, M., Hanuschka, H., Knappik, A., and Ylera, F. (2021). Periplasmic expression of SpyTagged antibody fragments enables rapid modular antibody assembly. Cell Chem. Biol. *28*, 813–824.e6. https://doi.org/10.1016/j.chembiol.2021.01.011.
25. Challa, S., Ryu, K.W., Whitaker, A.L., Abshier, J.C., Camacho, C.V., and Kraus, W.L. (2022). Development and characterization of new tools for detecting poly(ADP-ribose) in vitro and in vivo. eLife *11*, e72464. https://doi.org/10.7554/eLife.72464.
26. Hanzlikova, H., Prokhorova, E., Krejcikova, K., Cihlarova, Z., Kalasova, I., Kubovciak, J., Sachova, J., Hailstone, R., Brazina, J., Ghosh, S., et al. (2020). Pathogenic ARH3 mutations result in ADP-ribose chromatin scars during DNA strand break repair. Nat. Commun. *11*, 3391. https://doi.org/10.1038/s41467-020-17069-9.
27. Cho, N.W., Dilley, R.L., Lampson, M.A., and Greenberg, R.A. (2014). Interchromosomal homology searches drive directional ALT telomere movement and synapsis. Cell *159*, 108–121. https://doi.org/10.1016/j.cell.2014.08.030.
28. Hopp, A.K., Teloni, F., Bisceglie, L., Gondrand, C., Raith, F., Nowak, K., Muskalla, L., Howald, A., Pedrioli, P.G.A., Johnsson, K., et al. (2021). Mitochondrial NAD(+) controls nuclear ARTD1-induced ADP-ribosylation. Mol. Cell *81*, 340–354.e5. https://doi.org/10.1016/j.molcel.2020.12.034.
29. Schuller, M., Butler, R.E., Ariza, A., Tromans-Coia, C., Jankevicius, G., Claridge, T.D.W., Kendall, S.L., Goh, S., Stewart, G.R., and Ahel, I.

CellPress
OPEN ACCESS

(2021). Molecular basis for DarT ADP-ribosylation of a DNA base. Nature *596*, 597–602. https://doi.org/10.1038/s41586-021-03825-4.

30. Hayashi-Takanaka, Y., Yamagata, K., Wakayama, T., Stasevich, T.J., Kainuma, T., Tsurimoto, T., Tachibana, M., Shinkai, Y., Kurumizaka, H., Nozaki, N., et al. (2011). Tracking epigenetic histone modifications in single cells using Fab-based live endogenous modification labeling. Nucleic Acids Res. *39*, 6475–6488. https://doi.org/10.1093/nar/gkr343.
31. Smith, R., Lebeaupin, T., Juhász, S., Chapuis, C., D'Augustin, O., Dutertre, S., Burkovics, P., Biertümpfel, C., Timinszky, G., and Huet, S. (2019). Poly(ADP-ribose)-dependent chromatin unfolding facilitates the association of DNA-binding proteins with DNA at sites of damage. Nucleic Acids Res. *47*, 11250–11267. https://doi.org/10.1093/nar/gkz820.
32. Smith, R., Zentout, S., Chapuis, C., Timinszky, G., and Huet, S. (2021). HPF1-dependent histone ADP-ribosylation triggers chromatin relaxation to promote the recruitment of repair factors at sites of DNA damage. https://doi.org/10.1101/2021.08.27.457930.
33. Koczor, C.A., Saville, K.M., Andrews, J.F., Clark, J., Fang, Q., Li, J., Al-Rahahleh, R.Q., Ibrahim, M., McClellan, S., Makarov, M.V., et al. (2021). Temporal dynamics of base excision/single-strand break repair protein complex assembly/disassembly are modulated by the PARP/NAD(+)/SIRT6 axis. Cell Rep. *37*, 109917. https://doi.org/10.1016/j.celrep.2021.109917.
34. Jankevicius, G., Hassler, M., Golia, B., Rybin, V., Zacharias, M., Timinszky, G., and Ladurner, A.G. (2013). A family of macrodomain proteins reverses cellular mono-ADP-ribosylation. Nat. Struct. Mol. Biol. *20*, 508–514. https://doi.org/10.1038/nsmb.2523.
35. Mohapatra, J., Tashiro, K., Beckner, R.L., Sierra, J., Kilgore, J.A., Williams, N.S., and Liszczak, G. (2021). Serine ADP-ribosylation marks nucleosomes for ALC1-dependent chromatin remodeling. eLife *10*, e71502. https://doi.org/10.7554/eLife.71502.
36. Yang, C.S., Jividen, K., Spencer, A., Dworak, N., Ni, L., Oostdyk, L.T., Chatterjee, M., Kuśmider, B., Reon, B., Parlak, M., et al. (2017). Ubiquitin modification by the E3 ligase/ADP-ribosyltransferase Dtx3L/Parp9. Mol. Cell *66*, 503–516.e5. https://doi.org/10.1016/j.molcel.2017.04.028.
37. Zhu, K., Suskiewicz, M.J., Hloušek-Kasun, A., Meudal, H., Mikoč, A., Aucagne, V., Ahel, D., and Ahel, I. (2022). DELTEX E3 ligases ubiquitylate ADP-ribosyl modification on protein substrates. Sci. Adv. *8*, eadd4253. https://doi.org/10.1126/sciadv.add4253.
38. Ahel, D., Horejsí, Z., Wiechens, N., Polo, S.E., Garcia-Wilson, E., Ahel, I., Flynn, H., Skehel, M., West, S.C., Jackson, S.P., et al. (2009). Poly(ADP-ribose)-dependent regulation of DNA repair by the chromatin remodeling enzyme ALC1. Science *325*, 1240–1243. https://doi.org/10.1126/science.1177321.
39. Dasovich, M., Beckett, M.Q., Bailey, S., Ong, S.E., Greenberg, M.M., and Leung, A.K.L. (2021). Identifying poly(ADP-ribose)-binding proteins with photoaffinity-based proteomics. J. Am. Chem. Soc. *143*, 3037–3042. https://doi.org/10.1021/jacs.0c12246.
40. Kliza, K.W., Liu, Q., Roosenboom, L.W.M., Jansen, P.W.T.C., Filippov, D.V., and Vermeulen, M. (2021). Reading ADP-ribosylation signaling using chemical biology and interaction proteomics. Mol. Cell *81*, 4552–4567.e8. https://doi.org/10.1016/j.molcel.2021.08.037.
41. Mao, Z., Hine, C., Tian, X., Van Meter, M., Au, M., Vaidya, A., Seluanov, A., and Gorbunova, V. (2011). SIRT6 promotes DNA repair under stress by activating PARP1. Science *332*, 1443–1446. https://doi.org/10.1126/science.1202723.
42. Mostoslavsky, R., Chua, K.F., Lombard, D.B., Pang, W.W., Fischer, M.R., Gellon, L., Liu, P., Mostoslavsky, G., Franco, S., Murphy, M.M., et al. (2006). Genomic instability and aging-like phenotype in the absence of mammalian SIRT6. Cell *124*, 315–329. https://doi.org/10.1016/j.cell.2005.11.044.
43. Han, J., Kim, Y.L., Lee, K.W., Her, N.G., Ha, T.K., Yoon, S., Jeong, S.I., Lee, J.H., Kang, M.J., Lee, M.G., et al. (2013). ZNF313 is a novel cell cycle activator with an E3 ligase activity inhibiting cellular senescence by destabilizing p21(WAF1). Cell Death Differ. *20*, 1055–1067. https://doi.org/10.1038/cdd.2013.33.
44. Ahel, I., Ahel, D., Matsusaka, T., Clark, A.J., Pines, J., Boulton, S.J., and West, S.C. (2008). Poly(ADP-ribose)-binding zinc finger motifs in DNA repair/checkpoint proteins. Nature *451*, 81–85. https://doi.org/10.1038/nature06420.
45. Wachsmuth, M., Conrad, C., Bulkescher, J., Koch, B., Mahen, R., Isokane, M., Pepperkok, R., and Ellenberg, J. (2015). High-throughput fluorescence correlation spectroscopy enables analysis of proteome dynamics in living cells. Nat. Biotechnol. *33*, 384–389. https://doi.org/10.1038/nbt.3146.
46. Zhao, Y.H., Liang, X., Wei, L., Liu, Y., Liu, J., Feng, H., Zheng, F., Wang, Y., Ma, H.J., and Wu, J.X. (2021). RNF114 suppresses metastasis through regulation of PARP10 in cervical cancer cells. Cancer Commun. (Lond) *41*, 187–191. https://doi.org/10.1002/cac2.12132.
47. Gosti, F., Bertauche, N., Vartanian, N., and Giraudat, J. (1995). Abscisic acid-dependent and -independent regulation of gene expression by progressive drought in Arabidopsis thaliana. Mol. Gen. Genet. *246*, 10–18. https://doi.org/10.1007/BF00290128.
48. Zhang, J.M., Yadav, T., Ouyang, J., Lan, L., and Zou, L. (2019). Alternative lengthening of telomeres through two distinct break-induced replication pathways. Cell Rep. *26*, 955–968.e3. https://doi.org/10.1016/j.celrep.2018.12.102.
49. Panier, S., and Boulton, S.J. (2014). Double-strand break repair: 53BP1 comes into focus. Nat. Rev. Mol. Cell Biol. *15*, 7–18. https://doi.org/10.1038/nrm3719.
50. Caron, M.C., Sharma, A.K., O'Sullivan, J., Myler, L.R., Ferreira, M.T., Rodrigue, A., Coulombe, Y., Ethier, C., Gagné, J.P., Langelier, M.F., et al. (2019). Poly(ADP-ribose) polymerase-1 antagonizes DNA resection at double-strand breaks. Nat. Commun. *10*, 2954. https://doi.org/10.1038/s41467-019-10741-9.
51. Dos Santos Passos, C., Choi, Y.S., Snow, C.D., Yao, T., and Cohen, R.E. (2021). Design of genetically encoded sensors to detect nucleosome ubiquitination in live cells. J. Cell Biol. *220*, e201911130. https://doi.org/10.1083/jcb.201911130.
52. Gunn, A., Bennardo, N., Cheng, A., and Stark, J.M. (2011). Correct end use during end joining of multiple chromosomal double strand breaks is influenced by repair protein RAD50, DNA-dependent protein kinase DNA-PKcs, and transcription context. J. Biol. Chem. *286*, 42470–42482. https://doi.org/10.1074/jbc.M111.309252.
53. Liu, C., Vyas, A., Kassab, M.A., Singh, A.K., and Yu, X. (2017). The role of poly ADP-ribosylation in the first wave of DNA damage response. Nucleic Acids Res. *45*, 8129–8141. https://doi.org/10.1093/nar/gkx565.
54. Danhauser, K., Alhaddad, B., Makowski, C., Piekutowska-Abramczuk, D., Syrbe, S., Gomez-Ospina, N., Manning, M.A., Kostera-Pruszczyk, A., Krahn-Peper, C., Berutti, R., et al. (2018). Bi-allelic ADPRHL2 mutations cause neurodegeneration with developmental delay, ataxia, and axonal neuropathy. Am. J. Hum. Genet. *103*, 817–825. https://doi.org/10.1016/j.ajhg.2018.10.005.
55. Ghosh, S.G., Becker, K., Huang, H., Dixon-Salazar, T., Chai, G., Salpietro, V., Al-Gazali, L., Waisfisz, Q., Wang, H., Vaux, K.K., et al. (2018). Biallelic mutations in ADPRHL2, encoding ADP-ribosylhydrolase 3, lead to a degenerative pediatric stress-induced epileptic ataxia syndrome. Am. J. Hum. Genet. *103*, 431–439. https://doi.org/10.1016/j.ajhg.2018.07.010.
56. Beijer, D., Agnew, T., Rack, J.G.M., Prokhorova, E., Deconinck, T., Ceulemans, B., Peric, S., Milic Rasic, V., De Jonghe, P., Ahel, I., et al. (2021). Biallelic ADPRHL2 mutations in complex neuropathy affect ADP ribosylation and DNA damage response. Life Sci. Alliance *4*, 4. https://doi.org/10.26508/lsa.202101057.
57. Rudolph, J., Jung, K., and Luger, K. (2022). Inhibitors of PARP: number crunching and structure gazing. Proc. Natl. Acad. Sci. USA *119*, e2121979119. https://doi.org/10.1073/pnas.2121979119.
58. Langelier, M.F., Planck, J.L., Servent, K.M., and Pascal, J.M. (2011). Purification of human PARP-1 and PARP-1 domains from Escherichia

coli for structural and biochemical analysis. Methods Mol. Biol. *780*, 209–226. https://doi.org/10.1007/978-1-61779-270-0_13.

59. Lambrecht, M.J., Brichacek, M., Barkauskaite, E., Ariza, A., Ahel, I., and Hergenrother, P.J. (2015). Synthesis of dimeric ADP-ribose and its structure with human poly(ADP-ribose) glycohydrolase. J. Am. Chem. Soc. *137*, 3558–3564. https://doi.org/10.1021/ja512528p.

60. Kernstock, S., Koch-Nolte, F., Mueller-Dieckmann, J., Weiss, M.S., and Mueller-Dieckmann, C. (2006). Cloning, expression, purification, crystallization and preliminary X-ray diffraction analysis of human ARH3, the first eukaryotic protein-ADP-ribosylhydrolase. Acta Crystallogr. Sect. F Struct. Biol. Cryst. Commun. *62*, 224–227. https://doi.org/10.1107/S1744309106003435.

61. Cox, J., and Mann, M. (2008). MaxQuant enables high peptide identification rates, individualized p.p.b.-range mass accuracies and proteome-wide protein quantification. Nat. Biotechnol. *26*, 1367–1372. https://doi.org/10.1038/nbt.1511.

62. Tyanova, S., Temu, T., Sinitcyn, P., Carlson, A., Hein, M.Y., Geiger, T., Mann, M., and Cox, J. (2016). The Perseus computational platform for comprehensive analysis of (prote)omics data. Nat. Methods *13*, 731–740. https://doi.org/10.1038/nmeth.3901.

63. Labun, K., Montague, T.G., Krause, M., Torres Cleuren, Y.N., Tjeldnes, H., and Valen, E. (2019). CHOPCHOP v3: expanding the CRISPR web toolbox beyond genome editing. Nucleic Acids Res. *47*, W171–W174. https://doi.org/10.1093/nar/gkz365.

64. Conant, D., Hsiau, T., Rossi, N., Oki, J., Maures, T., Waite, K., Yang, J., Joshi, S., Kelso, R., Holden, K., et al. (2022). Inference of CRISPR edits from sanger trace data. CRISPR J. *5*, 123–130. https://doi.org/10.1089/crispr.2021.0113.

65. Ong, S.E., Blagoev, B., Kratchmarova, I., Kristensen, D.B., Steen, H., Pandey, A., and Mann, M. (2002). Stable isotope labeling by amino acids in cell culture, SILAC, as a simple and accurate approach to expression proteomics. Mol. Cell. Proteomics *1*, 376–386. https://doi.org/10.1074/mcp.M200025-MCP200.

66. Bartke, T., Vermeulen, M., Xhemalce, B., Robson, S.C., Mann, M., and Kouzarides, T. (2010). Nucleosome-interacting proteins regulated by DNA and histone methylation. Cell *143*, 470–484. https://doi.org/10.1016/j.cell.2010.10.012.

67. Richardson, C., Moynahan, M.E., and Jasin, M. (1998). Double-strand break repair by interchromosomal recombination: suppression of chromosomal translocations. Genes Dev. *12*, 3831–3842. https://doi.org/10.1101/gad.12.24.3831.

68. Prassler, J., Thiel, S., Pracht, C., Polzer, A., Peters, S., Bauer, M., Nörenberg, S., Stark, Y., Kölln, J., Popp, A., et al. (2011). HuCAL platinum, a synthetic Fab library optimized for sequence diversity and superior performance in mammalian expression systems. J. Mol. Biol. *413*, 261–278. https://doi.org/10.1016/j.jmb.2011.08.012.

69. Steidl, S., Ratsch, O., Brocks, B., Dürr, M., and Thomassen-Wolf, E. (2008). In vitro affinity maturation of human GM-CSF antibodies by targeted CDR-diversification. Mol. Immunol. *46*, 135–144. https://doi.org/10.1016/j.molimm.2008.07.013.

70. Shah, R.N., Grzybowski, A.T., Cornett, E.M., Johnstone, A.L., Dickson, B.M., Boone, B.A., Cheek, M.A., Cowles, M.W., Maryanski, D., Meiners, M.J., et al. (2018). Examining the roles of H3K4 methylation states with systematically characterized antibodies. Mol. Cell *72*, 162–177.e7. https://doi.org/10.1016/j.molcel.2018.08.015.

71. Cialek, C.A., Galindo, G., Koch, A.L., Saxton, M.N., and Stasevich, T.J. (2021). Bead loading proteins and nucleic acids into adherent human cells. J. Vis. Exp. *62559*. https://doi.org/10.3791/62559.

72. Ritchie, M.E., Phipson, B., Wu, D., Hu, Y.F., Law, C.W., Shi, W., and Smyth, G.K. (2015). limma powers differential expression analyses for RNA-sequencing and microarray studies. Nucleic Acids Res. *43*, e47. https://doi.org/10.1093/nar/gkv007.

73. Dennis, G., Jr., Sherman, B.T., Hosack, D.A., Yang, J., Gao, W., Lane, H.C., and Lempicki, R.A. (2003). DAVID: database for annotation, visualization, and integrated discovery. Genome Biol. *4*, P3.

STAR★METHODS

KEY RESOURCES TABLE

REAGENT or RESOURCE	SOURCE	IDENTIFIER
Antibodies		
Anti-mono-ADPr antibody	Bio-Rad	AbD43647
Anti-pan-ADPr antibody	Bio-Rad	AbD33641
Anti-protein mono-ADPr antibody	Bio-Rad	AbD33205
Anti-mono-ADPr antibody	Bio-Rad	AbD33204
Anti-PARP1-S499ADPr	Bio-Rad	AbD34251
Anti-H3-S10/28ADPr	Bio-Rad	AbD33644
Anti-Mouse Alexa-Fluor 594-conjugated goat secondary	Invitrogen	Cat# A11005; RRID:AB_2534073
Anti-HPF1 polyclonal antibody	Sigma-Aldrich	Cat# HPA043467; RRID:AB_10793949
Anti-human IgG F(ab') HRP-conjugated goat secondary	Bio-Rad	Cat# STAR126; RRID:AB_1605086
Anti-PARP1 polyclonal antibody	Abcam	Cat# ab6079; RRID:AB_305284
Anti-GFP Living Colors® A.v. Monoclonal Antibody (JL-8)	Takara	Cat# 632381; RRID:AB_2313808
Anti-RNF168 antibody	Novus Biologicals	Cat# AF7217; RRID:AB_10971653
Anti-RIF1 antibody	Abcam	Cat# ab229656
Anti-phospho-Histone H2A.X (Ser139) antibody	Millipore	Cat# 05-636; RRID:AB_309864
Anti-53BP1 antibody	BD Transduction Laboratories	Cat# 612523; RRID:AB_399824
Anti-DTX3L (D5F2J) antibody	Cell Signaling Technology	Cat# 14795S; RRID:AB_2798615
Anti-RNF114 antibody	Sigma-Aldrich	Cat# HPA021184; RRID:AB_1859185
Anti-unmodified-Histone H3 (Lys9) antibody, clone 9B1-2G6, Mouse monoclonal	Sigma-Aldrich	Cat# SAB4200591
Anti-FLAG M2 antibody (HRP)	Sigma-Aldrich	Cat# A8592; RRID:AB_439702
Anti-Streptavidin antibody (HRP)	Cell Signaling Technology	Cat# 3999; RRID:AB_10830897
Anti-GST antibody (HRP)	Abcam	Cat# ab3416; RRID:AB_303783
Anti-mono/poly-ADP0-ribose antibody (E6F6A)	Cell Signaling Technology	Cat# 83732; RRID:AB_2749858
Anti-poly-ADP-ribose binding reagent	Enzo Life Sciences	Cat# ALX-804-220-R100; RRID:AB_2052275
Anti-poly-ADP-ribose binding reagent	Millipore	Cat# MABE1031; RRID:AB_2665467
Anti-PAN-ADP-ribose binding reagent	Millipore	Cat# MABE1016; RRID:AB_2665466
Anti-H3 polyclonal antibody	Cell Signaling Technology	Cat# 9715S; RRID:AB_331563
Anti-Human FITC-conjugated goat secondary	Bio-Rad	Cat# STAR126F; RRID:AB_1102647
Anti-mouse IgG HRP-conjugated secondary	Amersham	Cat# NA931V; RRID:AB_772210
Anti-rabbit IgG HRP-conjugated secondary	Merck	Cat# GENA934-1ML; RRID:AB_2722659
Chemicals, peptides, and recombinant proteins		
Histone H3 (1-21), Biotinylated Ac-ARTKQTARKSTGGKAPRKQLAGG K(Biotin)-Am	AnaSpec	Cat# AS-61702
Histone H3 (1-34) S28ph Ac-ARTKQTARKSTGGKAPRKQL ATKAARKS(ph)APATGG-Am	SB-peptide	N/A
Histone H3(22-44), Biotinylated Ac-ATKAARKSAPATGGVKKPHRYRPGG GK(Biotin)-Am	AnaSpec	Cat# AS-64440-1
Histone H4(1-19), Biotinylated Ac-SGRGKGGKGLGKGGAKRHRGGK(Biotin)-Am	GeneCust	N/A
Recombinant Human PARP1 protein	Langelier et al.[58]	N/A
Recombinant Human PARG protein	Lambrecht et al.[59]	N/A

(Continued on next page)

Continued

REAGENT or RESOURCE	SOURCE	IDENTIFIER
Recombinant Human ARH3 protein	Kernstock et al.[60]	N/A
Recombinant Human HPF1 protein	Gibbs-Seymour et al.[10]	N/A
Recombinant Human RNF114-WT	This manuscript	N/A
Recombinant Human RNF114-ΔZn1	This manuscript	N/A
Recombinant Human RNF114-ΔZn2	This manuscript	N/A
Recombinant Human RNF114-ΔZn3	This manuscript	N/A
Recombinant Human RNF114-C176A	This manuscript	N/A
Recombinant Human APLF protein	Ahel et al.[44]	N/A
Recombinant Human ALC1 protein	Ahel et al.[38]	N/A
Mononucleosomes, Recombinant Human Biotinylated	Epicypher	Cat# 16-0006
Recombinant human histone H3.1	NEB	Cat# M2503S
Recombinant Lambda Protein Phosphatase	NEB	Cat# P0753
Imperial Protein Stain	Thermo Scientific	Cat# 24615
PARG inhibitor (PDD00017273)	Sigma-Aldrich	Cat# SML1781
Olaparib	Cayman Chemical	Cat# 10621
ADP-HPD, Dihydrate, Ammonium Salt	Millipore	Cat# 118415
Sonicated DNA	Biomol	Cat# 54653
NAD^+	Trevigen	Cat# 4684-096-02
Trypsin Gold, Mass Spectrometry Grade	Promega	Cat# V5280
Pierce™ Lys-C Protease, MS Grade	Thermofisher Scientific	Cat# 90051
cOmplete EDTA-free Protease Inhibitor Cocktail	Sigma-Aldrich	Cat# 11873580001
Unlabelled L-lysine (Light)	Sigma-Aldrich	Cat# L8662
Isotopically labeled L-lysine (13C6,15N2)	Sigma-Aldrich	Cat# 608041
Unlabeled L-Arginine (Light)	Sigma-Aldrich	Cat# A8094
Isotopically labeled L-Arginine ($^{13}C_6$, $^{15}N_4$)	Sigma-Aldrich	Cat# 608033
QuantaBlu™ Fluorogenic Peroxidase Substrate Kit	Thermo Scientific	Cat# 15169
ProLong™ Diamond Antifade Mountant	Invitrogen	Cat# P36970
Polyethylenimine (PEI)	Sigma-Aldrich	Cat# 408727
Xfect Transfection Reagent	Takara	Cat# 631318
Lipofectamine 2000	Invitrogen	Cat# 11668019
G-418 solution	Roche	Cat# 4727878001
L-glutathione reduced	Sigma-Aldrich	Cat# G4251
IPTG	Sigma-Aldrich	Cat# I6758
Biotin (terminal)-PAR polymer	Trevigen	Cat# 4336-100-02
Snake Venom Phosphodiesterase I from *Crotalus adamanteus*	Sigma-Aldrich	Cat# P3243-1VL
Adenosine	Sigma-Aldrich	Cat# A9251
Adenosine 5'-monophosphate	Sigma-Aldrich	Cat# A1752
Adenosine 2',5'-diphosphate	Sigma-Aldrich	Cat# A2754
2'-deoxyadenosine 5'-monophosphate	Sigma-Aldrich	Cat# D6375
Adenosine 5'-triphosphate	Sigma-Aldrich	Cat# A2383
Adenosine 5'-diphosphoribose sodium salt	Sigma-Aldrich	Cat# A0752
Guanosine 5'-diphosphate	Sigma-Aldrich	Cat# 51060
Cytidine 5'-diphosphate	Sigma-Aldrich	Cat# C9755
Ibidi U dish 35 mm	Ibidi	Cat# 81158
Ibidi µ-slide 8 well grid-500	Ibidi	Cat# 80806
Microtiter plate 384 well Maxisorp MTP	Thermo Scientific	Cat# 10395991
Methylmethanesulfonate (MMS)	Sigma-Aldrich	Cat# 129925

(*Continued on next page*)

Continued

REAGENT or RESOURCE	SOURCE	IDENTIFIER
Cytiva Amersham Protran 0.2 NC	Amersham	Cat# 10600001
Ponceau S	Sigma-Aldrich	Cat# P7170
DAPI	Sigma-Aldrich	Cat# D9542
Live Cell Imaging Solution	Invitrogen	Cat # A14291DJ
Hoechst 33342	Sigma-Aldrich	Cat# 14533-100MG
MitoTracker™ Red CMXRos	Invitrogen	Cat# M7512
Critical commercial assays		
SuperSignal™ West Atto Ultimate Sensitivity Chemiluminescent Substrate	Thermo Scientific	Cat# A38554
Ni-NTA agarose	Qiagen	Cat# 30250
Glass beads, < 106 μm	Sigma-Aldrich	Cat# G4649
Glutathione Sepharose 4B GST-tagged protein purification resin	Cytiva	Cat# 17-0756-01
Vivacon 500, 10 kDa MWCO	Sartorius	Cat# VN01H02
Dynabeads MyOne C1 streptavidin	Pierce	Cat# 65002
Dynabeads MyOne T1 streptavidin	Pierce	Cat# 65601
Chromotek GFP-trap Magnetic Particles M-270	Chromotek	Cat# gtd
Protein A Agarose Beads	Cell Signaling	Cat# 9863
Pierce™ NeutrAvidin™ Coated Plates, Black, 96-Well	Themo Scientific	Cat# 15117
Guide-it CRISPR/Cas9 System	Takara	Cat# 632601
m-Aminophenylboronic acid–Agarose	Sigma-Aldrich	Cat# A8312
Deposited data		
Mass spectrometry data: Identification of mono-ADPr readers by multilevel chromatin proteomics	This manuscript	ProteomeXchange: PXD037026
Original imaging data	This manuscript	Mendeley Data: https://doi.org/10.17632/fdnscb6pn8.1
Experimental models: Cell lines		
Human: U2OS cells	ATCC	Cat# HTB-96
Human: U2OS ARH3-KO cells	Fontana et al.[11]	N/A
Human: U2OS HPF1-KO cells	Gibbs-Seymour et al.[10]	N/A
Human: U2OS PARP1-KO cells	Gibbs-Seymour et al.[10]	N/A
Human: U2OS RNF114-KO cells	This manuscript	N/A
Human: U2OS RNF114-KO cells complemented with GFP-Empty Vector	This manuscript	N/A
Human: U2OS RNF114-KO cells complemented with GFP-Tagged RNF114-WT	This manuscript	N/A
Human: U2OS RNF114-KO cells complemented with GFP-Tagged RNF114-C176A	This manuscript	N/A
Software and algorithms		
MaxQuant proteomics suite of algorithms (version 1.5.3.17)	Cox and Mann[61]	http://www.coxdocs.org/doku.php?id=maxquant:start
Perseus software	Tyanova et al.[62]	http://www.perseus-framework.org
Prism 9	GraphPad	N/A
ImageJ	NIH	N/A
Other		
Detailed protocol for immunoblotting using the HRP-coupled SpyTag format	This manuscript	Methods S1

RESOURCE AVAILABILITY

Lead contact

Further information and requests for resources and reagents should be directed to and will be fulfilled by the lead contact, Dr. Ivan Matic (imatic@age.mpg.de).

Materials availability

The antibody generated in this study (AbD43647) as well as the previously published antibodies[15] converted to SpyTag formats in this study are available through Bio-Rad Laboratories.

Uncoupled Fab-SpyTag (monovalent format): AbD33641ad (Catalog # TZA024): AbD33205ad (Catalog # TZA021); AbD33204ad (Catalog # TZA019); AbD34251ad (Catalog # TZA022); AbD33644ad (Catalog # TZA023); AbD43647ad (Catalog # TZA020).

Fab-SpyTag coupled to HRP-conjugated BiCatcher2 (bivalent format): AbD43647pap (Catalog # TZA020P). The other antibodies can be obtained in this format via conjugation to BiCatcher2:HRP (Catalog # TZC002P).

Fab-SpyTag coupled to rabbit IgG FcCatchers (IgG-like format): the antibodies can be obtained in this format via conjugation to rbIgG-FcSpyCatcher3 (Catalog # TZC013).

Data and code availability

- Mass spectrometry data have been deposited in the ProteomeXchange Consortium (http://proteomecentral.proteomexchange.org) with the dataset identifier ProteomeXchange: PXD037026. Project Name: Identification of mono-ADPr readers by multilevel chromatin proteomics. Original imaging data are available at Mendeley Data https://doi.org/10.17632/fdnscb6pn8.1.
- No new code was generated in this study.
- Any additional information required to reanalyze the data reported in this paper is available from the lead contact upon request.

EXPERIMENTAL MODEL AND SUBJECT DETAILS

Cell culture, SILAC labeling, and drug treatments

U2OS cell lines were obtained, authenticated by STR profiling and confirmed mycoplasma free by ATCC cell line authentication services. Cells were routinely tested for mycoplasma contamination. ARH3-KO, PARP1-KO and HPF1-KO U2OS cell lines were generously provided by Ivan Ahel (University of Oxford). For the generation of RNF114-KO cell lines, the sgRNAs 5'-GCCGCTTACACGTGTCCGCA-3' aimed towards exon 1 (Clone 1) and 5'- AGCCGAAGAAGCCTGTCTGT-3' aimed towards exon 3 (Clone 2) of the RNF114 gene were designed using ChopChop https://chopchop.cbu.uib.no.[63] The sgRNA encoding plasmids were cloned using the Guide-it CRISPR/Cas9 System (TAKARA Bio Inc., Japan) with the pGuide-it-ZsGreen1 vector following the manufacturer's protocol. U2OS cells were separately transfected with the resulting plasmids using Xfect™ Transfection Reagent (TAKAR Bio Inc., Japan) by following the manufacturer's instructions. Two days after transfection, the cells were washed twice in PBS, trypsinized and resuspended in PBS with 10 % bovine serum and single zsGreen expressing cells were sorted into 96 well plates using FACS (FACS & Imaging Core Facility, MPI Age). Single cells were regrown and screened by western blotting using anti-RNF114 antibody. Clones lacking RNF114 expression were further validated by sequencing. Genomic DNA of potential clones and wildtype U2OS cells was isolated using NucleoSpin Tissue Kit (Macherey-Nagel, Germany).The sgRNA's target region of Clone 1 was then amplified using 5'-GGAGGAACGGGATACTTAGGAG-3' and 5'-GCAGAGCGGCAGCAAGATGG-3' as forward and revers primer, respectively. Additionally, 5'-CAAACTGGCCAGATCCCCATA-3' and 5'-GAGCGGCAGCAAGATGGC-3' were used as forward and revers primer for clone 1. The sgRNA's target region of Clone 2 was amplified using 5'-ATTTGTCGTCTTGTCTCCATGA-3' and 5'-GGAAAGACATGGACTCTTCCAC-3' as forward and revers primer, respectively. After gel extraction (GenElute Gel Extraction Kit, Sigma-Aldrich, USA) the amplicons were sequenced using the respective PCR primer. Sequencings were analyzed for deletions in RNF114 Exon 1 (Clone 1) or Exon 3 (Clone 2) by applying the sequencing to ICE CRISPR Analysis Tool https://ice.synthego.com[64] and subsequent checking for frame shifts leading to premature stop codons of the gene. Each cell line was cultured in Glutamax-DMEM supplemented with 10% bovine serum and 100 U/ml penicillin/streptomycin at 37°C and 5% CO_2.

For SILAC labeling,[65] U2OS cells were grown in DMEM for SILAC (Thermo Scientific) supplemented with unlabeled L-lysine (Silantes, Germany) and L-arginine (Silantes, Germany) for the light condition or isotopically labeled L-lysine ($^{13}C_6{}^{15}N_2$, Silantes, Germany) and L-arginine ($^{13}C_6{}^{15}N_4$, Silantes, Germany) for the heavy condition. Both light and heavy DMEM were supplemented with 10% dialyzed FBS (Thermo Scientific). Cells were cultured for more than seven generations to achieve complete labeling. Incorporation efficiency (>99%) was determined by MS.

To induce DNA damage, the cell medium was aspirated and replaced with 37 °C complete DMEM containing 1 or 2 mM H_2O_2 (as indicated in the figure legends) for the indicated times. For MMS treatments, 4 mM MMS was added to the cells in complete DMEM for 30 minutes. For PARP inhibition (Olaparib, Cayman Chemical) cells were treated with 1 μM Olaparib in complete DMEM for the indicated times (generally 30 min for U2OS WT and 24-48 h for U2OS ARH3-KO).

For live-cell experiments cells were seeded into either 8-well glass-bottom chambered coverglass (Zell Kontact or Thermo Fisher Scientific), μ-Slide 8-well polymer-bottom chambered coverslip (Ibidi), or μ-Dish 35 mm polymer-bottom coverslip.

For Hoechst presensitization (live-cell microscopy experiments), growth medium was aspirated and replaced with fresh medium containing 0.3 μg/mL Hoechst 33342 for 1 h at 37 °C. Immediately prior to imaging, growth medium was replaced with CO_2-independent imaging medium (Phenol Red-free Leibovitz's L-15 medium (Life Technologies) or Molecular Probes Live Cell Imaging Solution (Invitrogen) supplemented with 20% fetal bovine serum, 2 mM glutamine, 100 μg/mL penicillin and 100 U/mL streptomycin).

To generate stable cell lines complemented with GFP empty vector (EV), GFP-RNF114-WT, or GFP-RNF114-C176A; U2OS RNF114-KO cells were plated in 6 cm dishes and transiently transfected with the corresponding plasmid (EV, GFP-RNF114-WT, GFP-RNF114-C176A) using lipofectamine 2000 according to manufacturer's protocol at 1:2 ratio with 2 μg of DNA. After 24 h, the cells were transferred to 15 cm dishes and grown for 24 h. Afterwards, the media was replaced with complete DMEM supplemented with 500 μg/mL G-418 solution for 14 days to select for resistant cells with stably integrated plasmids. Then, cells were collected and sorted by cytometry to select alive cells expressing GFP. For each condition, cells with similar expression levels were selected and bulk sorted before plating into 24-well plates in complete DMEM without G-418.

Plasmids

EGFP-tagged RNF114 and SIRT6 were subcloned by Gateway Technology into pDEST-CMV-N-EGFP and pDEST-CMV-C-EGFP, respectively (this manuscript). pLNCX2 plasmid encodes EGFP (Empty Vector, Addgene). RNF114C176A plasmid was generated by mutagenesis PCR of pDEST-GFP-RNF114 using 5'-GGCATCGAGGCAGCTATCGGACAAACCACAGATTTGG-3' and 5'-CCAAATCTGTGGTTTGTCCGATAGCTGCCTCGATGCC-3' as forward and reverse primers, respectively. pmEGFP-WWE (encoding the WWE domain of RNF146), pmEGFP-DTX3L, pmCherry-C1, pmCherry-ARH3-WT, pmCherry-ARH3-D77/78N, pmCherry-HPF1-WT, pmCherry-HPF1-E284A mutant, pEGFP-HPF1-WT, and pEGFP-HPF1-E284A mutant were kindly provided by Dr. Sébastien Huet (University of Rennes). pmCherry-macroD2 (encoding the macrodomain of macroD2) was kindly provided by Dr. Gyula Timinszky (Biological Research Centre, Szeged, Hungary). FLAG-TRF1-FokI-WT and TRF1-FokI-D450A were kindly provided by Dr. Roderick J. O'Sullivan (University of Pittsburg).

METHOD DETAILS

Transfections and siRNA treatments

For transient expression, cells were transfected for ~ 24–48 h using polyethylenimide (PEI) for mass spectrometry and immunoprecipitation experiments, lipofectamine 2000 (Sigma) for FLAG-TRF1-FokI and live-cell microscopy, and XtremeGENE HP (Sigma) for live-cell microscopy according to manufacturer's instructions. Details of plasmids generated, used, and origin are in the supplementary materials.

Chromatin extraction

For cellular fractionation experiments, U2OS cells were chemically fractionated using a subcellular protein fractionation kit (Thermo Fisher Scientific, USA), according to manufacturer's instructions with minor modifications. All buffers were kept on ice and freshly supplemented with 1x protease inhibitor (Thermo Fisher Scientific, USA), 1 μM Olaparib, and 1 μM ADP-HPD. Cells were gently scraped from the surface of a 10 cm tissue culture dish into PBS and recovered by centrifugation at 500 xg for 5 min at 4 °C. 100 μl of CEB buffer was added to the cell pellet and the tube incubated for 10 min on ice. Following centrifugation at 500 xg for 5 min at 4 °C, the supernatant (cytoplasmic extract) was transferred to a clean tube on ice and stored at -20 °C. 100 μl of MEB buffer was added to the cell pellet, vortexed for 5 sec, and incubated for 10 min at 4 °C with gentle agitation. Following centrifugation at 3000 xg for 5 min at 4 °C, the supernatant (membrane bound extract) was transferred to a clean tube on ice and stored at -20 °C. 50 μl of NEB buffer was added to the cell pellet, vortexed for 15 sec, and incubated for 30 min at 4 °C with gentle agitation. Following centrifugation at 5000 xg for 5 min at 4 °C, the supernatant (nuclear soluble extract) was transferred to a clean tube on ice and stored at -20 °C. 50 μl of NEB buffer supplemented with Micrococcal Nuclease and $CaCl_2$ was added to the pellet, vortex for 15 sec, and incubated at 37 °C for 6 min. After incubation, the tube was vortexed for 15 sec and centrifuged at 16,000 xg and the supernatant (chromatin bound nuclear extract) was transferred to a clean pre-chilled tube on ice.

Of the final 50 μl of the chromatin bound extract, 10 μl were used for immunoblotting and the remaining 40 μl were processed with acetone precipitation (as described in STAR Methods) for LC-MS/MS preparation.

Acetone precipitation of proteins

For mass-spectrometry analysis of chromatin samples, proteins were purified and desalted using a standard acetone precipitation protocol. Briefly, 4x volumes of ice-cold acetone was added to the protein sample and incubated at -20 °C overnight. Following incubation, the samples were centrifuged at 16,000 xg at 4 °C for 10 min and the supernatant was aspirated and discarded. The protein pellet was washed in ice-cold acetone, centrifuged at 16,000 xg for 5 min at 4 °C and the supernatant discarded. This step was repeated once more. The final protein pellet was air-dried to remove all traces of acetone.

Recombinant protein production

E. coli codon-optimized coding sequences for RNF114 and corresponding mutants (see Figure 6C) were transferred into pGEX-6P-3 vectors. Transformed BL21 strains were grown until OD_{600} = 0.6 and protein expression induced by 0.2 mM IPTG for 16h at 18 °C. Bacteria cells were collected, resuspended in lysis buffer (50 mM HEPES pH 7.9, 200 mM NaCl, 2.5 mM MgCl2, 5% glycerol, 1 mM DTT, supplemented with 0.25 mM PDMS, EDTA-free protease inhibitor cocktail, 1 mg/ml lysozyme, 75 U/ml benzonase) at 1:4 bacteria weight to buffer volume ratio and incubated on ice for 30 min. The suspension was probe-sonicated for 30 sec on ice, then centrifuged at 18,000 xg for 45 min at 4 °C. The supernatant was collected and recombinant proteins purified by incubation with Glutathione Sepharose 4B beads overnight at °C, washed trice with ice-cold PBS and eluted with elution buffer (50 mM HEPES pH 7.9, 200 mM NaCl, 1 mM DTT, 20 mM reduced glutathione). The eluate was aliquoted and stored at -80 °C.

Preparation of nuclear extract for peptide and nucleosome immunoprecipitation

U2OS WT cells were washed in ice-cold PBS, cells were scraped from the surface of a 10 cm tissue culture dish into PBS and recovered by centrifugation at 500 xg for 5 min at 4 °C. The pellet was resuspended in hypotonic buffer (10 mM HEPES pH 7.9; 10 mM KC1; 0.1 mM EDTA; 0.1 mM EGTA; 1 mM DTT; 0.5 mM PMSF, supplemented with 1 μM Olaparib, 1 μM ADP-HPD, and 1x EDTA-free protease inhibitors), incubated for 15 min on ice. Following incubation, NP-40 to a final concentration of 0.6% was added and the sample vortexed for 10 sec. The sample was centrifuged at 14,000 xg for 30 sec at 4 °C. The supernatant (cytosolic fraction) was discarded. The pellet was washed once in hypotonic buffer and centrifuged at 14,000 xg for 30 sec at 4 °C. The pellet was resuspended in ice-cold salt extraction buffer (20 mM HEPES pH 7.9, 420 mM NaCl, 0.2 mM EDTA, 0.1% NP-40, 20% glycerol, supplemented fresh with 1 μM Olaparib, 1 μM ADP-HPD, 1x protease inhibitors (EDTA free), 1 mM DTT) and incubate with end-over-end rotation for 30 min at 4 °C. After incubation, the sample was centrifuged at 16,000 xg for 10 min at 4 °C. Following centrifugation the supernatant (nuclear extract) was collected, diluted to a final concentration of 150 mM NaCl and ~ 0.6 μg/μL proteins, flash frozen in liquid nitrogen, and stored at -80 °C until further processing for nucleosome and peptide pulldowns.

Peptide and nucleosome immunoprecipitation

Peptide and nucleosome immunoprecipitation were performed as previously described.[66] For peptide pulldowns, 1 μg of biotinylated H3S10ADPr or H3WT (control) peptide was bound to 75 μL Dynabeads MyOne C1 streptavidin (Pierce) in binding buffer (20 mM HEPES pH 7.9, 150 mM NaCl, 0.1 % NP-40, 20% glycerol, supplemented fresh with 1 mM DTT) for 20 min at RT. Following extensive washing of unbound peptides, the beads-peptide mix was incubated with 500 μg of ice-cold nuclear extract (see STAR Methods for nuclear extract preparation) with end-over-end rotation for 2 h at 4 °C. Following incubation, the beads were washed twice in binding buffer, and twice in binding buffer without NP-40 and glycerol (20 mM hepes pH 7.9, 150 mM NaCl, supplemented fresh with 1 mM DTT). Elution and digestion for mass spectrometry were performed with incubation with 50 μL of digestion buffer (50 mM TEAB pH 8.5, 2.5 mM TCEP, 10 mM CAA, 0.002 μg/mL Lys-C, 0.02 μg/mL Trypsin) for ~ 14-16 h at 37 °C. The eluate was collected and processed with stagetips as described in STAR Methods.

Nucleosome pulldowns were performed as described above, with minor modifications. Briefly, 5 μg of WT nucleosome or H3S10ADPr nucleosome (with biotinylated 601 DNA sequence) was bound to 75 μL Dynabeads MyOne T1 streptavidin (Pierce).

Co-immunoprecipitation

48 h post-transfection U2OS cells were either left untreated or treated with 2 mM H2O2 for 30 min. Following one wash in ice-cold PBS, cells were scraped from the surface of a 10 cm tissue culture dish into PBS and recovered by centrifugation at 500 xg for 5 min at 4 °C. The cell pellet was then resuspended in 100 μl of Lysis Buffer (20 mM HEPES pH 7.9, 300 mM NaCl, 2.5 mM $MgCl_2$, 0.5% NP-40, 20% glycerol, 750 U/ml Benzonase, 10 μM Olaparib, 1X ADP-HPD, 1X EDTA-free protease inhibitor) and incubated on end-over-end rotation for 1 h at 4°C. After quenching the reaction with 100 μl of Quenching Buffer (20 mM HEPES pH 7.9, 30 mM EDTA, 10 μM Olaparib, 1X ADP-HPD, 1X EDTA-free protease inhibitor) the lysate was clarified at 20,000 xg for 10 min at 4°C and the supernatant from this step was collected in fresh tubes, supplemented with 300 μl of Dilution Buffer (20 mM HEPES pH 7.9, 300 mM NaCl, 0.5 mM EDTA, 0.5% NP-40, 10 μM Olaparib, 1X ADP-HPD, 1X EDTA-free protease inhibitor) to a final volume of 500 μl. 50 μl aliquots were kept as *Input* samples and the remaining 450 μl of cell lysate was mixed with 10 μl bed volume of GFP-Trap magnetic particles M-270 (Chromotek, Munich, DE) and incubated for 1 h with end-over-end rotation at 4 °C. Beads were recovered using a magnetic separation rack and washed 5 times with Washing Buffer (10 mM HEPES pH 7.9, 150 mM NaCl, 0.5 mM EDTA, 0.5% NP-40). 50 μl of 2X Laemmli sample buffer (50 mM DTT) was then added and bound proteins were eluted by heating the beads at 95 °C for 5 min. Supernatants were collected in fresh tubes as *Bound* samples and resolved by SDS-PAGE, followed by detection of proteins by immunoblotting.

Histone purification

Histones were purified as previously described,[5] with minor modification. Briefly, cells were treated as indicated in the figure legends, washed twice with ice-cold PBS, collected by scraping in PBS and recovered by centrifugation at 500 xg for 5 min at 4 °C. Cells were lysed by rotation in 0.1 M H_2SO_4 on end-over-end rotation for 2 h at 4 °C. The lysate was centrifuged at 2,200 xg at 4 °C for 20 min. The resulting pellet containing non-soluble proteins and cell debris was discarded. The supernatant was transferred to a clean pre-chilled tube and supplemented with NaCl, EDTA, and DTT to a final concentration of 0.5 M NaCl, 2 mM EDTA, 1 mM DTT. 1M Tris-HCl pH 8.0

was added until the pH was neutralized. For ion exchange chromatography, sulfopropyl (SP)-Sepharose resin (S1799, Sigma-Aldrich) was packed into a column and pre-equilibrated with 10 bed volumes of binding buffer. The neutralized supernatant was passed through the column. The resin was washed with 10 bed volumes of binding buffer and 30 bed volumes of washing buffer (50 mM Tris-HCl, pH 8.0; 0.6 M NaCl; 2 mM EDTA; 1 mM DTT). Proteins were eluted with elution buffer (50 mM Tris-HCl, pH 8.0; 2 M NaCl; 2 mM EDTA; 1 mM DTT) in six fractions. Eluted proteins were precipitated overnight in 4% (vol/vol) PCA at 4 °C. The fractions were then centrifuged at 21,000 xg at 4 °C for 45 min, and the resulting pellets were washed with 4% PCA in water (2 × 1 ml), 0.2% HCl in acetone (2 × 1 ml) and acetone (2 × 1 ml). The pellet was air-dried to remove all traces of acetone.

Partial FASP

Partial FASP was performed as previously described.[5] Briefly, protein pellets obtained from histone purification were resuspended in 200 μl of 50 mM TEAB, pH 8.5 and loaded onto a pre-washed Vivacon 10 kDa cut-off filter. The filter was centrifuged at 10,000 xg for 20 min at RT to concentrate the proteins and then washed twice with 50 mM TEAB, pH 8.5. Protein digestion was performed with 50 μl of Trypsin Gold (Promega) at a ratio 1:2000 (trypsin:protein) for 20 min at RT. The digestion was stopped by adding 400 μl of 0.15% TFA and the resulting peptides were collected by centrifugation at 10,000 xg for 20 min at RT. 200 μl of 50 mM TEAB, pH 8.5 was added to the filter, and filters were centrifuged once more, collecting both flowthroughs in the same tube. The flowthrough (partial FASP fraction) was stage-tipped for downstream mass spectrometry processing.

Mono-ADPr immunoprecipitation of purified untreated histones

Cells were grown on 245 mm square dishes and harvested in ice-cold PBS on ice. Afterwards histones were purified as described above with special care using ice-cold buffers and keeping samples on ice. Concentration of purified histones was determined using Pierce Rapid Gold BCA Protein Kit. 40 μg histones were incubated with 20 μg mono-ADPr antibody (AbD43647, IgG) overnight in a thermoshaker at 4 °C. To ensure efficient binding a final protein concentration of 1 μg/μl was targeted using a 10x binding buffer (final concentration 1x: 50 mM MOPS, 10 mM Na2HPO4, 150 mM NaCl). Protein A agarose beads (ThermoFisher, #20333) were pre-washed in 1x binding buffer using a centrifugation column (Pierce, #89868) and mixed with the antibody/histone complex. The bead/sample mixture was incubated for 2 h at RT using a thermoshaker. Columns were spin at 0.6 xg for 1 min at RT and washed three times in 2 bed volumes 1x binding buffer. Histones were eluted twice in 1 bed volume 0.15% TFA and elutions were pooled and dried using a speedvac. Dried samples were recovered in an appropriate volume of 1X LDS-sample buffer containing DTT and immunoblotted as described.

Protein digestion and cleanup for mass spectrometry

Dried protein pellets from acetone precipitation were resuspended in digestion buffer (50 mM TEAB, pH 8.5; 2.5 mM TCEP; 10 mM CAA; 0.002 μg/mL Lys-C; 0.02 μg/mL Trypsin) and incubated for ∼14-16 h at 37 °C. The digestion was stopped with the addition of 0.15% TFA. Digested peptides were cleaned-up on C18 stagetips according to a standard protocol[5] with 100% MeOH conditioning buffer; 30% ACN, 0.15% TFA equilibration and elution buffer; 0.15% TFA washing buffer. After the elution step, the eluate was dried to completion in a speedvac (Eppendorf). The dried peptides were resuspended in 0.1% FA for injection.

LC-MS/MS data acquisition

Three different quantitative proteomics methodologies were used in this study. For quantitation of histone modifications, a SILAC approach was used in order to make quantitation independent of possible variations in the partial digestion procedure.

Data Independent Acquisition (DIA) data were collected using Boxcar-DIA methodology on an Orbitrap Fusion coupled to an Easy-nLC 1000 (Thermo Scientific). The column was a 50 cm in-house packed emitter (medium Poroshell 120, C18, 1.9um. Emitter CoAnn (MS Wil) – 75 micron – 15 micron tip). Running buffers were 0.1% FA in Water (A) and 0.1%FA in 80% Acetonitrile (B). A 60-minute gradient from 4%-30% B was run at 300 nl/min followed by a steeper washing phase and a fast wash gradient at 400 nl/min.

Boxcar-DIA acquisition – MS1 spectra were collected for m/z 400-800 Th at a resolution of 120k and an AGC of 300%. Targeted SIM scans were collected in 2 sets of 8 overlapping user-defined windows spanning the precursor range, also at 120k. DIA MS2 spectra were collected in 14 Th windows from 400-800 Th, with HCD fragmentation (NCE = 27) and a resolution of 15k over the m/z range 145-1450 Th. Exact method details can be extracted from the raw files provided.

Tandem-Mass-Tag (TmT) MS3 acquisitions were performed on an Orbitrap Fusion LUMOS equipped with a FAIMs-Pro interface and coupled to an Easy-nLC 1200 with a 50 cm Acclaim Pep-map column (Thermo Scientific) and a 20 micron CoAnn emitter (MS Wil). LC buffers were identical to those described above. A 90-minute gradient of 6-31% B was run at 250 nl/min and MS data were collected with FAIMs compensation voltage (CV) alternating between -50 and -70 volts. MS1 was collected at 60k resolution with m/z 350-1500. Multiply-charged precursors were selected by a Topspeed method (1 sec cycle time per FAIMs voltage), isolated (width 0.7 Th) in the ion trap and fragmented by CID (energy 35%). The daughter ions were analyzed in the ion trap at "Turbo" resolution and 10 MS2 masses were selected for re-isolation for MS3. MS3 for TmT reporter quantification was performed with HCD (NCE=65) and analyzed in the orbitrap at 50k resolution. Again, method details may be extracted from the raw files provided.

Data-dependent Acquisition (DDA) SILAC data were collected by a TopSpeed method on the Orbitrap Fusion LUMOS system described above. A 110-minute gradient ran from 1-31% Buffer B at 250 nl/min followed by a steeper washing phase. MS1 spectra were acquired for m/z 350-1500 at 60k resolution and 250% AGC alternating between two FAIMs CVs (-50 and -70). Topspeed cycle

time was 1 sec for each FAIMs channel. Precursors with charge states 3-9 (highest first) were isolated (quadrupole width 1.6 Th) and fragmented by electron transfer dissociation (ETD). MS2 spectra were collected at 60k resolution with an AGC of 100%. Again, method details may be extracted from the raw files provided.

In vitro HPF1/PARP1 ADP-ribosylation assay

Recombinant PARP1 (100 nM), H3 (1 μM), HPF1 (concentration ranging from 100 nM to 5000 nM, as indicated), and 1 μg/mL of sonicated DNA were incubated with or without 200 μM NAD^+ in a PARP reaction buffer (50 mM Tris-HCl, pH 7.5; 50 mM NaCl; 1 mM $MgCl_2$) for 20 min at RT. The reaction was stopped with the addition of 4x Laemmli loading buffer supplemented with 5 mM DTT. The samples were them boiled for 5 min at 95 °C and stored at -20 °C until further processing for immunoblotting.

Detection of ADP-ribosylated genomic DNA by dot blot assay

Control and *Taq*DarT-modified gDNA was prepared as previously described.[29] A 1:1 dilution series of the *Taq*DarT-modified gDNA was prepared and dotted along with the control gDNA both at a maximum of 1000 ng onto nitrocellulose membranes (Amersham Protran 0.45 NC nitrocellulose). The membranes were crosslinked with 1200 J using a Stratalinker UV crosslinker and immunoblotted for gDNA (autoanti-dsDNA, DSHB, 1:200) or for ADPr-gDNA using the indicated antibodies at a concentration of 1 μg/mL in 5 % (w/v) powdered milk in PBST for 1 hr at RT. Where applicable, secondary peroxidase-couple antibodies (Dako) were incubated at RT for 45 min and samples visualised on hyperfilms (GE) using an ECL western blotting detection kit (Pierce).

Recombinant ARH3/DarG treatment of ADPr-gDNA

A solution of 400 ng ADPr-gDNA was incubated with either buffer only, or buffer supplemented with 1 μM of recombinant ARH3 or DarG and incubated for 30 min at 37 °C before performing a dot blot assay as described above.

Colony formation assay

For colony formation assay, cells were plated at 400 cells/well in 6-well plates and grown for 14 days in Glutamax-DMEM supplemented with 10 % bovine serum and 100 U/ml penicillin/streptomycin at 37 °C and 5 % CO_2. Approximately 12 h after plating, cells were treated by replacing medium with MMS-containing medium. Cells were incubated for 14 days and resulting colonies were stained with 0.5 % crystal violet in 25 % methanol for 30 min, washed with water, and air-dried. The plates were scanned with EVOS FL Auto 2, and quantification was performed with ImageJ/FIJI.

NHEJ reporter assay

U2OS-EJ5 cells (kind gift of Dr. Jeremy Stark (Duarte, CA),[52] were transfected with control siRNA, siXRCC4, siBRCA1 or siRNF114 using Lipofectamine RNAiMAX Transfection Reagent (Thermo Fisher Scientific) according to manufacturer's pro-tocol. 36 hours later, the cells were transfected with mCherry and I-SceI expression vectors, pmCherry-C1 and pCBASceI, respectively, using Xfect (Takara). pCBASceI was a gift from Maria Jasin (Addgene plasmid # 26477[67]). 48 hours after I-SceI transfection the cells were subjected to analysis with CytoFLEX S flow cytometer (Beckman Coulter Life Sciences). The percentages of GFP+ cells were determined within the mCherry expressing cell populations, representing the frequency of NHEJ events. Quantification was done by Kaluza Analysis Software (Beckman Coulter Life Sciences).

Affinity maturation, determination of antibody affinity, and SpyTag coupling

The antibody AbD33204 derived from the Fab phage display library HuCAL PLATINUM[68] was used for affinity maturation as previously described.[69] To maintain, and possibly extend, the broad mono specificity of the parental antibody, we used a selection strategy based on three different mono-ADPr peptides. During panning, a mixture of H3S10ADPr and PARP7S39ADPr (a mimic of the PARP7C39ADPr site) peptides were used as antigens, whereas the corresponding unmodified peptides were used for counter-selection. After initial panning for high-affinity clones, the PARP1S499ADPr peptide was added as an additional screening peptide. To identify successful affinity maturated clones, a subset of the clones was screened with ELISA (see "indirect enzyme linked immunosorbent assay (ELISA)" section in the STAR Methods for technical details) using H3S10ADPr, PARP7S39ADPr, PARP1S499ADPr, corresponding unmodified peptides, and free poly-ADPr. The corresponding antibody affinity was quantified (see below) to determine affinity and specificity compared to the parental antibody AbD33204. Affinity determination of monovalent HuCAL® Fab antibodies generated by Bio-Rad AbD Serotec GmbH (Germany, Puchheim) was performed on an Octet® RH16 instrument (Sartorius) at 30°C using 384-well microplates (Greiner Bio-One, No. 781209) that were agitated at 1000 rpm. Each of the purified Fab antibodies (molecular mass 52 kDa) was measured at five concentrations between 10 and 0.65 μg/mL (200 nM – 12.5 nM), and diluted in running buffer (PBS with 0.1% (w/v) bovine serum albumin (BSA), and 0.02% (v/v) Tween® 20). Biotinylated antigen H3-S10ad was immobilized on streptavidin (SA) biosensors (Sartorius, No. 18-5021) in phosphate-buffered saline (PBS) at 10 μg/mL for 10 min and blocked with 10 μg/ml biocytin. Typical immobilization levels were 2.6 ± 0.1 nm. After loading, sensors were kept in PBS until needed. Between measurements, the H3-S10ad-loaded biosensor surfaces were regenerated by exposing them to 10 mM glycine (pH 3.0), for 10 s followed by PBS for 30 s. Association phase was measured for 300 s, dissociation phase depending on the interaction for 200-400 s. All measurements were corrected for baseline drift by subtracting a control sensor exposed to running buffer only. Data were analyzed using a 2:1 interaction model for heterogeneous binding (fitting global, Rmax unlinked by sensor) on the Sartorius data

analysis software 10.0.3.1. Values are only shown for the binding event, which reflects the interaction as closely as possible. Spy-Tag coupling was performed as previously described.[24] Briefly, SpyTagged proteins and SpyCatchers were mixed in PBS and incubated for 1 h at room temperature. For bivalent SpyCatcher proteins (IgG-like and HRP-coupled formats), a 25% molar excess of SpyTagged proteins over SpyCatcher was chosen to ensure complete labeling of all SpyTag-SpyCatcher coupled products.

Generation of H3S10ADPr nucleosomes via native chemical ligation

Recombinant or semi-synthetic nucleosomes (aka. designer Nucleosomes) were synthesized / purified / assembled as previously described[70] but without DNA barcoding. EpiCypher versaNucs® were created by individually ligating histone H3 tail peptides (aa1-31 (A29L) with S10 mono-ADPr) to a H3 tailless nucleosome precursor (H3.1Ndelta32 assembled on 147bp 5′ biotinylated 601 DNA). The resulting nucleosome products were confirmed to contain <5% free DNA, undetectable levels of peptide precursor, and ≥90% full-length H3.1 with the modification(s) of interest. See also Figures S3A and S3C–S3F.

Indirect enzyme-linked immunosorbent assay (ELISA)

Biotinylated peptides (2 μg/mL or 0.2 μg/mL) in PBS 0.1% Tween 20 (PBS-T) were immobilized on Pierce NeutrAvidin Coated Plates. After three washes with PBS-T, wells were blocked with PBS-T containing 5% BSA for 1 h at RT. Next, primary antibodies diluted to 2 μg/mL in PBS-T/5% BSA were added and incubated for 2 h at RT. After washing the wells five times with PBS-T, HRP-conjugated goat anti-human IgG F(ab') (1:5000 dilution in blocking buffer) was added and incubated for 1 h at RT. After washing the wells five times, QuantaBlu Fluorogenic Peroxidase Substrate Kit was used for detecting peroxidase activity following manufacturer instructions.

Competition ELISA

A solution of 61 nM H3S10ADPr biotinylated peptide in PBS-T was incubated on Pierce NeutrAvidin Coaded Plates for 30 min at RT. After three washes with PBS-T, wells were blocked with PBS-T containing 5% BSA for 1 h at RT. Meanwhile, adenosine, AMP, ADP, deoxy-AMP, ATP, GDP, CDP, ADP-ribose were dissolved in water to a final concentration of 17 mM and used to prepare three solutions with a final concentration of 688 μM, 43 μM, 5.5 mM. Equal volumes of a 0.1 μg/mL antibody solution (AbD33205, AbD33204, AbD43647 HRP-conjugated antibodies) was mixed with each small molecule solution to a final concentration of 0.05 μg/mL and 344 μM, 21.5 μM, 2.75 μM respectively. The antibody:small molecule solution was incubated for 1 h at RT. After blocking, the wells were washed three times with PBS-T and the antibody:small molecule solution was added and incubated for 2 h at RT. After washing the wells five times, QuantaBlu Fluorogenic Peroxidase Substrate Kit was used for detecting peroxidase activity following manufacturer instructions.

SDS cell lysate preparation

U2OS cells with different genetic backgrounds (WT, HPF1-KO, ARH3-KO) were untreated or, where indicated, treated with H_2O_2 and lysed in SDS buffer (4% SDS; 50 mM HEPES, pH 7.9), boiled for 5 min at 95°C, and sonicated for 10 cycles of 30 s on/off on a bioruptor (Diagenode) at 4 °C.

Immunoblotting

For western blot analysis, samples were subjected to a standard SDS-PAGE method. Proteins were transferred to PVDF or nitrocellulose membranes (Merck Millipore), with wet transfer overnight with 90 mA constant, or with Trans-Blot Turbo transfer system (Bio-Rad). Membranes were then blocked with TBS-T buffer (25 mM Tris-HCl, pH 7.5; 150 mM NaCl; 0.05% Tween-20 supplemented with 5% non-fat dried milk) and probed overnight with primary antibodies at 4 °C, followed by a 1 h incubation with peroxidase-conjugated secondary antibodies at RT. For pap format antibodies, the secondary antibody step was skipped and instead the membranes were directly developed after washing. Blots were developed using either ECL Select (GE Healthcare) or SuperSignal™ West Atto Ultimate Sensitivity Chemiluminescent Substrate (Thermo Scientific), and signals were captured using a ChemiDoc MP System (Bio-Rad). Dilutions used for the primary antibodies were: α-PARP1 diluted 1:1000; α-HPF1 diluted 1:1000; Mono/Poly-ADPr-reagent (Cell Signaling Technology) diluted 1:1000; α-H3-S10/S28ADPr (AbD33644) HRP-coupled diluted 0.05 μg/ml; α-mono-ADPr (AbD43647) HRP-coupled diluted 0.05 μg/ml; α-mono-ADPr (AbD43647) IgG-like diluted 2 μg/ml; α-mono-ADPr (AbD33204) IgG diluted 2 μg/ml; α-mono-ADPr (AbD33205) IgG diluted 2 μg/ml; α-PARP1S499-ADPr (AbD34251) IgG diluted 2 μg/ml; α-GFP (Living Colors, JL-8, Takara) diluted 1:5000; α-H3 (Ab9715S, Cell Signaling Technology) diluted 1:1000; α-RNF114 (HPA021184, Sigma) diluted 1:1000; α-GAPDH (mAb 6C5, Sigma) diluted 1:1000; α-SEPT2 (HPA018481, Sigma) diluted 1:1000; α-SIRT6 (8457S, Cell Signaling Technology) diluted 1:1000; α-mCherry (GTX128508, GeneTex) diluted 1:2000; α-PARP1 (ab32138, abcam). See Methods S1 for details regarding the HRP-coupled antibodies.

Immunoblotting with on-membrane recombinant proteins treatment

For the experiments in Figure S1J, immunoblotting was performed as described previously. After milk bloking, the membranes were incubated in 5 mL of 50 mM Tris-HCl, pH 7.5, 50 mM NaCl, 1 mM $MgCl_2$ with 1 μM recombinant ARH3, or 10 U of snake venom phosphodiesterase (svPDE) as indicated. The membranes were incubated for 3 h at room temperature before washing with TBS-T and proceeding with primary antibody incubation as detailed above.

Direct immunofluorescence (IF)

General IF protocol

Cells were cultured on glass coverslips, treated as indicated, and fixed with ice-cold methanol for 20 min at -20 °C or 4% formaldehyde at RT. Cells were washed three times, permeabilised with 0.5% Triton X-100 for 5 min, and blocked with 3% BSA for 5 min. Incubation with primary antibodies (AbD33205: 1:1000; AbD43647: 1:1000; PAR (10H): 1:100; α-53BP1 (612523, BD transduction laboratories) 1:5000, α-RIF1 (229656, abcam) 1:400.) was followed by two washes in PBS before adding the secondary antibodies and DAPI stain. Coverslips were incubated again in 0.5% Triton X-100, washed twice in PBS and mounted with Prolong Diamond Antifade (ThermoScientific). Cells were imaged using a Leica SP8-DLS inverted laser-scanning confocal microscope using 63X objective. Nuclei were identified based on DAPI signal and ADPr fluorescence intensities were quantified in the colocalizing DAPI stained regions using Fiji Software. For quantification 100 nuclei were counted per condition.

Quantification of mean grey values microscopy

Raw files from microscopy were imported to Fiji. After splitting channels, the DAPI based channel was processed to generate a mask using the following filters: Minimum, radius=4; Maximum, radius=4; Subtract Background, rolling=300; Enhance Contrast, saturated=0.01 normalize; Auto Threshold, method=Huang white. Afterwards, particles were analysed using size=10-Infinity and edges were excluded. The mask was used to define regions of interest (ROIs) which were transferred to the ADPr fluorescence channel and mean grey values were measured. Next, another ROI was drawn manually to measure background levels which were subtracted from previously measured values. All values were imported to GraphPad Prism and plotted in a grouped data table.

Immunofluorescence with on-slide recombinant protein treatment

For the experiments in Figure S1J, immunofluorescence was performed as described previously using methanol fixation. After milk bloking, the slides were incubated in 500 μL of 50 mM Tris-HCl, pH 7.5, 50 mM NaCl, 1 mM $MgCl_2$ with 1 μM recombinant ARH3, or 1 U of snake venom phosphodiesterase (svPDE) as indicated. The slides were incubated for 3 h at room temperature before washing with PBS and proceeding with primary antibody incubation as detailed above.

TRF1-FokI telomere experiments

Cells on glass coverslips were washed twice in PBS and fixed with 2% paraformaldehyde (PFA) for 10 min. Cells were permeabilized with 0.1% (w/v) sodium citrate and 0.1 % (v/v) Triton X-100 for 5 min and incubated with fresh blocking solution (1 mg/mL BSA, 10% normal goat serum, 0.1% Tween) for 30 min. Primary antibodies were diluted in blocking solution and added to cells for 1 h at RT or overnight in refrigerated conditions. Next, cells were washed 3 times with PBS for 5 min and incubated with Alexa-coupled secondary antibodies (594 nm) (Life Technologies) for 1 h at RT. Following 3 washes in PBS, cells were mounted on slides with Prolong Gold Antifade reagent with DAPI (Life Technologies). Once the Prolong Anti-fade polymerized and cured, cells were visualized by conventional fluorescence with 60X and/or 63X Plan λ objective (1.4 oil) using a Nikon 90I. For this set of experiments sample size was not predetermined.

For EdU methods

U2OS cells were transfected with TRF1-FokI plasmid as described above. Cells were pulsed with EdU (10mM) 1 h before harvest. Cells on glass coverslips were washed twice in PBS and fixed with 2% paraformaldehyde (PFA) for 10mins. Cells were permeabilized with 0.1% (w/v) sodium citrate and 0.1 % (v/v) Triton X-100 for 5mins. The Click-IT Plus EdU Cell Proliferation Kit with Alexa Flour 647 (Invitrogen) was used to detect EdU.

Antibody labeling

100 μg anti-monoADPr antibody AbD33205 in monovalent format was labelled with 50 μg DyLight 550 NHS-Ester labelling dye (Thermo Scientific #632263) as follows: The antibody was diluted with PBS to 1 μg/μl and added to the dried fluorophore and the mixture was incubated for 1 h at room temperature protected from light. The excessive fluorophore was removed using a PD minitrap G-25 (sigma Aldrich #28-9180-07) according to the manifacture's instructions. The elution was concentrated on a 10 kDa cut-off filter to final 0.5 μg/μl. Afterwards, fluorophore/antibody ratio was measured by a nanodrop. The labelled antibody was stored at 4 °C protected from light. See also Figure S2G.

Antibody loading

The antibody was loaded into the cell as described in Cialek et al.[71] with minor modifications. In brief, 10^5 U2OS cells were seeded onto 35 mM high glass bottom dishes (ibidi #81158) two days prior to bead loading. Glass beads (≤ 106 μm) were cleaned and acidic washed by a sodium hydroxide wash, followed by two sterile water washes and a final ethanol wash. Beads were dried under sterile conditions and irradiated by several rounds of UV light. Afterwards beads were transferred into a jar covered by a 105 μM polypropylene mesh (Bückmann #PP 105/106-25) to ensure an even monolayer of beads when added to the cells. 5 μg labelled antibody was diluted in 50 μl PBS. DMEM covering the cells was removed and cells were washed once with PBS. 50 μl antibody loading solution was added to the cells and immediately covered by a monolayer of glass beads. Afterwards, the dish was tapped 10 times on the bench using medium force and 2 ml DMEM medium was added to the cells to prevent drying. Cells were incubated for 5 minutes with antibody/bead mixture before carefully washing off the glass beads with DMEM. Fresh DMEM with 0.3 μg/ml Hoechst 33342 (Thermo Scientific #H3570) was added and incubated for 1 h at 37 °C. DMEM was replaced by live cell imaging solution (Invitrogen #A14291DJ). At this stage, cells are ready for live cells imaging as described in the "protein recruitment kinetics at sites of laser irradiation" section of the STAR Methods. See also Figure S2G.

Protein recruitment kinetics at sites of laser irradiation

The monitoring of protein recruitment kinetics at sites of laser irradiation was performed as described previously.[31] In brief, images were acquired either on a Ti-E inverted microscope from Nikon equipped with a CSU-X1 spinning-disk head from Yokogawa, a Plan APO 60x/1.4 N.A. oil-immersion objective lens and a sCMOS ORCA Flash 4.0 camera; or on an Olympus Spin SR spinning disc system equipped with a CSU-W1 spinning-disk head from Yokogawa (50 micron pinhole size), a UPLSAPO 100XS/1.35 N.A. silicon-immersion objective lens and a Hammamatsu sCMOS ORCA Flash 4.0 camera. Laser irradiation of Hoechst-presensitized cells was performed along a 10 or 16 μm-line through the nucleus with a continuous 405 nm laser set at 125-130 μW at the sample level. Cells were maintained at 37 °C with a heating chamber. Analysis of the image sequences was performed either automatically using a custom MATLAB routine as described previously,[31] or by manually segmenting the irradiated area as well as the whole nucleus on ImageJ/FIJI or Olympus CellSense. Mean fluorescence intensities with the irradiated area was background substracted, divided to the mean nuclear intensity to correct for imaging photobleaching, and then normalized to the signal prior to DNA damage.

Fluorescence correlation spectroscopy

Fluorescence Correlation Spectroscopy (FCS) experiments were performed on a Zeiss LSM880 confocal setup equipped with a C-Apo 40x/1.2 N.A. water immersion objective. Fluorescence of GFP and mCherry was excited at 488 nm and 561 nm, respectively, and single emitted photons were detected and counted on the GaAsP spectral detector with spectral detection windows of 500-550 nm and 580-650 nm, respectively. Raw photon fluctuation traces were acquired for 20 seconds and detrended for slow fluctuations using the Fluctuation Analyzer 4G software[45] before autocorrelation. Cells were maintained at 37°C with a heating chamber. Autocorrelation curves were fitted with the following effective diffusion model:

$$G(t) = \frac{1}{N}\left(1 + t\frac{4D}{\omega^2}\right)^{-1}\left(1 + t\frac{4D}{(s\omega)^2}\right)^{-1/2}, \qquad \text{(Equation 1)}$$

where N is the number of tagged molecules in the focal volume, D is the effective diffusion coefficient, ω is the radial radius of the focal volume and s is the shape factor. N and D are fitted parameters while ω and s are fixed and set to 160 nm and 6, respectively.

For the experiment in Figure 5I, Effective diffusion coefficient measured by FCS for GFP-RNF114 (left) and mono-ADPr probe (right). Left: WT or ARH3-KO U2OS cells were transfected with GFP-RNF114 alone or together with mCherry-tagged HPF1-WT and treated or not with 1 uM Olaparib for 24h. Right: WT or ARH3-KO U2OS cells were transfected with mono-ADPr probe alone or together with GFP-tagged HPF1-WT and treated or not with 1 uM Olaparib for 24h.

QUANTIFICATION AND STATISTICAL ANALYSIS

All data analysis was performed using GraphPad Prism, ImageJ, Microsoft Excel, MatLab, and/or R. Detection, colocalization, and quantification of cells were performed using the ComDet v.0.5.3 plugin for ImageJ (https://github.com/ekatrukha/ComDet). Differences were analyzed with several statistical tests, as indicated in figure legends. In all graphs, the mean ± SEM (standard error of the mean) or ± SD (standard deviation) is plotted, as indicated in figure legends. For significant results, ****$P<0.0001$, ***$P<0.001$, **$P<0.01$, and *$P<0.05$.

Bioinformatic analysis of mass spectrometry samples

For DIA analysis, the protein_groups file was processed with an in-house R script using LIMMA[72] for significance testing of $\log_2$ intensities. Multiple testing-corrected p-values <0.05 ($-\log_{10}$(adj. p val) > 1.3) were considered significant. The GO term enrichment analysis was performed with the DAVID online platform.[73]

For TMT analysis, proteomics data was analyzed using MaxQuant, version 1.6.17.0.[61] The isotope purity correction factors, provided by the manufacturer, were included in the analysis. Differential expression analysis was performed using limma, version 3.34.9,[72] in R, version 3.4.3 (https://www.R-project.org/).

Statistics on microscopy data

To calculate significance between multiple groups and conditions an ordinary two-way analysis of variance (ANOVA) test was conducted and Tukey's multiple comparisons post-test was performed. Calculated p-values are represented by asterisks on the graph and listed in the figure legends.

ADDITIONAL RESOURCES

Detailed protocol

Immunoblotting using the HRP-coupled SpyTag format (Methods S1, related to STAR Methods and Figure 1)

Molecular Cell, Volume *83*

Supplemental information

Modular antibodies reveal DNA damage-induced mono-ADP-ribosylation as a second wave of PARP1 signaling

Edoardo José Longarini, Helen Dauben, Carolina Locatelli, Anne R. Wondisford, Rebecca Smith, Charlotte Muench, Andreas Kolvenbach, Michelle Lee Lynskey, Alexis Pope, Juan José Bonfiglio, Eva Pinto Jurado, Roberta Fajka-Boja, Thomas Colby, Marion Schuller, Ivan Ahel, Gyula Timinszky, Roderick J. O'Sullivan, Sébastien Huet, and Ivan Matic

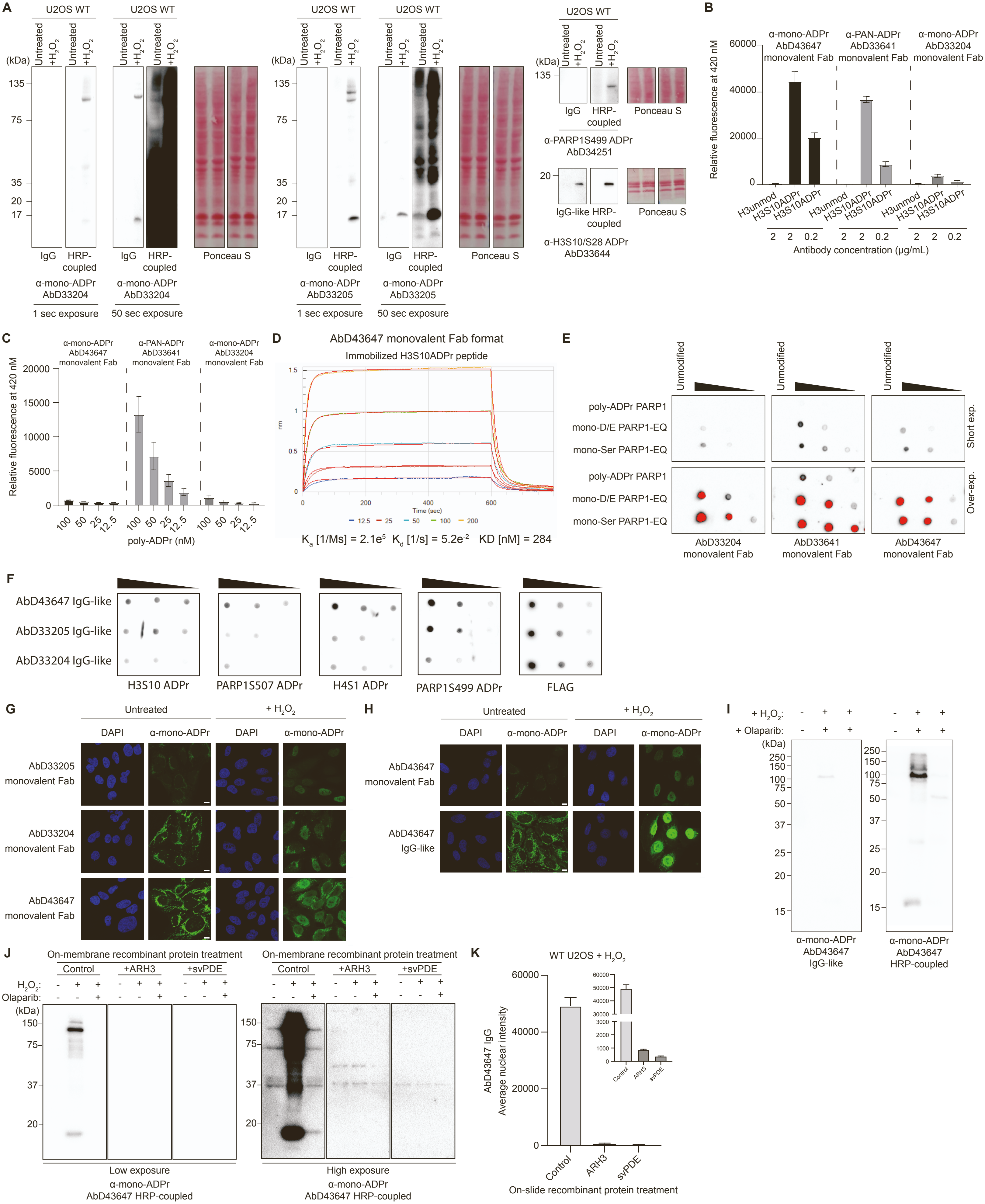
A
U2OS WT
Untreated
$+H_2O_2$
(kDa)
135
75
35
20
17
IgG
HRP-coupled
Ponceau S
α-mono-ADPr AbD33204
1 sec exposure
50 sec exposure
α-mono-ADPr AbD33205
α-PARP1S499 ADPr AbD34251
IgG-like HRP-coupled
α-H3S10/S28 ADPr AbD33644
B
Relative fluorescence at 420 nM
60000
40000
20000
0
α-mono-ADPr AbD43647 monovalent Fab
α-PAN-ADPr AbD33641 monovalent Fab
α-mono-ADPr AbD33204 monovalent Fab
H3unmod
H3S10ADPr
2
0.2
Antibody concentration (µg/mL)
C
α-mono-ADPr AbD43647 monovalent Fab
α-PAN-ADPr AbD33641 monovalent Fab
α-mono-ADPr AbD33204 monovalent Fab
Relative fluorescence at 420 nM
20000
15000
10000
5000
0
100
50
25
12.5
poly-ADPr (nM)
D
AbD43647 monovalent Fab format
Immobilized H3S10ADPr peptide
nm
1.5
1
0.5
0
200
400
600
Time (sec)
12.5
25
50
100
200
K_a [1/Ms] = 2.1e^5 K_d [1/s] = 5.2e^{-2} KD [nM] = 284
E
Unmodified
poly-ADPr PARP1
mono-D/E PARP1-EQ
mono-Ser PARP1-EQ
Short exp.
Over-exp.
AbD33204 monovalent Fab
AbD33641 monovalent Fab
AbD43647 monovalent Fab
F
AbD43647 IgG-like
AbD33205 IgG-like
AbD33204 IgG-like
H3S10 ADPr
PARP1S507 ADPr
H4S1 ADPr
PARP1S499 ADPr
FLAG
G
Untreated
+ H_2O_2
DAPI
α-mono-ADPr
AbD33205 monovalent Fab
AbD33204 monovalent Fab
AbD43647 monovalent Fab
H
Untreated
+ H_2O_2
DAPI
α-mono-ADPr
AbD43647 monovalent Fab
AbD43647 IgG-like
I
+ H_2O_2:
+ Olaparib:
(kDa)
250
150
100
75
50
37
25
20
15
α-mono-ADPr AbD43647 IgG-like
α-mono-ADPr AbD43647 HRP-coupled
J
On-membrane recombinant protein treatment
Control
+ARH3
+svPDE
H_2O_2:
Olaparib:
(kDa)
150
75
37
20
Low exposure
High exposure
α-mono-ADPr AbD43647 HRP-coupled
K
WT U2OS + H_2O_2
AbD43647 IgG Average nuclear intensity
60000
40000
20000
0
50000
30000
3000
2000
1000
Control
ARH3
svPDE
On-slide recombinant protein treatment

Figure S1. SpyTag-based modular antibodies enable sensitive and versatile detection of mono-ADPr, related to Figure 1

(**A**) Immunoblotting images showing comparison between IgG and HRP-coupled format for the indicated antibodies (see also **Figure 1A and S1I**). WT U2OS cells were either left untreated or treated with 2 mM H_2O_2 for 10 min and lysed in 4% SDS lysis buffer. (**B**) ELISA analysis of antibody sensitivity. The antibodies AbD33641, AbD33204, or the derived affinity maturated AbD43647 were incubated at the indicated concentrations with immobilized biotinylated peptides (H3S10unmodified or H3S10ADPr, 61 nM). Graphs showing mean ± SEM of n=2 technical replicates. (**C**) ELISA analysis of antibody specificity. The antibodies AbD33641, AbD33204, or the derived affinity maturated AbD43647 were incubated at 0.2 µg/ml with biotinylated free-poly-ADP-ribose at the indicated concentrations. Graphs showing mean ± SEM of n=2 technical replicates. (**D**) Affinity measurements of AbD43647, in monovalent Fab format. H3(1-21)S10ADPr was used as the immobilized antigen. K_a [1/Ms] = 2.1e^5 K_d [1/s] = 5.2e^{-2} KD [nM] = 284. (**E**) Dot blot analysis of *in vitro* automodified (i) PARP1 WT for poly-ADPr, (ii) PARP1-E988Q (PARP1-EQ) mutant in the absence of HPF1 for mono-ADPr on Asp/Glu, and (iii) PARP1-E988Q mutant in the presence of HPF1 WT for mono-ADPr on Ser. Bottom panels (high-exposed immunoblots of the top panels) show poly-ADPr of PARP1 WT. (**F**) Dot blot analysis of AbD43647, AbD33205, and AbD33204 antibodies blotted at 0.5, 0.25, 0.125 µg and incubated with the indicated biotinylated ADPribosylated peptides. Peptide binding was detected using anti-Streptavidin HRP antibody. (**G**) IF analysis of WT U2OS cells, untreated or treated with 2 mM H_2O_2 for 10 min, PFA fixed and stained with the indicated antibodies. (**H**) As in (**G**), IF analysis with the indicated antibodies. Detection of mono-ADPr in IF is dramatically improved upon conversion of Fab to bivalent IgG-like antibody. (**I**) Immunoblotting images showing comparison between IgG-like and HRP-coupled for the affinity maturated AbD43647. WT U2OS cells were treated with either 2 mM H_2O_2 for 10 min or 1 µM Olaparib for 30 min followed by 2 mM H_2O_2 for 10 min, and analyzed by immunoblotting with the indicated antibodies. Detection of mono-ADPr in WB is dramatically improved upon conversion of IgG-like to directly-coupled HRP antibody format. (**J**) Immunoblotting images showing that AbD43647 displays minimal signal after Serine-ADPr is removed by on-membrane recombinant ARH3 treatment or all forms of ADPr are hydrolyzed to phosphoribose by on-membrane svPDE treatment. (**K**) IF analysis of WT U2OS cells treated with 2 mM H_2O_2 for 10 min then fixed with Methanol and treated with the corresponding recombinant proteins. As in (**J**), AbD43647 display minimal nuclear signal after on-slide recombinant ARH3 and svPDE treatment.

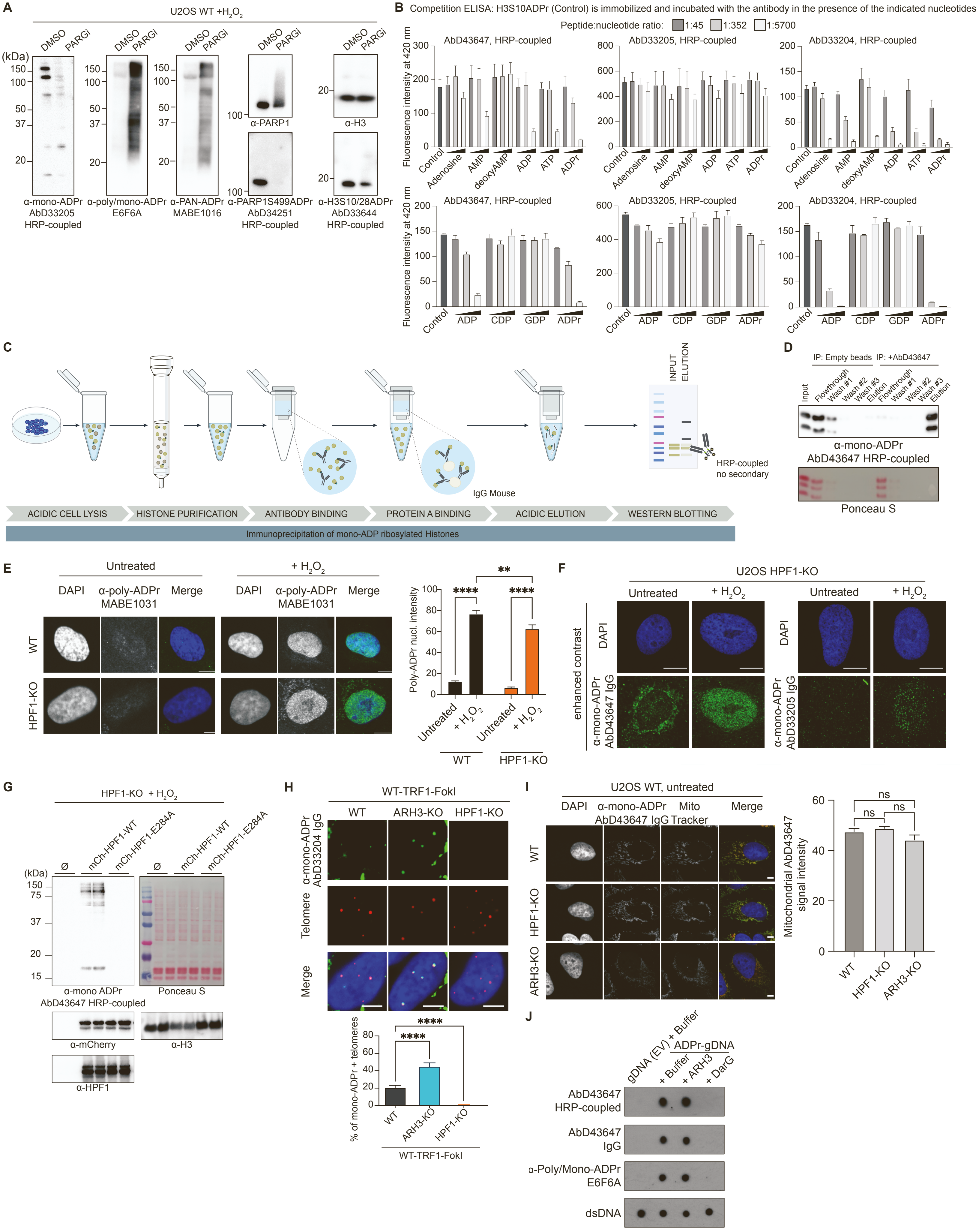

A
U2OS WT +H2O2
DMSO
PARGi
(kDa)
150
100
50
37
20
α-mono-ADPr
AbD33205
HRP-coupled
α-poly/mono-ADPr
E6F6A
α-PAN-ADPr
MABE1016
α-PARP1
α-PARP1S499ADPr
AbD34251
HRP-coupled
α-H3
α-H3S10/28ADPr
AbD33644
HRP-coupled
B
Competition ELISA: H3S10ADPr (Control) is immobilized and incubated with the antibody in the presence of the indicated nucleotides
Peptide:nucleotide ratio: 1:45 1:352 1:5700
AbD43647, HRP-coupled
AbD33205, HRP-coupled
AbD33204, HRP-coupled
Fluorescence intensity at 420 nm
Control
Adenosine
AMP
deoxyAMP
ADP
ATP
ADPr
CDP
GDP
C
ACIDIC CELL LYSIS
HISTONE PURIFICATION
ANTIBODY BINDING
PROTEIN A BINDING
ACIDIC ELUTION
WESTERN BLOTTING
Immunoprecipitation of mono-ADP ribosylated Histones
IgG Mouse
INPUT
ELUTION
HRP-coupled
no secondary
D
IP: Empty beads
IP: +AbD43647
Input
Flowthrough
Wash #1
Wash #2
Wash #3
Elution
α-mono-ADPr
AbD43647 HRP-coupled
Ponceau S
E
Untreated
+ H2O2
DAPI
α-poly-ADPr
MABE1031
Merge
WT
HPF1-KO
Poly-ADPr nucl. intensity
****
**
F
U2OS HPF1-KO
Untreated
+ H2O2
DAPI
enhanced contrast
α-mono-ADPr
AbD43647 IgG
α-mono-ADPr
AbD33205 IgG
G
HPF1-KO + H2O2
Ø
mCh-HPF1-WT
mCh-HPF1-E284A
(kDa)
150
75
37
20
15
α-mono ADPr
AbD43647 HRP-coupled
Ponceau S
α-mCherry
α-H3
α-HPF1
H
WT-TRF1-FokI
WT
ARH3-KO
HPF1-KO
α-mono-ADPr
AbD33204 IgG
Telomere
Merge
% of mono-ADPr + telomeres
****
I
U2OS WT, untreated
DAPI
α-mono-ADPr
AbD43647 IgG
Mito
Tracker
Merge
WT
HPF1-KO
ARH3-KO
Mitochondrial AbD43647
signal intensity
ns
J
gDNA (EV) + Buffer
ADPr-gDNA
+ Buffer
+ ARH3
+ DarG
AbD43647
HRP-coupled
AbD43647
IgG
α-Poly/Mono-ADPr
E6F6A
dsDNA

Figure S2. Dependence of mono-ADPr on HPF1 and validation of live cell imaging probes, related to Figure 1

(**A**) Immunoblotting images showing the mono-ADPr specificity of AbD33205, AbD34251, and AbD33644. WT U2OS cells were treated with either DMSO or 1 µM PARGi for 20 min followed by 2 mM H_2O_2 for 10min and lysed in 4% SDS lysis buffer. Related to **Figure 1C**. (**B**) Competition ELISA analysis of antibody epitopes. The antibodies AbD33205, AbD33204, or the derived affinity maturated AbD43647 were incubated with increasing concentrations of ADPr-related small molecules: 0 (control), 2.75 µM, 21.5 µM, 344 µM then incubated with immobilized H3S10ADPr peptide (61 nM). The signal intensity represents the amount of leftover antibody bound to the peptide. AbD33205 is not significantly affected by the nucleotides under any of the conditions tested, reflecting the lack of reactivity towards free-ADPr and indicating a strong preference for peptide-bound ADPr. Conversely, AbD33204 and to a lesser extent AbD43647 can cross-react with free-ADPr. Binding of AbD43647 to ADPr requires adenine and a free 2'-hydroxyl group on the adenine-proximal ribose. Binding is further strengthened by presence of 2 phosphate groups and to a lesser extent adenine-distal ribose. Graphs showing mean ± SEM (n=3, technical replicates). (**C**) Schematic illustration showing the immunoprecipitation workflow used to detect mono-ADPr on purified histones coupled with sensitive immunoblot detection. Related to **Figure 1D.** (**D**) Immunoblotting images showing selective enrichment of ADPribosylated histones upon pulldown with AbD43647. Related to **Figure 1D.** (**E**) IF analysis of WT or HPF1-KO U2OS cells, untreated or treated with 2 mM H_2O_2 for 10 min, methanol fixed and stained for poly-ADPr with the MABE1031 antibody. Related to **Figure 1E**. Data is mean ± SEM from a representative of n = 3 biological replicates. **** $P < 0.0001$; ** $P < 0.01$ (two-way ANOVA). (**F**) Enhanced contrast of the images from **Figure 1E**, showing mono-ADPr levels detected by AbD43647 in HPF1-KO U2OS cells upon treatment with 2 mM H_2O_2 for 10 min. (**G**) HPF1-KO U2OS cells were mock transfected or transfected with mCherry-HPF1-WT or mCherry-HPF1-E284A, treated with 1 mM H_2O_2 for 20 min, lysed and subjected to immunoblotting with the indicated antibodies. The two side-by-side lanes represent biological replicates. (**H**) Top: IF images of WT, ARH3-KO, and HPF1-KO U2OS cells transfected with FLAG-tagged WT-TRF1-FokI - to induce telomere-localized DNA damage - or DA-TRF1-FokI (D450A, catalytic dead mutant which does not induce DNA damage), fixed with methanol/acetone and stained with the indicated antibodies. Bottom: corresponding quantification of of mono-ADPr positive telomeres (%). Results are mean ± SEM. n = 3 biological replicates, 50 cells. **** $P < 0.0001$ (one-way ANOVA). Related to **Figure 1F.** Scale bars: 5 µm. (**I**) IF images (left) of WT, ARH3-KO, and HPF1-KO U2OS cells treated with MitoTracker red to stain mitochondria (500 nM for 15 min), fixed with PFA and stained with the indicated antibodies. Scale bars: 5 µm. Corresponding quantification (right) of AbD43647 signal overlapping with mitochondria staining. Data is mean ± SEM from a representative of n = 3 biological replicates. ns, not significant (unpaired Student's t test). Related to **Figure 1G**. (**J**) Dot blot of genomic DNA (gDNA) ADPr treated with 1 µM recombinant ARH3 or DarG for 30 min at 37 °C before staining with the indicated antibodies. Related to **Figure 1H**.

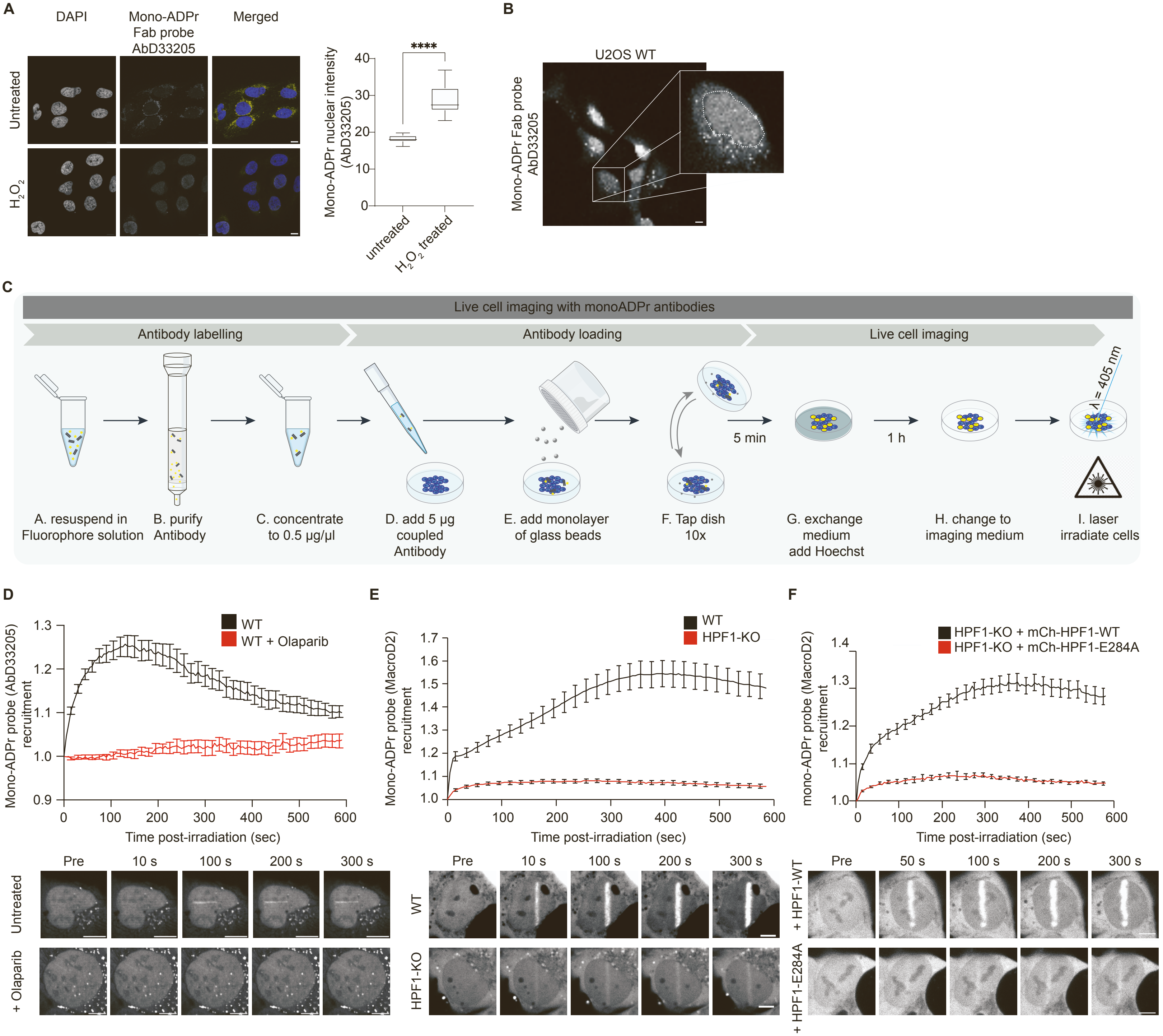
A
DAPI
Mono-ADPr Fab probe AbD33205
Merged
Untreated
H2O2
Mono-ADPr nuclear intensity (AbD33205)
****
untreated
H2O2 treated
B
U2OS WT
Mono-ADPr Fab probe AbD33205
C
Live cell imaging with monoADPr antibodies
Antibody labelling
Antibody loading
Live cell imaging
λ = 405 nm
5 min
1 h
A. resuspend in Fluorophore solution
B. purify Antibody
C. concentrate to 0.5 µg/µl
D. add 5 µg coupled Antibody
E. add monolayer of glass beads
F. Tap dish 10x
G. exchange medium add Hoechst
H. change to imaging medium
I. laser irradiate cells
D
WT
WT + Olaparib
Mono-ADPr probe (AbD33205) recruitment
Time post-irradiation (sec)
Pre
10 s
100 s
200 s
300 s
Untreated
+ Olaparib
E
WT
HPF1-KO
Mono-ADPr probe (MacroD2) recruitment
Time post-irradiation (sec)
Pre
10 s
100 s
200 s
300 s
F
HPF1-KO + mCh-HPF1-WT
HPF1-KO + mCh-HPF1-E284A
mono-ADPr probe (MacroD2) recruitment
Time post-irradiation (sec)
Pre
50 s
100 s
200 s
300 s
+ HPF1-WT
+ HPF1-E284A

Figure S3. Fluorescence-based sensors reveal DNA damage-induced serine mono-ADPr as second wave of PARP1 signaling, related to Figure 2

(**A**) IF analysis of WT U2OS cells treated or not with 2 mM H_2O_2 for 10 min, fixed and stained with AbD33205 coupled to dyLIGHT550, used as live cell mono-ADPr Fab probe (see also **Figure 1A and 2A**). Data is mean ± SEM from a representative of n = 3 biological replicates. **** $P < 0.0001$ (unpaired Student's t test). (**B**) Representative confocal image of AbD33205 coupled to dyLIGHT550 (mono-ADPr Fab probe) showing nuclear localization of the antibody loaded into live cells using the bead-loading method. (**C**) Schematic illustration showing the antibody labeling, bead-loading and laser microirradiation workflow used to detect mono-ADPr in live cells. Briefly, an antibody solution is added to the cells and immediately covered by a monolayer of beads. The antibodies are not attached to the glass beads and the beads do not enter the nuclei, but are simply used to induce a temporary mechanical disruption to the cell membrane, allowing macromolecules, including antibodies, to enter the cells. After incubation, the beads are washed off and the medium replaced by live cell imaging solution. See also the "Antibody labelling", "Antibody loading", and "Protein recruitment kinetics at sites of laser irradiation" sections of the STAR Methods for details. (**D**) Top: recruitment kinetics of mono-ADPr Fab probe (AbD33205) in WT U2OS cells untreated (black) or treated with 30 μM Olaparib immediately prior to irradiation (red). The mean ± SEM from 10 cells from a representative of 3 independent experiments is shown. Bottom: representative confocal images. Scale bars: 10 μm. (**E**) Top: recruitment kinetics of mono-ADPr probe (GFP-tagged macrodomain of macroD2) to sites of DNA damage induced by laser irradiation, in WT and HPF1-KO U2OS cells. The mean ± SEM from 10–12 cells from a representative of 3 independent experiments is shown. Bottom: representative confocal images. Scale bar: 5 μm. (**F**) Top: recruitment kinetics of mono-ADPr probe (GFP-tagged macrodomain of macroD2) to sites of DNA damage induced by laser irradiation in HPF1-KO U2OS cells expressing mCherry-tagged HPF1-WT or HPF1-E284A. The mean ± SEM from 13–16 cells from a representative of 3 independent experiments is shown. Bottom: representative confocal images. Scale bar: 5 μm.

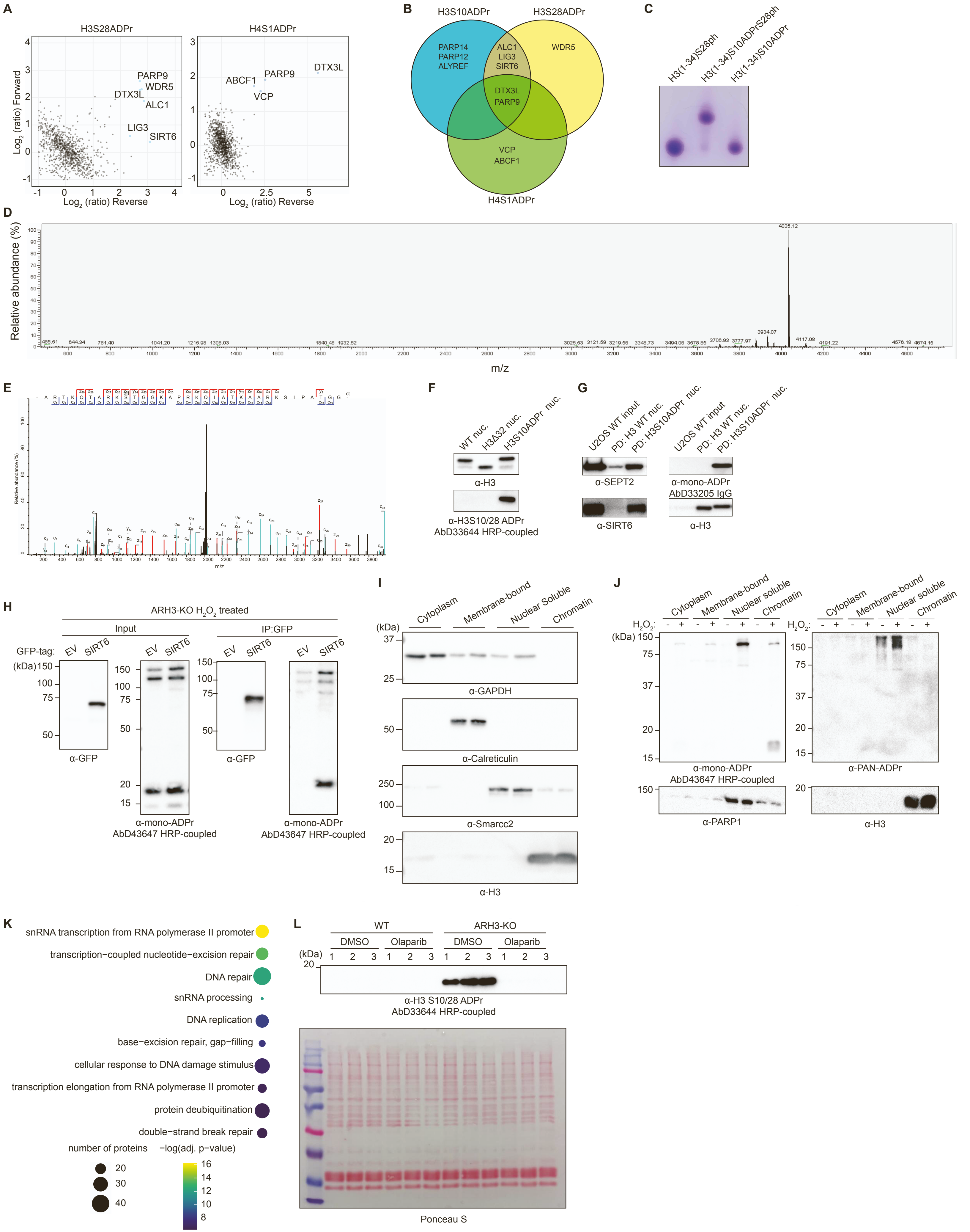

A
H3S28ADPr
H4S1ADPr
Log2 (ratio) Forward
Log2 (ratio) Reverse
PARP9
WDR5
DTX3L
ALC1
LIG3
SIRT6
ABCF1
VCP
B
H3S10ADPr
H3S28ADPr
H4S1ADPr
PARP14
PARP12
ALYREF
LIG3
WDR5
PARP9
VCP
ABCF1
C
H3(1-34)S28ph
H3(1-34)S10ADPrS28ph
H3(1-34)S10ADPr
D
Relative abundance (%)
m/z
E
F
WT nuc.
H3Δ32 nuc.
H3S10ADPr nuc.
α-H3
α-H3S10/28 ADPr
AbD33644 HRP-coupled
G
U2OS WT input
PD: H3 WT nuc.
PD: H3S10ADPr nuc.
α-SEPT2
α-mono-ADPr
AbD33205 IgG
α-SIRT6
α-H3
H
ARH3-KO H2O2 treated
Input
IP:GFP
GFP-tag:
EV
SIRT6
(kDa)
α-GFP
α-mono-ADPr
AbD43647 HRP-coupled
I
Cytoplasm
Membrane-bound
Nuclear Soluble
Chromatin
(kDa)
α-GAPDH
α-Calreticulin
α-Smarcc2
α-H3
J
Nuclear soluble
H2O2:
α-mono-ADPr
AbD43647 HRP-coupled
α-PAN-ADPr
α-PARP1
α-H3
K
snRNA transcription from RNA polymerase II promoter
transcription−coupled nucleotide−excision repair
DNA repair
snRNA processing
DNA replication
base−excision repair, gap−filling
cellular response to DNA damage stimulus
transcription elongation from RNA polymerase II promoter
protein deubiquitination
double−strand break repair
number of proteins
−log(adj. p−value)
L
WT
ARH3-KO
DMSO
Olaparib
α-H3 S10/28 ADPr
AbD33644 HRP-coupled
Ponceau S

Figure S4. Identification of mono-ADPr readers by chromatin proteomics, related to Figure 4

(**A**) Scatterplot showing proteins enriched by H3S28ADPr (left) and H4S1ADPr (right) peptides, compared to the unmodified peptide. Enriched proteins are shown in light blue. (Shown are n=2 forward and reverse samples as representative from 3 independent experiments). (**B**) Venn diagram illustrating the overlap between the H3S10ADPr, H3S28ADPr, H4S1ADPr peptide pulldown datasets, related to **Figure 4B and S4A**. (**C**) Inverted Polarity-TBE gel showing the steps of the phospho-guided ADPr reaction: (i) synthetic peptide precursor with phosphate as protecting group H3(1-34)S28ph; (ii) phosphopeptide ADP-ribosylated on the desired serine H3(1-34)S10ADPrS28ph; and (iii) site-specifically ADP-ribosylated peptide H3(1-34)S10ADPr. (**D**) Deconvoluted chromatogram showing purity of the H3(1-34)S10ADPr peptide. Mass = 4035.12. (**E**) ETD spectrum of H3(1-34)S10ADPr used for nucleosome generation showing site-specific ADPribosylation (ad) at the intended site. (**F**) WT unmodified nucleosomes, truncated nucleosomes used as input for native chemical ligation, and produced H3S10ADPr nucleosomes were probed with the indicated antibodies. (**G**) Immunoblot analysis of nucleosome pulldown showing enrichment of SEPT2 and SIRT6 to H3S10ADPr nucleosomes. PD = pulldown. Related to **Figure 4D.** (**H**) Immunoblotting images of ARH3-KO U2OS cells transfected with GFP- SIRT6, treated with 2 mM H_2O_2 for 30 min and subjected to immunoprecipitation using an anti-GFP antibody. Bound proteins were analyzed by immunoblotting using the indicated antibodies. (**I**) WT U2OS cells were subjected to subcellular fractionation, the resulting fractions were resolved by SDS-PAGE and fractionation efficiency was checked using GAPDH (Mainly cytosolic), Calreticulin (Membrane-bound), Smarcc2 (Nuclear Soluble), and H3 (Chromatin Bound). (**J**) WT U2OS cells were untreated or treated with 1 mM H_2O_2 for 20 min and subjected to subcellular fractionation. The samples were immunoblotted with the indicated antibodies. ADPr PARP1 is mainly nuclear soluble and ADPr histones mainly chromatin-bound, confirming previous reports (Prokhorova et al, mol cell). (**K**) Gene ontology (GO) analysis of the proteins with positive $\log_2$ fold change (enriched in H_2O_2-treated cells) from **Figure 4F**. Shown are the significant Biological Processes terms. (**L**) Ponceau-S loading control and corresponding mono-ADPr immunoblots of the chromatin extract samples used in the proteomics experiment in **Figure 4H-J**.

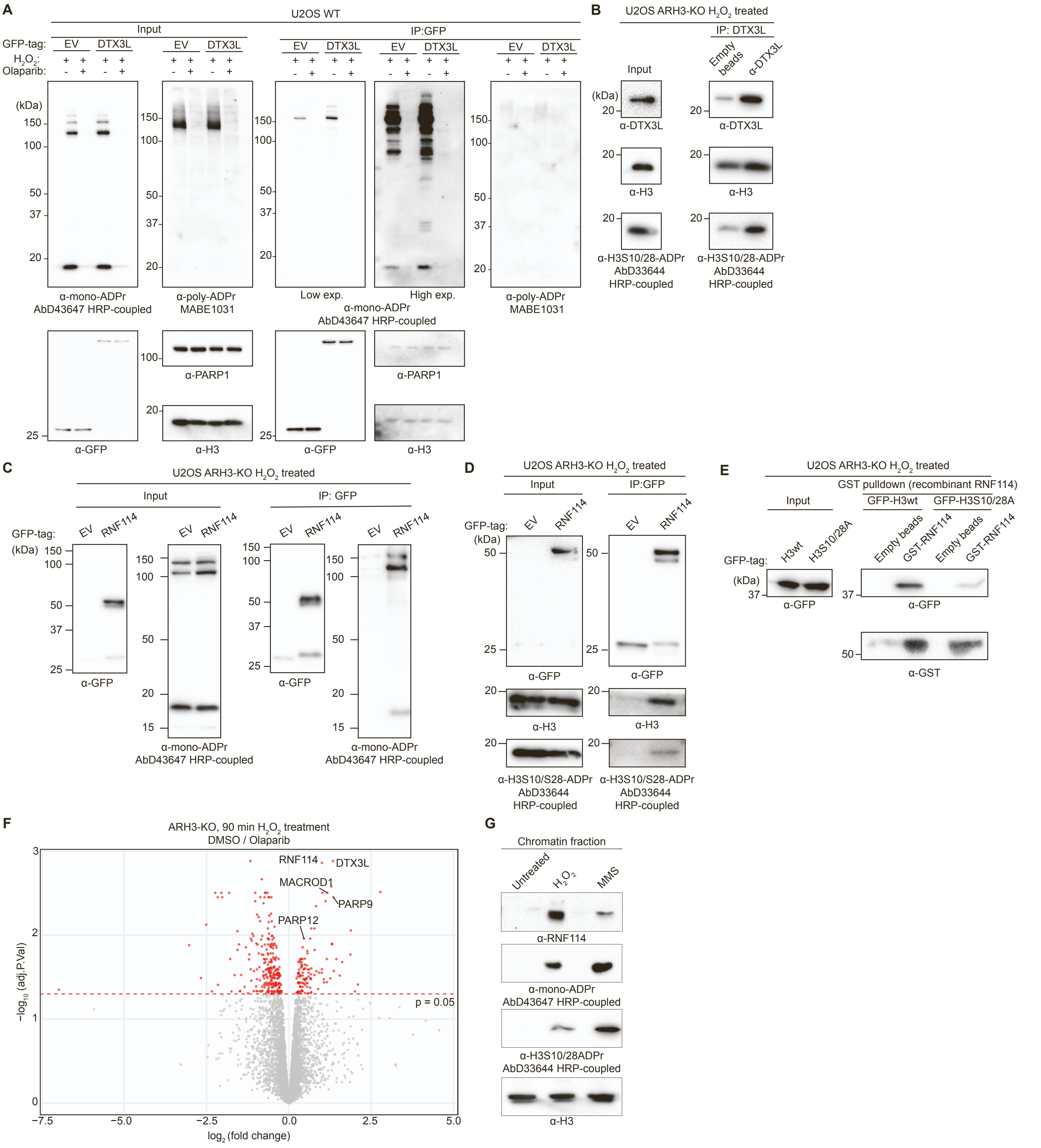
A
U2OS WT
Input
IP:GFP
GFP-tag: EV DTX3L EV DTX3L
H2O2:
Olaparib:
(kDa)
150
100
50
37
20
25
α-mono-ADPr
AbD43647 HRP-coupled
α-poly-ADPr
MABE1031
Low exp.
High exp.
α-PARP1
α-GFP
α-H3
B
U2OS ARH3-KO H2O2 treated
IP: DTX3L
Input
Empty beads
α-DTX3L
α-H3S10/28-ADPr
AbD33644
HRP-coupled
C
U2OS ARH3-KO H2O2 treated
IP: GFP
RNF114
15
D
IP:GFP
α-H3S10/S28-ADPr
E
GST pulldown (recombinant RNF114)
GFP-H3wt
GFP-H3S10/28A
H3wt
H3S10/28A
GST-RNF114
α-GST
F
ARH3-KO, 90 min H2O2 treatment
DMSO / Olaparib
RNF114
DTX3L
MACROD1
PARP9
PARP12
p = 0.05
−log10 (adj.P.Val)
log2 (fold change)
−7.5
−5.0
−2.5
0.0
2.5
5.0
G
Chromatin fraction
Untreated
H2O2
MMS
α-RNF114
α-H3S10/28ADPr
AbD33644 HRP-coupled

Figure S5. Validation of mono-ADPr readers identified by chromatin proteomics, related to Figure 4

(**A**) Immunoblotting images of WT U2OS cells, transfected with GFP-Empty Vector (GFP-EV) or GFP-DTX3L, treated with DMSO or 1 μM Olaparib for 30 min, then treated with 2 mM H_2O_2 for 30 min. Cell lysates were incubated with magnetic beads conjugated to an anti-GFP antibody. Bound proteins were immunoblotted and stained with the indicated antibodies. (**B**) Immunoblotting images of WT U2OS cells treated with 2 mM H_2O_2 for 30 min. Cell lysates were incubated with magnetic protein A beads, either empty or coupled to anti-DTX3L antibody. Bound proteins were immunoblotted and stained with the indicated antibodies. (**C, D**) Immunoblotting images of ARH3-KO U2OS cells, transfected with GFP-Empty Vector (GFP-EV) or GFP-RNF114, treated with 2 mM H_2O_2 for 30 min. Cell lysates were incubated with magnetic beads conjugated to an anti-GFP antibody. Bound proteins were immunoblotted and stained with the indicated antibodies. (**E**) Immunoblotting images of ARH3-KO U2OS cells transfected with GFP-tagged H3-WT or H3-S10/28A mutant, treated with 2 mM H_2O_2 for 30 min. Cell lysates were incubated with either empty GST agarose beads or beads coupled to GST-tagged RNF114. Bound proteins were immunoblotted and stained with the indicated antibodies. (**F**) ARH3-KO U2OS cells were treated with DMSO or 1 μM Olaparib for 48 h, then either left untreated or treated with 1 mM H_2O_2 for 90 min. Cells were then subjected to subcellular fractionation and the resulting chromatin bound fraction was analyzed by DIA LC-MS/MS. Volcano plot showing the log2-fold change of detected proteins. the red dotted line represents significance with P-value=0.05 (-$\log_{10}$(adj. P-value)>1.3) cut-off, Significant proteins are indicated in red. (n = 4, biological replicates). (**G**) U2OS WT cells were either left untreated, treated with 1 mM H_2O_2 for 20 min, or treated with 4 mM MMS for 30 min and subjected to subcellular fractionation. The chromatin bound fraction was analyzed by immunoblotting using the indicated antibodies.

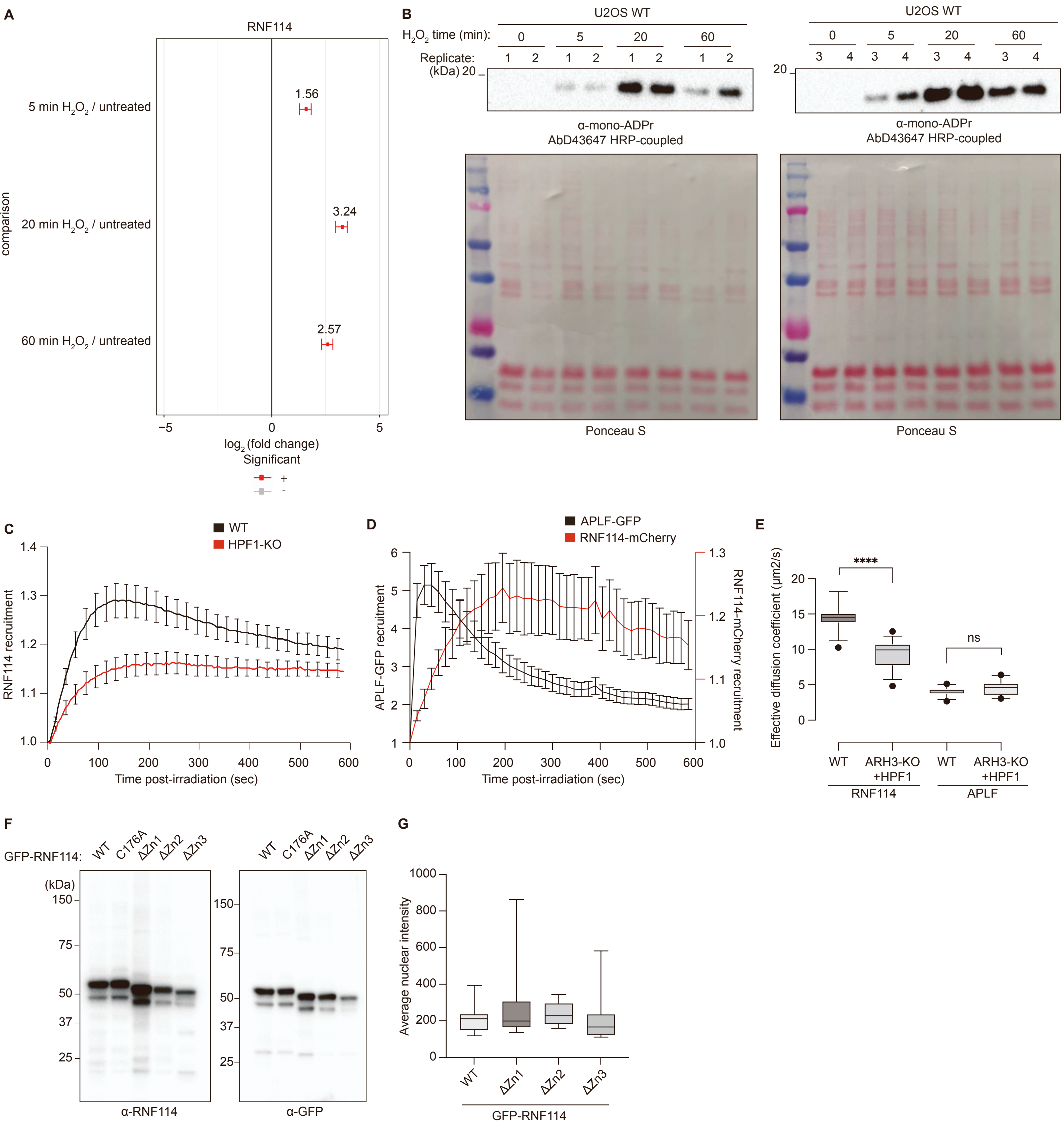
A
RNF114
5 min H_2O_2 / untreated
20 min H_2O_2 / untreated
60 min H_2O_2 / untreated
comparison
1.56
3.24
2.57
−5
0
5
log2 (fold change)
Significant
+
-
B
U2OS WT
H_2O_2 time (min):
0
5
20
60
Replicate:
1
2
3
4
(kDa) 20
α-mono-ADPr
AbD43647 HRP-coupled
Ponceau S
C
WT
HPF1-KO
RNF114 recruitment
1.4
1.3
1.2
1.1
1.0
0
100
200
300
400
500
600
Time post-irradiation (sec)
D
APLF-GFP
RNF114-mCherry
APLF-GFP recruitment
RNF114-mCherry recruitment
Time post-irradiation (sec)
E
Effective diffusion coefficient (µm2/s)
****
ns
WT
ARH3-KO
+HPF1
RNF114
APLF
F
GFP-RNF114:
WT
C176A
ΔZn1
ΔZn2
ΔZn3
(kDa)
150
75
50
37
25
α-RNF114
α-GFP
G
Average nuclear intensity
1000
800
600
400
200
0
WT
ΔZn1
ΔZn2
ΔZn3
GFP-RNF114

Figure S6. Chromatin mono-ADPr functions as a recruitment signal for RNF114, related to Figure 5 and 6

(**A**) Log_2 fold change plots for RNF114 corresponding to **Figure 5A**. (**B**) Ponceau S loading control and corresponding mono-ADPr immunoblots of the chromatin extract samples used in the proteomics experiment in **Figure 5A**. (**C**) Recruitment kinetics of GFP-RNF114 in WT (black) or HPF1-KO (red) U2OS cells. The mean ± SEM of 16 cells from a representative of 3 independent experiments is shown. Related to **Figure 5C**. (**D**) Recruitment kinetics of co-expressed GFP-tagged APLF (black) and mCherry-tagged RNF114 (red) in WT U2OS cells. The mean ± SEM from 16 cells from a representative experiment is shown. (**E**) Effective diffusion coefficient measured by FCS for RNF114 (left) and APLF (right). WT or ARH3-KO U2OS cells were transfected with GFP-tagged RNF114 or APLF alone or together with mCherry-tagged HPF1. ****, P-value < 0.0001; ns, not significant (unpaired Student's t test assuming unequal variances). n=3 independent replicates. (**F**) Immunoblotting analysis of the GFP-tagged RNF114-WT and corresponding mutants expressed in U2OS WT cells, see **Figure 6C**. (**G**) Corresponding relative average nuclear intensity for the constructs used in **Figure 6D**, showing that despite the lower expression level of RNF114-ΔZn3 (**Figure S6F**) for the live cell experiment we selected cells with similar expression levels across all constructs.

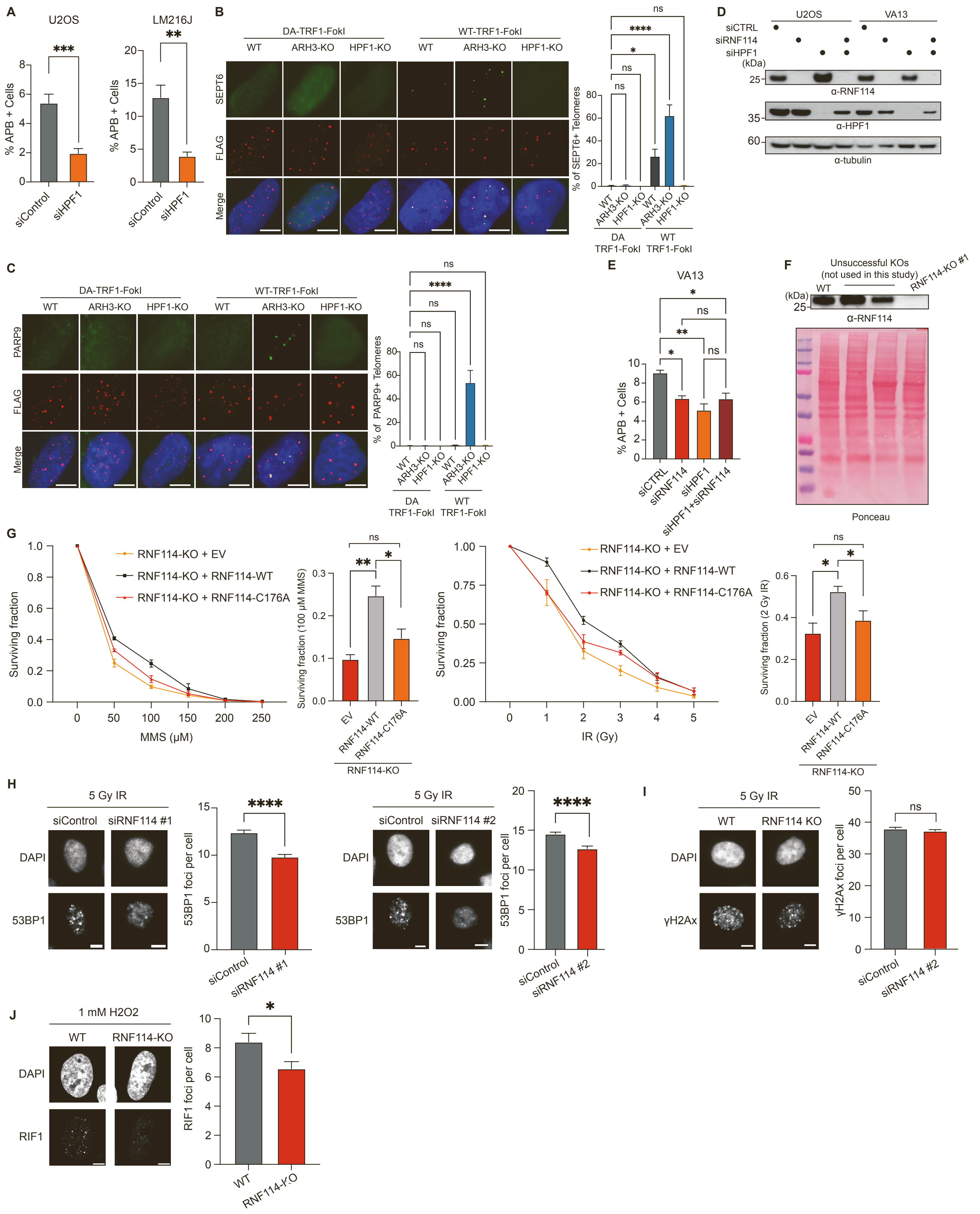

A
U2OS
LM216J
% APB + Cells
siControl
siHPF1
B
DA-TRF1-FokI
WT-TRF1-FokI
WT
ARH3-KO
HPF1-KO
SEPT6
FLAG
Merge
% of SEPT6+ Telomeres
DA TRF1-FokI
WT TRF1-FokI
ns
D
U2OS
VA13
siCTRL
siRNF114
siHPF1
(kDa)
α-RNF114
α-HPF1
α-tubulin
C
PARP9
% of PARP9+ Telomeres
E
VA13
% APB + Cells
siCTRL
siRNF114
siHPF1
siHPF1+siRNF114
F
Unsuccessful KOs (not used in this study)
RNF114-KO #1
α-RNF114
Ponceau
G
RNF114-KO + EV
RNF114-KO + RNF114-WT
RNF114-KO + RNF114-C176A
Surviving fraction
MMS (μM)
Surviving fraction (100 μM MMS)
EV
RNF114-WT
RNF114-C176A
RNF114-KO
IR (Gy)
Surviving fraction (2 Gy IR)
H
5 Gy IR
siControl
siRNF114 #1
siRNF114 #2
DAPI
53BP1
53BP1 foci per cell
I
WT
RNF114 KO
γH2Ax
γH2Ax foci per cell
siRNF114 #2
J
1 mM H2O2
RNF114-KO
RIF1
RIF1 foci per cell

Figure S7. RNF114 modulates the alternative lengthening of telomeres pathway and the DNA damage response, related to Figure 7

(**A**) Representative IF images (left) and quantification (right) of ALT-associated PML bodies (APBs) in U2OS and LM216J cells transfected with siControl or siRNA for HPF1 (siHPF1). ***, P-value < 0.001; **, P-value < 0.01. (unpaired Student's t test). (**B**) Left: Representative images of WT, ARH3-KO, or HPF1-KO U2OS cells co-transfected with FLAG-TRF1-FokI WT or FLAG-TRF1-FokI DA (D450A, catalytic dead mutant) and GFP-SEPT6, fixed and stained with the indicated antibodies. Right: quantification of SEPT6 positive telomeres (%). Data are mean ± SEM. (n = 3, biological replicates each 50 cells). ****, P-value < 0.0001; *, P-value < 0.05; ns, not significant (one-way ANOVA). Scale bars: 5 µm. (**C**) Left: Representative images of WT, ARH3-KO, or HPF1-KO U2OS cells co-transfected with FLAG-TRF1-FokI WT or FLAG-TRF1-FokI DA (D450A, catalytic dead mutant) and GFP-PARP9, fixed and stained with the indicated antibodies. Right: quantification of PARP9 positive telomeres (%). Data are mean ± SEM. (n = 3, biological replicates each 50 cells). ****, P-value < 0.0001; ns, not significant (one-way ANOVA). Scale bars: 5 µm. (**D**) Immunoblot images showing efficient siRNA of RNF114 (siRNF114) and HPF1 (siHPF1) in U2OS and VA13 cell lines. Related to **Figure 7C and S7E**. (**E**) Quantification of APBs in VA13 WT cells tranfected with siRNA for control (siControl), HPF1 (siHPF1), RNF114 (siRNF114), or HPF1 + siRNF114. Data are mean ± SEM. ** P-value < 0.01; * P-value < 0.05; ns, not significant. Related to **Figure 7C**. (**F**) Immunoblot images of WT and RNF114-KO U2OS cells. Ponceau was used as loading control. (**G**) Clonogenic cell survival assay of RNF114-KO U2OS cells stably complemented with either GFP-EV, GFP-RNF114-WT, or GFP-RNF114-C176A. DNA damage was induced by continuous MMS treatment (left) and IR (right) at the indicated doses. Bar plots represent corresponding mean ±SEM of n=3 independent replicates at the indicated MMS and IR doses. ** P-value < 0.01; * P-value < 0.05; ns, not significant (two-tailed Student's t test). (**H**) IF images and quantification of 53BP1 foci after 1 h 5 Gy IR treatment in WT HeLa cells transfected with siControl, siRNA #1 (left) or siRNA #2 (right) for RNF114. The mean ± SEM from 100 cells from a representative of 3 independent experiment is shown. **** P-value < 0.0001 (two-tailed Student's t test). Scale bars: 5 µm. (**I**) Representative IF images (left) and quantification (right) of γH2Ax foci after 5 Gy IR in HeLa WT cells transfected with siControl or siRNA #2 for RNF114. The mean ± SEM from 100 cells from a representative of 2 independent experiment is shown. ns, not significant (two-tailed Student's t test) Related to **Figure 7H**. (**J**) Representative IF images (left) and quantification (right) of RIF1 foci after 1 h 1 mM H_2O_2 treatment in WT or RNF114-KO U2OS cells. The mean ± SEM from 100 cells from a representative of 2 independent experiment is shown. * P-value < 0.05 (two-tailed Student's t test). Scale bars: 5 µm. Related to **Figure 7K**.
